# OpenDVP: an experimental and computational framework for community-empowered deep visual proteomics

**DOI:** 10.1038/s41592-026-03262-z

**Published:** 2026-10-22

**Authors:** Jose Nimo, Sonja Fritzsche, Daniela S. Valdes, Minh Trinh, Tancredi Pentimalli, Melissa Klingeberg, Simon Schallenberg, Frederick Klauschen, Florian Herse, Stefan Florian, Nikolaus Rajewsky, Fabian Coscia

**Affiliations:** 1 Max Delbrück Center for Molecular Medicine in the Helmholtz Association, Spatial Proteomics Group, Berlin, Germany; 2 Charité – Universitätsmedizin Berlin, corporate member of Freie Universität Berlin and Humboldt-Universität zu Berlin, Berlin, Germany; 3 German Cancer Consortium (DKTK), Partner Site Berlin, Heidelberg, Germany; 4 Humboldt-Universität zu Berlin, Institute of Biology, Berlin, Germany; 5 Laboratory for Systems Biology of Regulatory Elements, Berlin Institute for Medical Systems Biology, Max Delbrück Center for Molecular Medicine in the Helmholtz Association, Berlin, Germany; 6 Institute of Pathology, Charité – Universitätsmedizin Berlin, corporate member of Freie Universität Berlin and Humboldt-Universität zu Berlin, Berlin, Germany; 7 Institute of Pathology, Ludwig Maximilians University Hospital Munich, Munich, Germany; 8 German Cancer Consortium (DKTK), Partner Site Munich, German Cancer Research Center (DKFZ), Heidelberg, Germany; 9 Berlin Institute for the Foundations of Learning and Data, Berlin, Germany; 10 Max Delbrück Center for Molecular Medicine in the Helmholtz Association and the Charité – Universitätsmedizin Berlin, Experimental and Clinical Research Center, Berlin, Germany; 11 Max Delbrück Center for Molecular Medicine in the Helmholtz Association, Berlin, Germany; 12 Cluster of Excellence NeuroCure, Berlin, Germany; 13 German Center for Cardiovascular Research (DZHK) Partner Site Berlin, Berlin, Germany; 14 National Center for Tumor Diseases Partner Site Berlin, Berlin, Germany; 15 German Center for Child and Adolescent Health (DZKJ) Partner Site Berlin, Berlin, Germany; 16 ImmunoPreCept Cluster of Excellence, Berlin, Germany; 17 Einstein Center for Early Disease Interception, Berlin, Germany

**Keywords:** Proteomics, Image processing, Cancer imaging, Proteome informatics

## Abstract

Deep visual proteomics (DVP) is an emerging approach for cell-type-specific and spatially resolved proteomics. However, its broad adoption has been constrained by the lack of an open-source end-to-end workflow within a community-driven ecosystem. Here we introduce openDVP, an experimental and computational framework for accessible and scalable DVP. OpenDVP combines open-source image analysis tools (MCMICRO, QuPath and napari) with scverse formats (AnnData and SpatialData) for multiomics integration. It provides two workflows: a fast-track pipeline requiring no image analysis expertise, and an artificial intelligence-powered pipeline incorporating state-of-the-art methods for image preprocessing, segmentation and spatial analysis. We apply openDVP across three archival tissue studies (human placenta, early stage lung cancer and locally relapsed breast cancer), integrating spatial single-cell phenotypes with exploratory proteomic measurements. Finally, we introduce deep proteomic profiling of cellular neighborhoods as a scalable approach for spatial discovery proteomics.

## Abstract (web summary)

OpenDVP enables scalable and high-resolution proteomic profiling of cellular neighborhoods.

## Main

Recent advances in spatial omics have revolutionized our understanding of tissue biology and disease processes^1^. In particular, spatial proteomics is experiencing remarkable progress in sensitivity, throughput and spatial resolution^2^. The ability to map proteins with high spatial resolution has provided novel insights into cellular organization, tissue heterogeneity and disease mechanisms with unprecedented detail. The impact of these advancements lies in the premise that proteomic profiles provide a direct perspective on the functional and phenotype-centric cellular states that govern health and disease. Historically, spatial proteomics has depended on targeted, antibody-based methodologies^3^, which offer excellent spatial resolution, yet require prior knowledge for antibody panel design. Antibody-based methods, which generally profile up to ∼60 proteins^4^, capture a comparatively small fraction of the proteome, which is estimated to encompass more than 10,000 different proteins per single cell type^5^. A highly synergistic approach involves integrating targeted methods with ultrasensitive liquid chromatography (LC)–mass spectrometry (MS)-based proteomics. This multiscale approach has paved the way for exploratory, comprehensive analyses of cell type and spatially resolved tissue proteomes^6,7^. Recently, we codeveloped deep visual proteomics (DVP)^8^, which combines tissue imaging (immunofluorescence (IF) or immunohistochemistry (IHC)), machine learning-based image analysis, semi-automated laser microdissection (LMD) and ultrasensitive MS for the exploratory proteomic profiling of cell phenotypes. DVP enables the systematic mapping of thousands of proteins, elucidating their networks and signaling pathways within complex tissue architectures, and providing novel insights into health and disease states. For example, it recently enabled the discovery of a curative treatment strategy for a fatal human skin disease^9^ and mapped single-cell proteotoxicity in human liver^10^.

For image analysis, Biological Image Analysis Software^8^ has enabled full support of DVP experiments. However, broader adoption and methodological development have been limited by the absence of nonproprietary, open-source workflows that support scalable image analysis, LMD experimental design, multimodal data integration, and interoperability across imaging and omics platforms. These limitations have constrained systematic benchmarking, extensibility and community-driven DVP innovations.

To address these gaps, we developed openDVP, an open-source modular framework designed to complement and extend existing DVP solutions, including Biological Image Analysis Software. OpenDVP provides flexible, interoperable workflows that support simplified experimental strategies for users with limited computational expertise and advanced pipelines optimized for scalability and complex analyses. Built to integrate state-of-the-art bioimage analysis, spatial statistics and ultrasensitive MS-based proteomics, openDVP enables scalable acquisition and analysis of hundreds of spatially resolved proteomes. Across three archival tissue studies, we demonstrate the versatility of the framework and show that cellular neighborhood (CN)-guided proteome profiling offers a balanced alternative to technically more demanding single-cell isolation, enabling efficient spatial discovery proteomics at scale.

## Results

### Overview of the openDVP framework

**Fig. 1:**
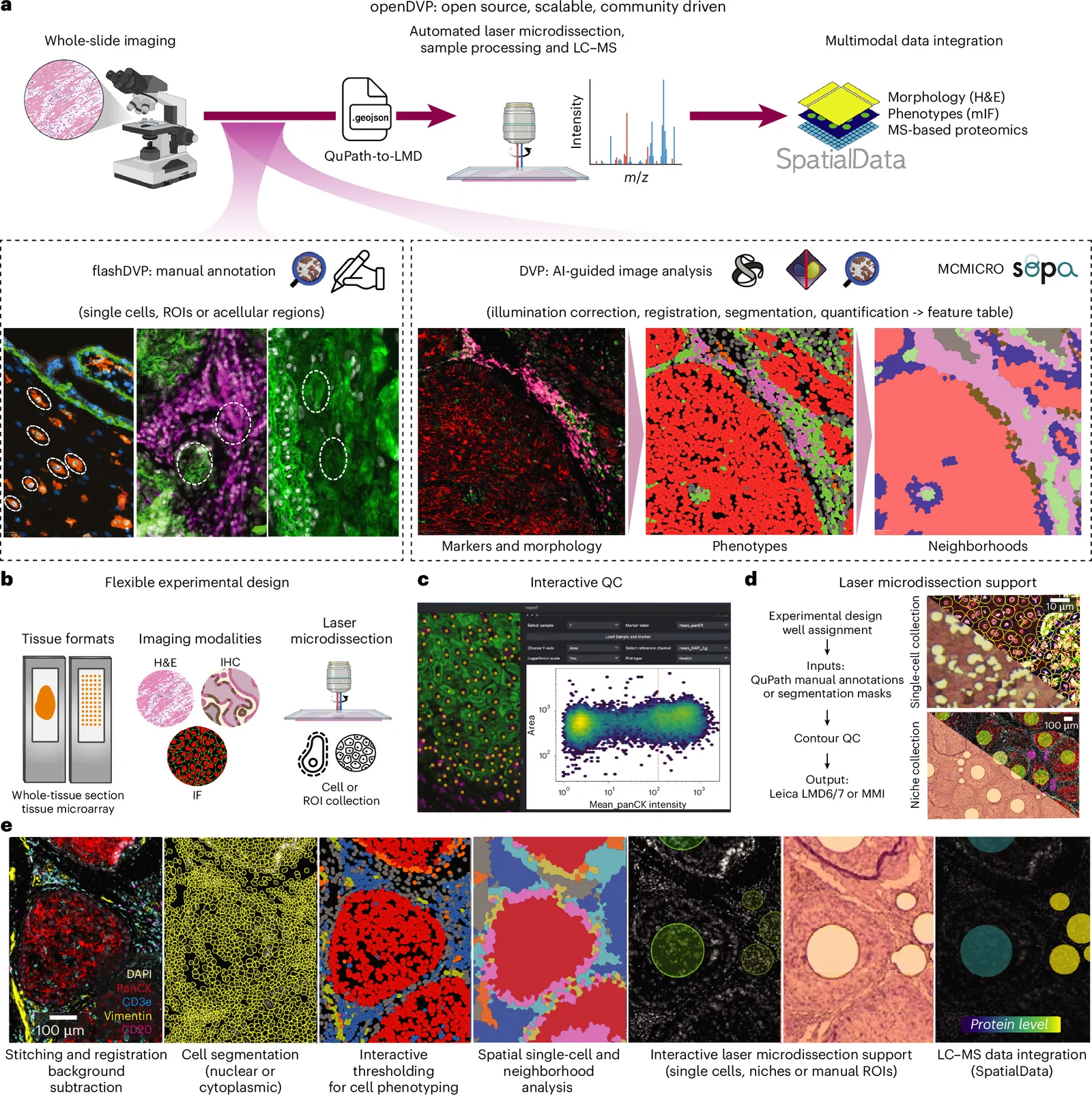
Overview of the openDVP framework. **a**, The main workflow of openDVP begins with tissue imaging to produce TIFF files. FlashDVP comprises manual image annotation, coordinate transfer with the QuPath-to-LMD web application and LMD. OpenDVP provides a framework to process images into feature tables and analyze matrices to phenotype cells, using open-source tools. These data layers provide the basis for contour export and LMD via QuPath-to-LMD. **b**, OpenDVP supports TMAs or WSIs, common imaging modalities and different LMD collection strategies. **c**, The napari-cell-gater plugin enables phenotyping through visual feedback thresholding, plotting cell features in fluorescence-activated cell sorting-like density plots. **d**, The QuPath-to-LMD web application allows users to design, transfer and validate annotations from QuPath and openDVP to an LMD-ready file format. **e**, An exemplary openDVP workflow showing the results of mIF imaging, cell segmentation, cell phenotyping, CN analysis, contour annotations and multimodal data integration using SpatialData. Illustrations in **a** and **b** created in BioRender; Coscia, F. https://biorender.com/89ddc1q (2026). AI, artificial intelligence.

We developed openDVP, an open-source modular framework for user-empowered DVP (https://github.com/CosciaLab/openDVP) (Fig. 1a). Our end-to-end framework encompasses optimized functions for all the key steps of the DVP workflow, supporting image preprocessing and analysis, automated LMD, interactive image data visualization and multimodal data integration. OpenDVP supports the most common imaging and proteomics data formats by current and extending functionalities from existing open-source software, such as MCMICRO^11^, SOPA^12^, QuPath^13^, napari^14^ and SpatialData^15^. Importantly, the modules’ inputs and outputs are standard file formats for seamless integration of community-published or in-house tools. We introduce two complementary workflows: a simplified fast-track pipeline, termed flashDVP, which does not require image analysis expertise, and an extended artificial intelligence (AI)-powered pipeline capable of in-depth spatial tissue analyses. FlashDVP serves as an intuitive entry point for users seeking to rapidly convert raw images (that is, hematoxylin and eosin (H&E), IHC or IF) into exploratory spatial proteomics results. The extended AI-powered DVP workflow enables more experienced users to customize and optimize the pipeline using advanced single-cell spatial analysis tools for more complex phenotype-to-proteotype associations.

### Efficient and extensible image processing

**Extended Data Fig. 1:**
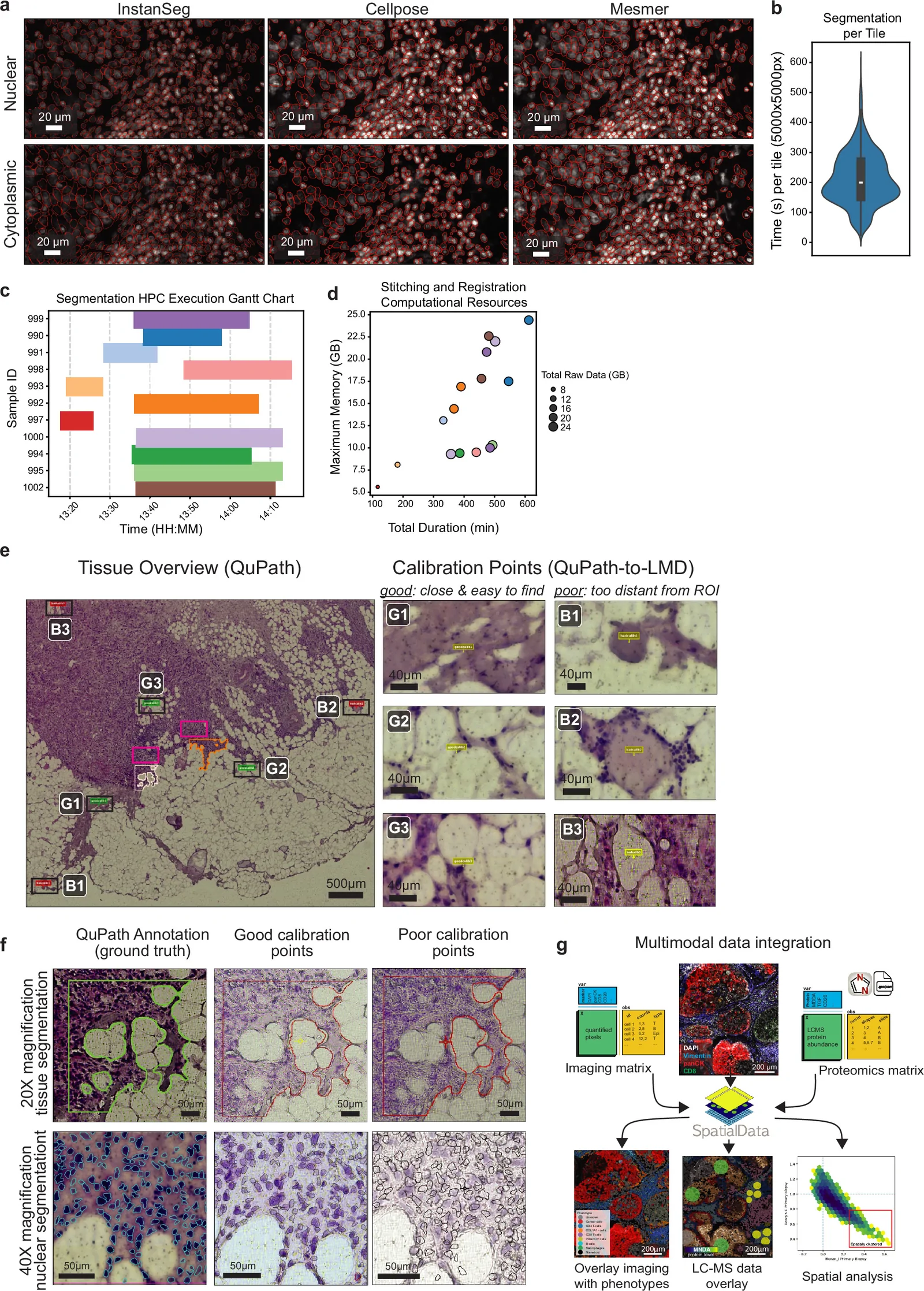
Benchmarking of computational resources and accurate reference point selection for reliable contour transfer for laser microdissection. (**a**) Comparison of segmentation approaches (InstanSeg, Cellpose and Mesmer). Shown is a subset of the TNBC tissue (primary biopsy), first nuclear stain. Top-row; Nuclear segmentation. Bottom-row; Cytoplasmic segmentation. Used InstanSeg’s “single_channel_nuclei-0.1.2” model for nuclear segmentation. For the cytoplasmic segmentation we used the “fluorescence_nuclei_and_cells-0.1.1” model on all channels. For Cellpose’s (v4.0.9) segmentation, we used the “cpsam” model. For nuclear segmentation we used the first nuclear stain and for cytoplasmic segmentation we used a two-channel (nuclei, membrane) maximum pixel projection. For Mesmer, (deepcell v0.12.10), we used the “nuc” compartment for nuclear segmentation, and “whole-cell” for the cytoplasmic segmentation (using the projection). (**b**) Violin plot showing duration in seconds of tile (5000x5000px) segmentation (total 2799 tiles across 11 whole-slide images). The central line indicates the median, the box represents the IQR (25th to 75th percentiles), whiskers extend to 1.5* IQR. For data see Suppl. Table 1. (**c**) Gantt chart showing parallelization capabilities of MCMICRO and SOPA, 11 whole-slides with a total of 6+ million cells were segmented in less than two hours. (**d**) Bubble scatterplot with proportional computational resources to input dataset size. Stitching and registration being the slowest process. (**e**) Tissue overview and reference point definition in QuPath. G1–G3 indicate good calibration points, located close to the objects of interest and easily identifiable. B1–B3 indicate poor calibration points, which are located farther away and are difficult to recall. The center shows four annotated regions: two magenta boxes for regions subjected to nuclear (H&E-based), and two regions of tissue annotations. Tests performed on a single tissue. (**f**) Accurate annotation transfer depends on reliable calibration points. Top row: large tissue segmentation annotation and its contour transfer to the LMD7. Poor calibration points result in an upward shift of the contour. Bottom row: nuclear segmentation and contour transfer. In this case, poor calibration points lead to relatively large positional errors, which result in improper single-cell collection. (**g**) Schematic illustrating how openDVP leverages the scverse SpatialData format to integrate antibody-based imaging, image-analysis–derived matrices, and LC–MS-based spatial proteomics data. This integration enables overlay of multiple information layers to guide laser microdissection and downstream spatial proteomics analyses.

Following image acquisition, the first step in the DVP workflow is the processing of images, including illumination correction and stitching image tiles. OpenDVP builds on top of the established open-source image processing pipelines MCMICRO and SOPA. MCMICRO was built using Nextflow^16^, following nf-core guidelines, to ensure high standards of modularity, consistency and interoperability. SOPA is built on Python-based Snakemake^17^, enabling Python-fluent users to add new modules or modify existing steps. We developed custom modules to expand segmentation masks by a defined number of pixels, quantify marker intensity quantiles per cell and pyramidize images for smooth quality control (QC). These open-source pipelines offer key advantages: (1) broad compatibility, supporting most imaging formats and modalities. (2) Modular architecture, integrating the latest benchmarked algorithms and machine learning models for cell and nuclear segmentation, such as Cellpose^18^, Mesmer^19^ and InstanSeg^20^ (Extended Data Fig. 1a). (3) Scalability, enabling efficient distribution of processes for stitching, registration and segmentation. For example, segmenting 11 whole-slide images (WSI) with a total of 6.34 million cells took less than 2 h (Extended Data Fig. 1b–d and Supplementary Table 1). (4) Active maintenance and development by a growing community of bioimage analysts.

### Providing a versatile solution to support diverse spatial proteomics experiments

We built openDVP for compatibility with a wide range of imaging applications that support WSIs and tissue microarrays (TMAs) based on conventional widefield or confocal microscopy (H&E, IHC, IF and multiplexed IF (mIF)) (Fig. 1b). While a WSI can provide a comprehensive view of large tissue areas, which are particularly important for heterogenous tissues, TMA applications support cohort-size spatial proteome analyses. Importantly, the imaging pipelines use Bio-Formats-compatible file formats (for example, OME-TIFF) and do not rely on any proprietary software or specific microscopy hardware for broad accessibility.

### Interactive QC and marker thresholding for single-cell phenotyping

Visual QC is critical in image processing pipelines to assess illumination correction, stitching and registration results, as well as segmentation accuracy. OpenDVP also provides demo datasets and Jupyter notebook-based tutorials to help users integrate and inspect results using two complementary interfaces, napari and QuPath. We chose them for their ability to visualize larger-than-memory images and community activity in developing new features. To facilitate image-based cell phenotyping based on marker expression, we developed an interactive napari plugin (github.com/CosciaLab/napari-cell-gater) (Fig. 1c). Our plugin enables users to visualize multichannel images, overlay segmentation masks, plot mutually exclusive markers (fluorescence-activated cell sorting analysis-like) and provide real-time feedback of cells being labeled positive or negative on the basis of selected thresholds. Although more laborious, this approach gives users full autonomy in their filter strategy for the complex task of single-cell phenotyping. For transparency, our plugin generates summary tables with cutoff values for each marker–sample pair. Finally, our image-processing pipeline creates a filtered single-cell matrix used for supervised or unsupervised analysis to identify cell types, states and CNs. These guide LMD and exploratory LC–MS-based proteome analysis.

### A versatile interface between image analysis and LMD

LMD is essential in the DVP workflow for isolating single cells or regions of interest (ROIs) precisely and semi-automatically. Segmentation-based cell/nucleus isolation provides the highest biological granularity, particularly relevant for single-cell applications or rare phenotypes. The isolation of larger ROIs (for example, multicellular niches) represents a robust alternative to single-cell LMD cutting. To support both scenarios, we developed QuPath-to-LMD, an open-source web application for experimental design, contour export and validation of LMD-ready masks, supporting the Leica LMD7 and MMI CellCut systems (Fig. 1d) (github.com/CosciaLab/Qupath_to_LMD). QuPath-to-LMD provides an interface between histopathology and omics-based profiling, as slide annotations can be directly transferred into LMD-ready contours for downstream (prote)omic analysis. We validated single-cell and ROI-based contour alignment on the LMD7 system using different magnifications (20× and 40×) and provided example images for selecting optimal tissue reference points (Extended Data Fig. 1e,f).

### Integrating imaging and MS-based proteomics data through the scverse ecosystem

DVP generates two types of proteomic data: imaging and LC–MS data. For data integration, visualization and multimodal analysis, openDVP’s Python code uses scverse standards and the AnnData and SpatialData formats, ensuring compatibility with popular analysis packages such as scimap^21^, scanpy^22^ and spatialproteomics^23^. This framework integrates MS-based proteomic data into the scverse ecosystem, streamlining data analysis for large-scale spatial tissue proteomics. For example, through SpatialData adoption, users can overlay their images with cellular phenotype information (for example, cell types and CNs) and matching quantitative proteome information. We developed a data analysis package to facilitate storing, integrating, visualizing and analyzing spatial proteomics data, available for download from the PyPi registry (Extended Data Fig. 1g). A vignette of the openDVP workflow is shown in Fig. 1e.

### OpenDVP quantifies cell-type-resolved proteomes of human placental tissue

**Fig. 2:**
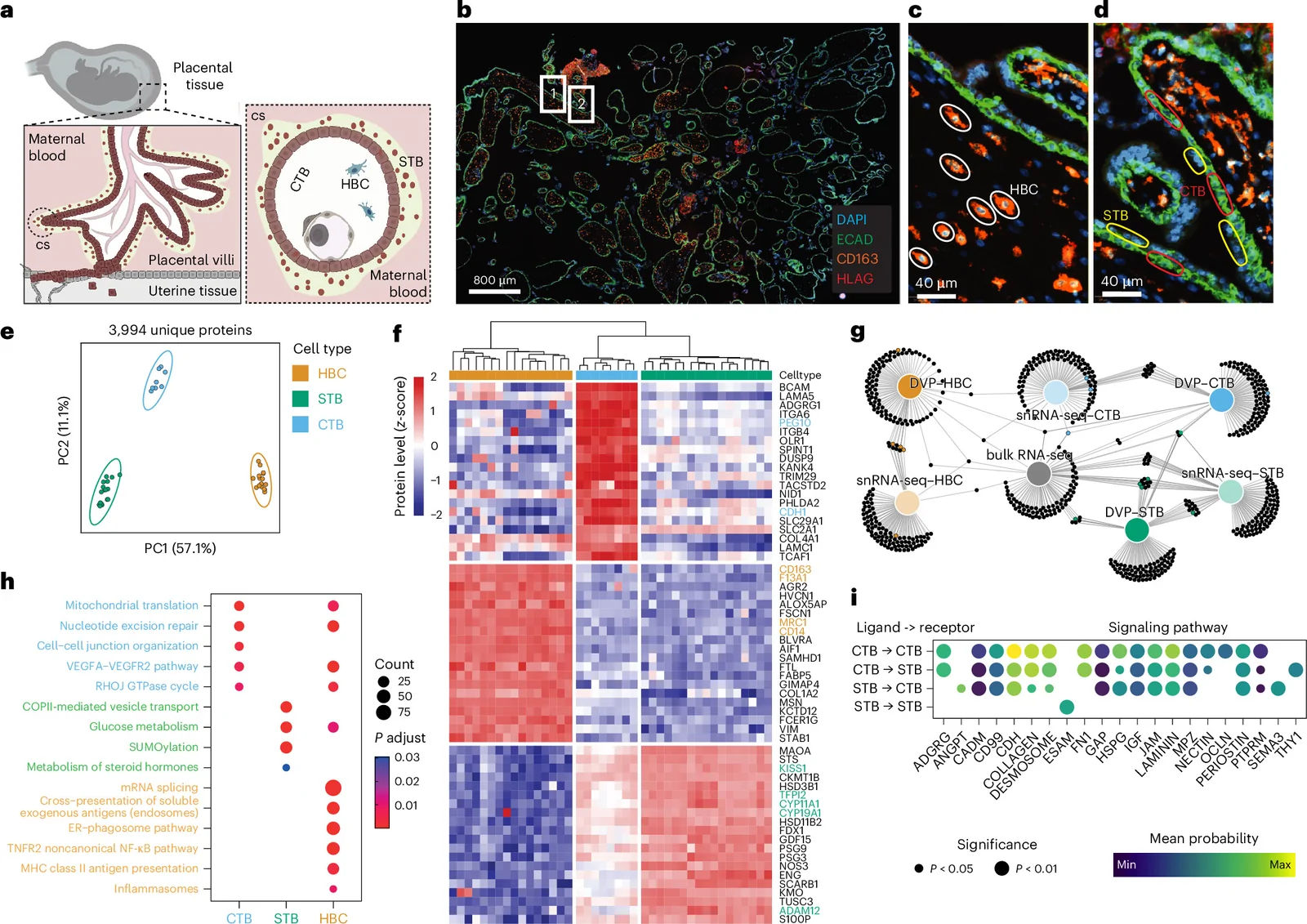
High-resolution cell-type-resolved proteomics of human placental tissue. **a**, Left: a schematic of placental villi samples and fetal structures at the maternal–fetal interface. Right: cross-section showing the CTB monolayer replenishing the STB layer, with HBC in the stroma. **b**, Representative IF image of placental tissue stained for E-cadherin, CD163, HLA-G and DNA (DAPI), used for cell type annotation, LMD and proteomic profiling. Box inserts correspond to **c** (1) and **d** (2). **c**,**d**, Representative ROIs manually selected in QuPath for isolation of immune (**c**) and trophoblast (**d**) compartments. The CTB monolayer is E-cadherin positive and HLA-G^−^, whereas the multinucleated STB layer is E-cadherin negative and HLA-G^−^ in the outer layer of the villi. CD163 expression marks macrophages (HBC). **e**, PCA of cell-type-resolved proteomes from three placental tissues. The colors indicate cell types. Confidence ellipses (95% confidence interval) were computed from principal component score covariance. **f**, A cell-type-specific protein marker heat map as inferred by ANOVA (FDR <0.05) showing relative abundance profiles per replicate. Canonical markers for each phenotype highlighted in orange (HBC), green (STB) and light blue (CTB). *z*-Scored relative protein abundances are shown. Distance metric: ‘euclidean’, method: ‘complete’. **g**, Comparison of flashDVP-generated phenotype-specific proteins and published genes marking CTB, STB and HBC phenotypes. Shared and unique markers highlight the importance of proteomic data in complementing other omics. **h**, A dot plot of enriched functional pathways of phenotype-separating clusters from **g**. Enrichment based on ranked proteins using the Reactome database. The colors show adjusted *P* values of overrepresented pathways, and dot size indicates category sizes (‘count’). *P* values were derived from a one-sided Fisher’s exact test with BH FDR correction. **i**, Significant cell–cell communication in the trophoblast compartment, inferred using CellChat^66^. The dot colors show mean communication probability of ligand–receptor pairs in the labeled pathways, and size represents the *P* value obtained from a one-sided permutation test. All samples depicted in **b**–**d** were imaged once. Illustrations in **a** created in BioRender; Coscia, F. https://biorender.com/ws6un4t (2026). cs, cross-section; *P* adjust, adjusted *P* value; snRNA-seq, single-nucleus RNA sequencing.

**Extended Data Fig. 2:**
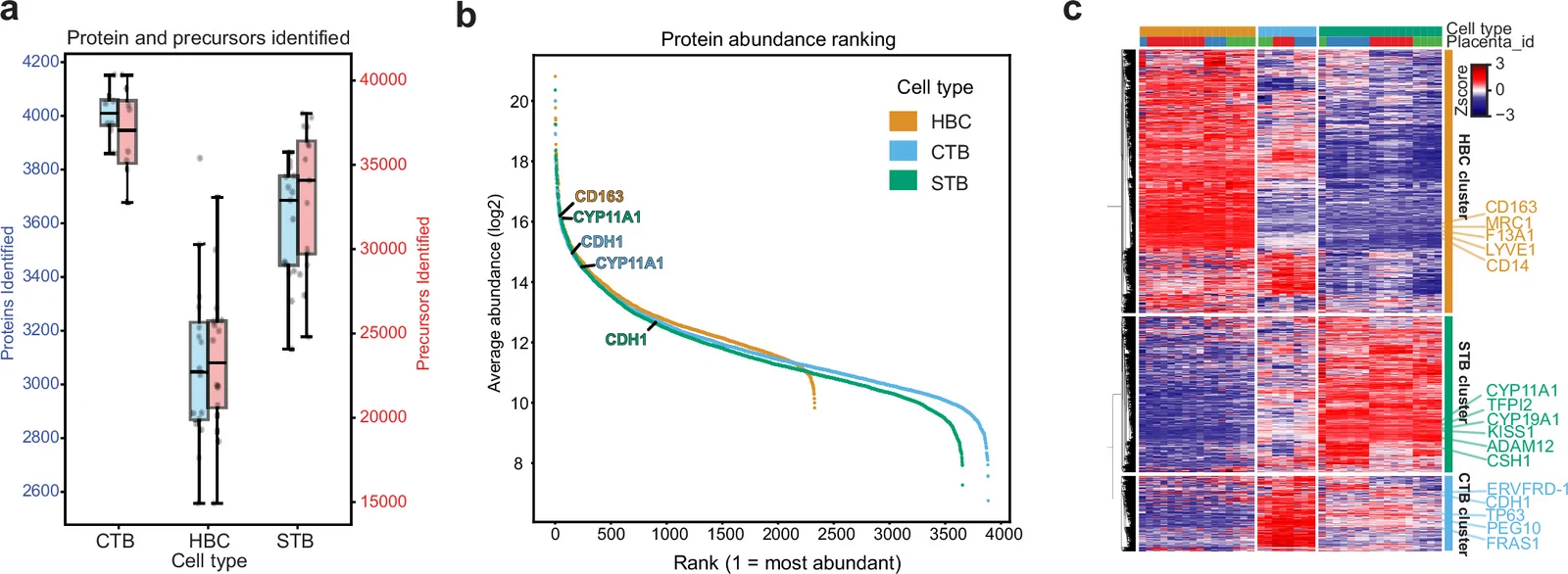
Proteome coverage and cell-type specific placental tissue proteomes. (**a**) Box plots showing the number of proteins (blue) and precursors (pink) identified per cell type; each dot represents a sample (CTB = 8, HBC = 16, STB = 17) from 3 biological samples; data available in Suppl. Table 2. The central lines indicate the median, the box the interquartile range, whiskers extend to (1.5*IQR) of the box. (**b**) Rankplot showing the dynamic range of proteins measured with labelled canonical markers and their rank for each cell type. (**c**) Unsupervised hierarchical clustering of 3,994 proteins, showing three distinct cell-type specific protein clusters used in Fig. 2h. Canonical markers of each cell type are labelled. The metric for clustering is “cityblock,” and the method is “average.” Clustering was performed by splitting the dendrogram into three largest groups. CTB, cytotrophoblast; STB, syncytiotrophoblast; HBC, Hofbauer cell.

Understanding human tissue biology and disease requires molecular characterization of individual cell types and states. Recent single-cell and spatial omics advancements have catalyzed large-scale initiatives, such as the Human Cell Atlas^24^ and the LifeTime Initiative^25^, which aim to systematically profile all human cell types across tissues to unravel relationships between cells, tissue organization and function. However, certain cell types (for example, cardiomyocytes, adipocytes and neurons) pose analytical challenges owing to their resistance to dissociation into single-cell suspensions. Their interconnectivity and/or fragility make them inaccessible for sorting-based single-cell profiling, and they are often morphologically too complex for accurate cell segmentation. One example is the syncytiotrophoblast (STB) of the human placenta, a syncytial layer derived from fused cytotrophoblasts (CTBs) that lacks distinct cell boundaries (Fig. 2a), making cell segmentation and single-cell-based LMD challenging. Despite STB’s critical roles in nutrient exchange, hormone secretion and immune modulation at the maternal–fetal interface^26^, its syncytial nature has prohibited its comprehensive molecular profiling, particularly at the global proteomic level. With this in mind, we applied flashDVP as a simplified and image segmentation-free version of DVP. We performed four-color IF staining of 5-µm-thick first-trimester human placental formalin-fixed paraffin-embedded (FFPE) sections mounted on PPS (polyphenylene sulfide) frame slides and performed centimeter-scale whole-slide imaging (Fig. 2b). The placental barrier, appearing in tree-like villi structures, controls the nutrient and gas exchange between maternal and fetal blood. It consists of E-cadherin-positive bipotential CTBs that fuse to form a multinucleated E-cadherin-negative, HLA-G–STB layer that is in direct contact with maternal blood. HLA-G^+^ trophoblasts constitute the invasive CTB-derived lineage that anchors the placenta to the maternal uterine tissue (Fig. 2a). CD163^+^ extraembryonic macrophages (Hofbauer cells (HBCs)) in the stroma play roles in tissue remodeling and immune surveillance, and support the formation and maintenance of fetal blood vessels in the villi^27^. With QuPath, we manually annotated 150 HBCs per replicate or an equal tissue amount for the STB and CTB layers (Fig. 2c,d). LMD contours were created using the QuPath-to-LMD web application, transferred to a Leica LMD7 microscope for LMD and processed using our ultralow-input tissue proteomics protocol^28^. Samples were measured in label-free dia-PASEF mode and analyzed with DIA-NN^29^ in library-free mode. We identified 3,994 unique proteins across groups (Extended Data Fig. 2a and Supplementary Table 2), including canonical markers for each cell type (Fig. 2d and Extended Data Fig. 2b,c). Principal component analysis (PCA) separated cell phenotypes regardless of tissue origin (Fig. 2e). Differential abundance analysis revealed cell-type-specific markers that showed good concordance with public transcriptomics data^30,31^ (Fig. 2f,g and Supplementary Table 3). Pathway analysis of protein clusters confirmed STB-specific functions in steroid hormone metabolism, vesicular transport and energy demands (Fig. 2h). The regulation of SUMO proteins, linked to placental development and hypertension in pregnancy in bulk studies^32,33^, was specific to STBs despite their lower abundance, underscoring the value of cell-type-resolved proteomic analyses. CTB and HBC showed an enrichment in RhoJ GTPase and VEGF signaling pathways, while HBCs displayed immune regulation pathways, supporting their immunotolerant role. We found HBC-specific enrichment for proteins involved in mRNA splicing, aligning with the role of alternative splicing as an essential feature of placental dysfunction^34^. Given the physical proximity between STBs and CTBs (Fig. 2a,d), cell communication analysis between these connected trophoblasts cells revealed high paracrine activity of both cell types, with autocrine communication strongest in the CTB monolayer (Fig. 2i and Supplementary Table 3). Strong signaling via cadherins, junctional adhesion molecules, fibronectins (FN1) and desmosomes was inferred, supporting the importance of cell adhesion integrity in fusion-competent CTBs. We identified robust communication involving collagens, laminins and extracellular matrix proteins critical for implantation and placentation^35^. Our data also revealed an endothelial cell-selective adhesion molecule (ESAM) as a unique and previously unreported autocrine signaling molecule of STBs. ESAM is an important component of tight junctions and a negative regulator of platelet activation; the latter is known to underpin pregnancy health and coexist with complications^36^. These findings establish flashDVP as a powerful ‘fast-track’ approach for cell type and spatially resolved tissue proteomics.

### OpenDVP coupled to spatial transcriptomics uncovers niche-specific drug targets

**Fig. 3:**
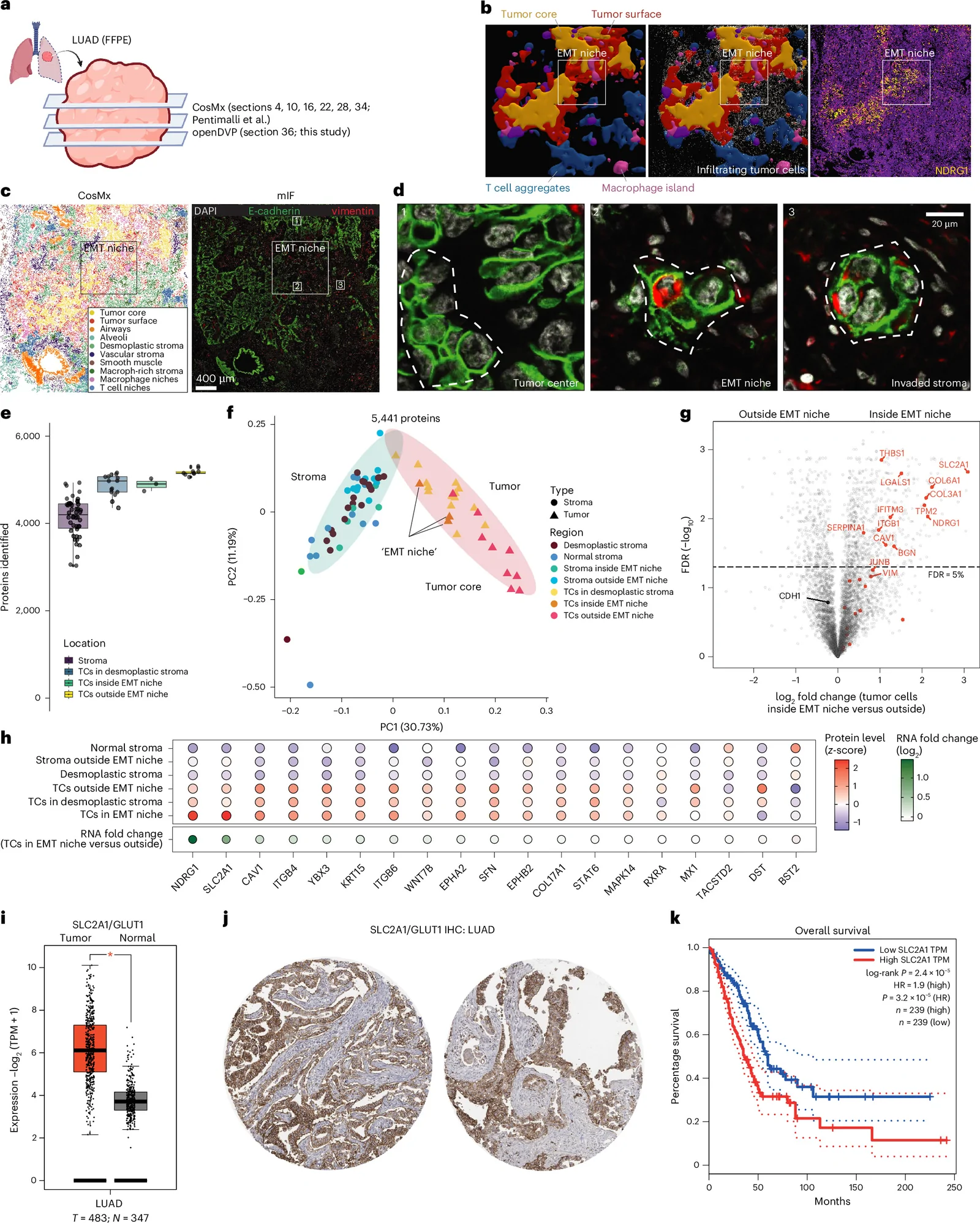
Cell-type-resolved deep proteomic profiling of transcriptionally defined cellular niches. **a**, An overview of 5-µm FFPE LUAD sections showing regions for CosMx ST and DVP analysis. **b**, A 3D reconstruction of transcriptomically defined niches, showing tumor-infiltrating cells. The *NDRG1* RNA level was upregulated in tumor cells from the surface and EMT niche, as previously published by us^37^. **c**, Left: two-dimensional multicellular niche map from CosMx data (section 34 in ref. ^37^). Right: IF of epithelial (E-cadherin, green) and mesenchymal (VIM, red) markers in section 36. Insets 1–3 correspond to **d**. Nuclei stained with DAPI (white). Scale bar, 400 µm. Imaging was performed once. **d**, Magnified views: (1) tumor center (E-cadherin positive/VIM^−^), (2) EMT niche (E-cadherin positive/VIM^+^) and (3) invading tumor cells (E-cadherin positive/VIM^+^). Scale bar, 20 µm. **e**, Proteomics identified a mean of 4,138 proteins in stroma and 4,976 in tumor regions (50–100 cells per sample). **f**, PCA of 5,441 proteins shows a separation of tumor and stroma samples along PC1. EMT niche tumor samples cluster near invasive and stromal samples. **g**, A volcano plot showing differential protein abundance in tumor cells inside versus outside the EMT niche. CosMx-upregulated genes^37^ are shown in red. Proteins above the dashed line have a permutation-based FDR below 0.05. **h**, Relative protein levels of the top upregulated genes in EMT niche tumor cells. **i**, Mean RNA expression of *SLC2A1*, the most EMT niche upregulated tumor protein in the proteomics data, in the lung cancer cohort (*n* = 483 tumor, 347 healthy; TCGA LUAD) shows higher expression in cancer tissues^41^. **j**, GLUT1 IHC staining of LUAD tissues confirmed tumor-specific expression. Images (left, ID 426; right, ID 1303) from the human protein atlas^43^. **k**, Kaplan–Meier analysis showed lower overall survival in patients with high *SLC2A1* RNA expression, grouped by median. The dotted lines represent the 95% confidence intervals. For box plots in **e** and **i**, the central lines indicate the median, the box indicates the interquartile range (IQR) and whiskers extend to 1.5× IQR of the box. Illustrations in **a** created in BioRender; Coscia, F. https://biorender.com/89ddc1q (2026). TC, tumor cell; Macroph, macrophage; TPM, transcripts per million; HR, hazard ratio.

**Extended Data Fig. 3:**
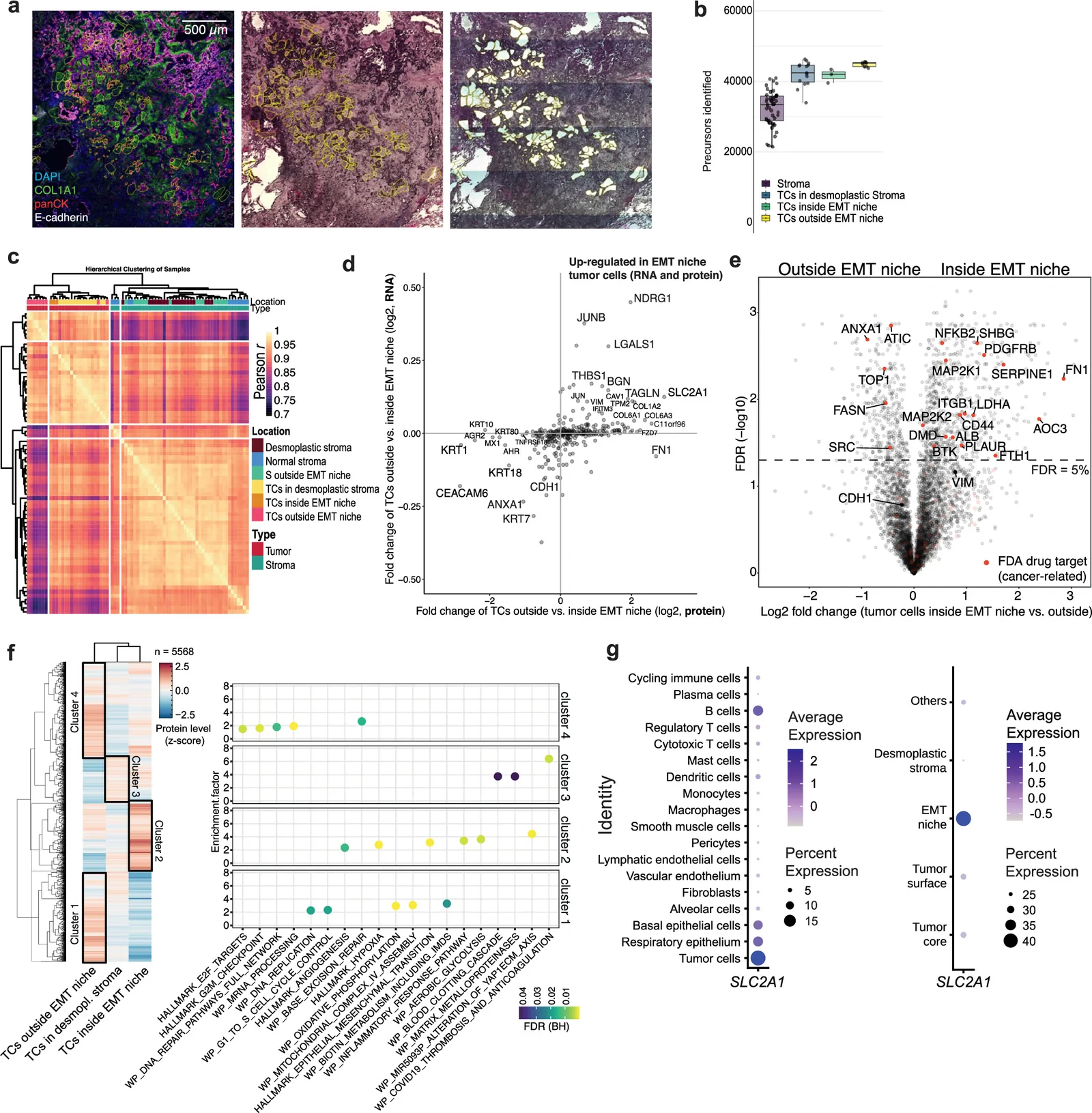
Integration of spatial transcriptomics and proteomics in lung cancer. (**a**) Imaging modalities (H&E and IF) used to guide laser microdissection. Yellow annotations show ROIs used for proteomic profiling. Left: Multiplex immunofluorescence used to locate tumor and stromal niches identified by spatial transcriptomics. H&E staining showing ROIs before (middle panel) and after LMD collection (right panel). Each imaging took place once for each tissue. (**b**) Boxplot showing the number of identified precursors per sample type. The central lines indicate the median, the box the interquartile range, whiskers extend to (1.5*IQR) of the box. (**c**) Pearson’s correlation heatmap for all analyzed samples, method: “complete”, distance metric: “euclidean”. (**d**) Scatterplot comparing fold changes between tumor cells inside and outside the EMT niche, for both spatial proteomics and spatial transcriptomics. (**e**) Volcano plot showing differentially abundant proteins between tumor cells inside and outside the EMT niche as defined by CosMx spatial transcriptomics. FDA-approved drug targets are highlighted in red. E-Cadherin (CDH1) and Vimentin (VIM) are shown for reference. (**f**) Heatmap showing clusters of proteins between tumor cells inside the EMT niche, outside the EMT niche, and in the desmoplastic stroma, method: “complete”, distance metric: “euclidean”. The dot plot shows enriched Hallmarks and WikiPathways pathways for the different clusters using ClusterProfiler R package 71. (**g**) Dot plot of SLC2A1 expression across all cell types (left) and in tumor cells distributed in different niches (right). Dot size indicates the percentage of gene-expressing cells; color represents average expression (log-normalized and scaled).

We next tested whether flashDVP could facilitate cell-type-resolved tissue proteomics by integrating additional spatial omics layers. We analyzed a serial FFPE tissue section from an early stage lung adenocarcinoma (LUAD) that we previously characterized by spatial transcriptomics (ST; Nanostring CosMx)^37^ (Fig. 3a). Using 960 cancer-related genes, we profiled over 340,000 cells, identifying 18 cell types and their spatial distribution. These rich data revealed diverse immune and tumor cell niches of therapeutic value, including a small tumor niche enriched for prometastatic mesenchymal tumor cells with high *NDRG1* and *LGALS1* expression, located near myofibroblasts and SPP1^+^ tumor-associated macrophages (Fig. 3b). Pseudotime analysis identified this ‘epithelial-to-mesenchymal (EMT) niche’ as the potential start site of tumor invasion. We used the CosMx data to guide the collection of the EMT niche tumor cells, and revealed their proteomes to understand their functional state and identify potential drug targets. IF staining of a serial tissue section against epithelial and mesenchymal markers, overlaid with CosMx phenotypes, confirmed the mesenchymal nature of EMT niche tumor cells on protein level (panCK^+^ and vimentin (VIM)^+^) (Fig. 3c,d). We next annotated multiple ROIs to proteomically profile tumor and stromal niches (Extended Data Fig. 3a). ROIs contained 50–100 cells (40,000–50,000 µm^2^, 5-µm-thick). We quantified 4,138 and 4,976 proteins in stromal and tumor compartments, respectively, totaling 5,956 proteins (Fig. 3e, Extended Data Fig. 3b and Supplementary Table 4). Group replicates clustered together and showed high proteome correlations (Pearson’s *r* > 0.9; Extended Data Fig. 3c). PCA analysis showed that EMT niche tumor cells and stroma-infiltrating tumor cells were closer to stromal samples in component 1, reflecting their mesenchymal phenotype (Fig. 3f). Integration with CosMx data confirmed that tumor cell-intrinsic signatures dominated the EMT niche tumor proteomes (Fig. 3g,h). Proteomics further validated the CosMx and IF data showing panCK^+^, VIM^+^ and E-cadherin-negative tumor cells in the EMT niche and upregulated NDRG1 and LGALS1 protein levels. Many genes identified by ST were also significantly upregulated at the protein level in EMT niche tumor cells, emphasizing the value of spatial niche information in three dimensions (3D) for aligning and integrating tissue sections for multiomics analyses (Fig. 3a). However, MS-based proteomics captured higher dynamic ranges of analyte abundances, resulting in different gene/protein rankings and drug target prioritizations (Fig. 3g,h and Extended Data Fig. 3d). We identified 180 Food and Drug Administration-approved drug targets, of which 16 cancer-related genes were significantly upregulated in EMT niche tumor cells (Extended Data Fig. 3e and Supplementary Table 4). For example, *PDGFRB*, an actionable gene for imatinib, a receptor tyrosine kinase inhibitor approved for various malignancies, including gastrointestinal stromal tumors and myeloproliferative neoplasms^38^. GLUT1, encoded by *SLC2A1*, was the most upregulated protein in EMT niche tumor cells. Consistently, pathway analysis revealed high glycolysis expression in EMT niche tumor cells, along with hypoxia, angiogenesis and EMT pathways (Extended Data Fig. 3f). As targeting glucose metabolism is currently under clinical investigation for cancer therapy^39,40^, we assessed the tumor-specific expression of *SLC2A1* and its association with patient outcomes. We validated high tumoral *SLC2A1* expression in a large-scale transcriptome study of 483 LUADs and 347 control samples^41,42^ (Fig. 3i), as well as on IHC data obtained from the human protein atlas^43^ (Fig. 3j). High *SLC2A1* expression was a strong prognostic factor associated with poor patient outcome, as revealed by the significantly different overall survival (*P* = 0.0003, *n* = 239 per group) (Fig. 3k). Notably, re-evaluation of the ST data not only confirmed the tumor-specific expression of *SLC2A1* but also emphasized its strong EMT niche specificity (Extended Data Fig. 3g). Together, these results underline the benefit of cellular niche-guided spatial multiomics as a promising approach for identifying and validating niche-specific, personalized drug targets.

### OpenDVP reveals tumor microenvironment remodeling in primary and relapsed TNBC

**Fig. 4:**
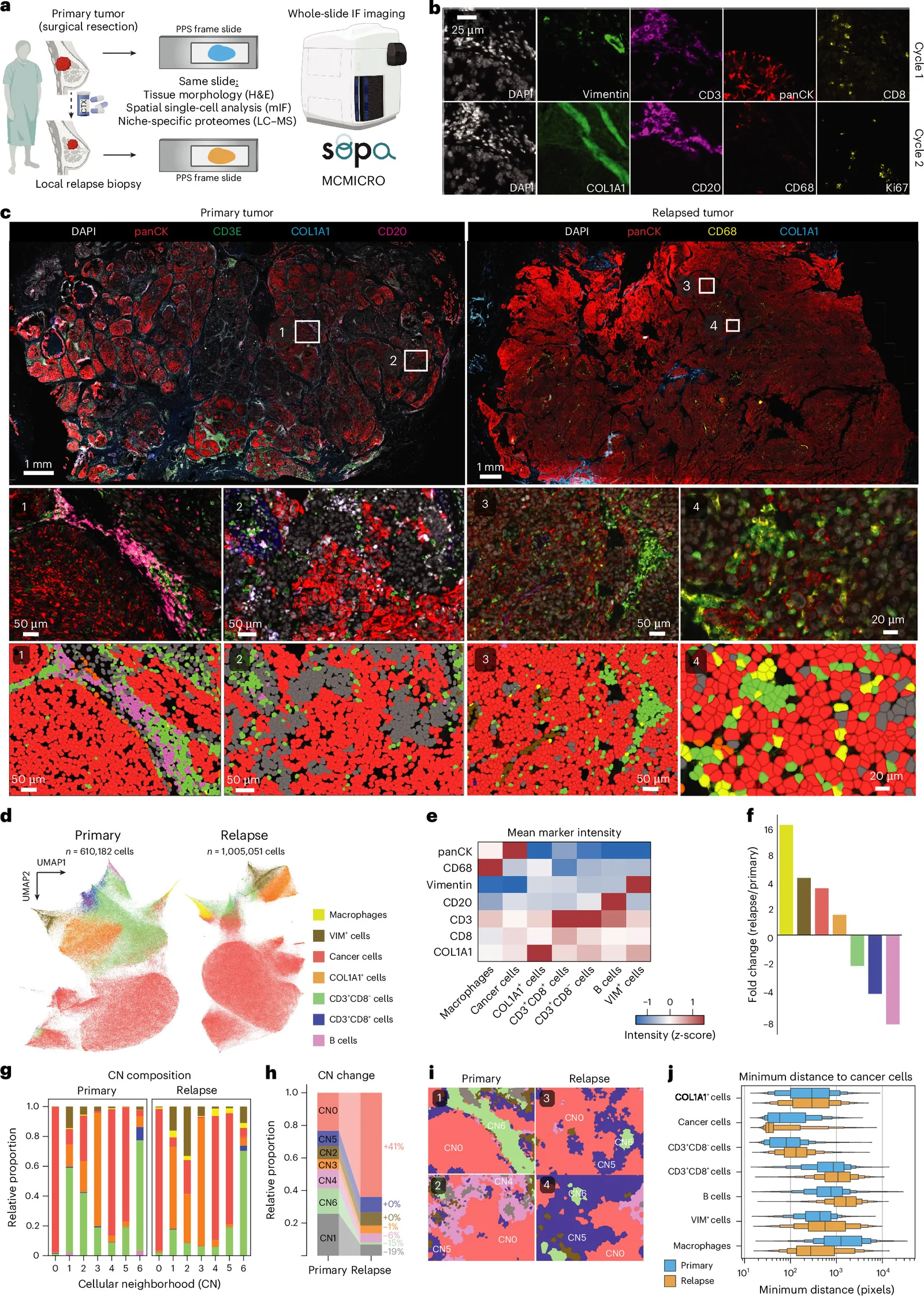
Image acquisition and analysis of primary and recurrent TNBC. **a**, FFPE 5-µm sections of a sample from a primary tumor and the same patient post-chemotherapy relapse were mounted on PPS frame slides. **b**, Slides underwent mIF staining and processing using MCMICRO and SOPA pipelines. **c**, Whole-slide mIF images of samples with zoomed regions show overlaid cell phenotype information. **d**, Uniform manifold approximation and projection (UMAP) embeddings of segmented cells from each slide, based on morphology and marker intensity (excluding nuclear stains and background); unidentified cells were excluded; n_neighbors = 25. **e**, A heat map of *z*-scored mean marker intensities across phenotyped cells. Phenotyping was performed by thresholding markers per slide, rescaling intensities and scimap phenotype function. **f**, Fold change in cell type counts between recurrent and primary samples. **g**, Relative composition of CNs per sample, excluding unidentified cells. **h**, Selected regions with CNs overlaid. **i**, Stacked bar plots showing CN frequency in each sample, calculated using scimap’s spatialLDA function. **j**, Boxen plots of the minimum distance from cells to the nearest cancer cell between primary and relapse samples (for cell numbers see Supplementary Table 5). The central line indicates the median, the thickest box represents the IQR (25th–75th percentiles), and each subsequent thinner level of boxes corresponds to half the remaining data points. The outermost lines (representing the whiskers) extend to the absolute minimum and maximum observed values. Imaging for **b** and **c** was performed once. Illustrations in **a** created in BioRender; Coscia, F. https://biorender.com/89ddc1q (2026).

**Extended Data Fig. 4:**
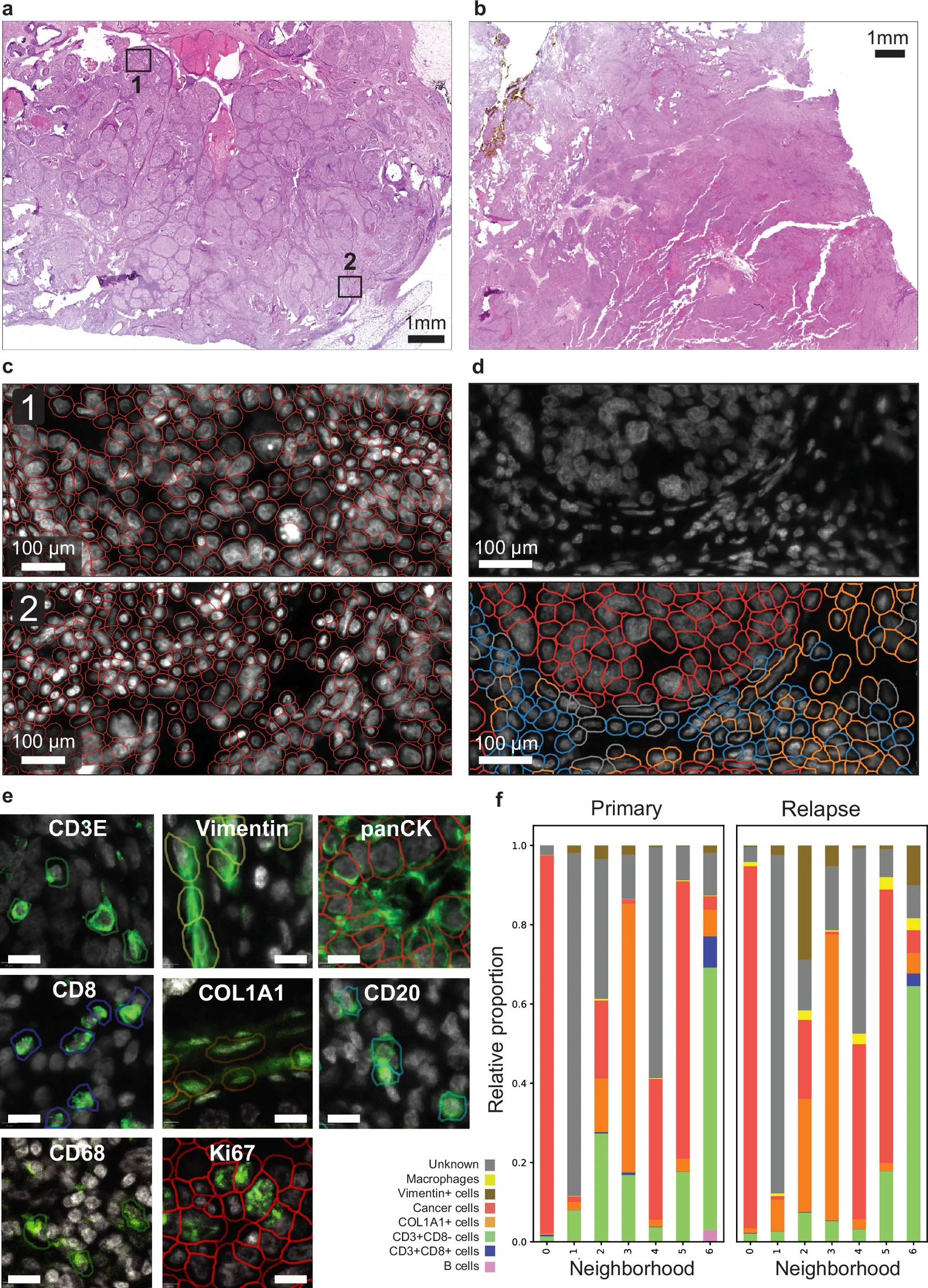
Consistent segmentation across large distances and staining variations. (**a**) H&E image of the primary TNBC tissue. (**b**) H&E image of the relapse TNBC tissue. Both tissues showed minor tissue losses due to cyclic imaging on PPS frame slides. (**c**) Nuclear stain overlayed with segmentation mask. These zoom-ins belong to distant regions from the primary TNBC tissue, areas shown and numbered in plot (**a**), showing segmentation consistency across entire whole-slide. Each tissue underwent mIF imaging once and then H&E staining and imaging. (**d**) Zoom-ins of the primary tissue. The white color shows DAPI as the nuclear counterstain. Bottom image shows segmentation masks coloured by assigned phenotype labels. (**e**) Exemplary images from same imaging experiment for each IF marker used, overlaid with segmentation mask. Colors represent phenotyped cells for each marker (not for Ki67). Scale bar represents 40 µm. (**f**) Barplots with cellular neighborhood compositions for each sample, showing the proportion of unclassified cells in grey. Related to Fig. 4g.

Current DVP implementations enable the integration of visually and machine learning-defined cell phenotypes with quantitative proteomic profiles but involve trade-offs between spatial resolution, proteome depth and experimental throughput. Our original DVP concept^8^ achieves workflow robustness and high proteome coverage by pooling phenotypically coherent cell populations, whereas single-cell DVP (scDVP)^44^ maximizes spatial resolution at the expense of analysis depth and ease of use. These limitations motivate alternative strategies that preserve spatial interpretability while substantially increasing throughput and discovery potential. Recent advances in spatial biology have established cellular neighborhoods (CNs) as fundamental organizational units that shape tissue function, disease progression and therapeutic response^45–47^. Triple-negative breast cancer (TNBC), an aggressive breast cancer subtype^48^, exemplifies how CN organization within the tumor microenvironment correlates with clinical outcomes^49^, including critical roles for macrophage- and fibroblast-enriched niches^50–52^. Resolving CN-specific proteomes therefore offers an opportunity to identify molecular mediators of cell–cell interactions and disease progression. To enable CN-resolved proteome profiling, we first established an optimized protocol for centimeter-scale whole-slide mIF imaging on PPS membrane slides, using a nine-marker panel for this proof-of-concept study (Fig. 4a,b). We analyzed paired TNBC tissue sections from the primary surgical resection and a matched local relapse obtained 2 years after adjuvant chemotherapy. The primary specimen contained extensive ductal carcinoma in situ (DCIS; 38 mm) with focal invasion into the surrounding stroma, whereas the relapse consisted of a large (45 mm) invasive carcinoma without residual DCIS (Fig. 4c). Each WSI stack (>150 GB) was processed using MCMICRO and SOPA for cell segmentation, phenotyping, and neighborhood analysis, with matched H&E sections providing additional morphological context (Extended Data Fig. 4a–d). We segmented 610,182 and 1,005,051 cells from the primary and relapse samples, respectively, identifying five major cell types: tumor, T cells, B cells, macrophages and stromal cells (Fig. 4c–e and Extended Data Fig. 4d,e). While panCK^+^ tumor cells dominated both tissues, marked shifts in immune and stromal composition were observed (Fig. 4f and Supplementary Table 5). Compared with the primary tumor, the relapse exhibited pronounced depletion of B cells and CD8^+^ T cells alongside a 16-fold increase in CD68^+^ macrophages and a 4-fold expansion of VIM^+^ stromal cells, consistent with immune evasion and tumor progression^53,54^. CN analysis using scimap’s spatialLDA identified seven recurrent CNs (CN0–6) with distinct cellular compositions (Fig. 4g, Extended Data Fig. 4f and Supplementary Table 5). Several CNs showed strong abundance differences between the two samples (Fig. 4g–i), accompanied by spatial reorganization of immune cells. Proximity analysis revealed that macrophages were located closer to tumor cells in the relapse, whereas lymphocytes preferentially surrounded DCIS regions in the primary tumor (Fig. 4j), indicating a transition from a lymphocyte-rich peritumoral niche to a macrophage-dominated intratumoral microenvironment.

### cnDVP enhances proteome coverage and scalability

**Fig. 5:**
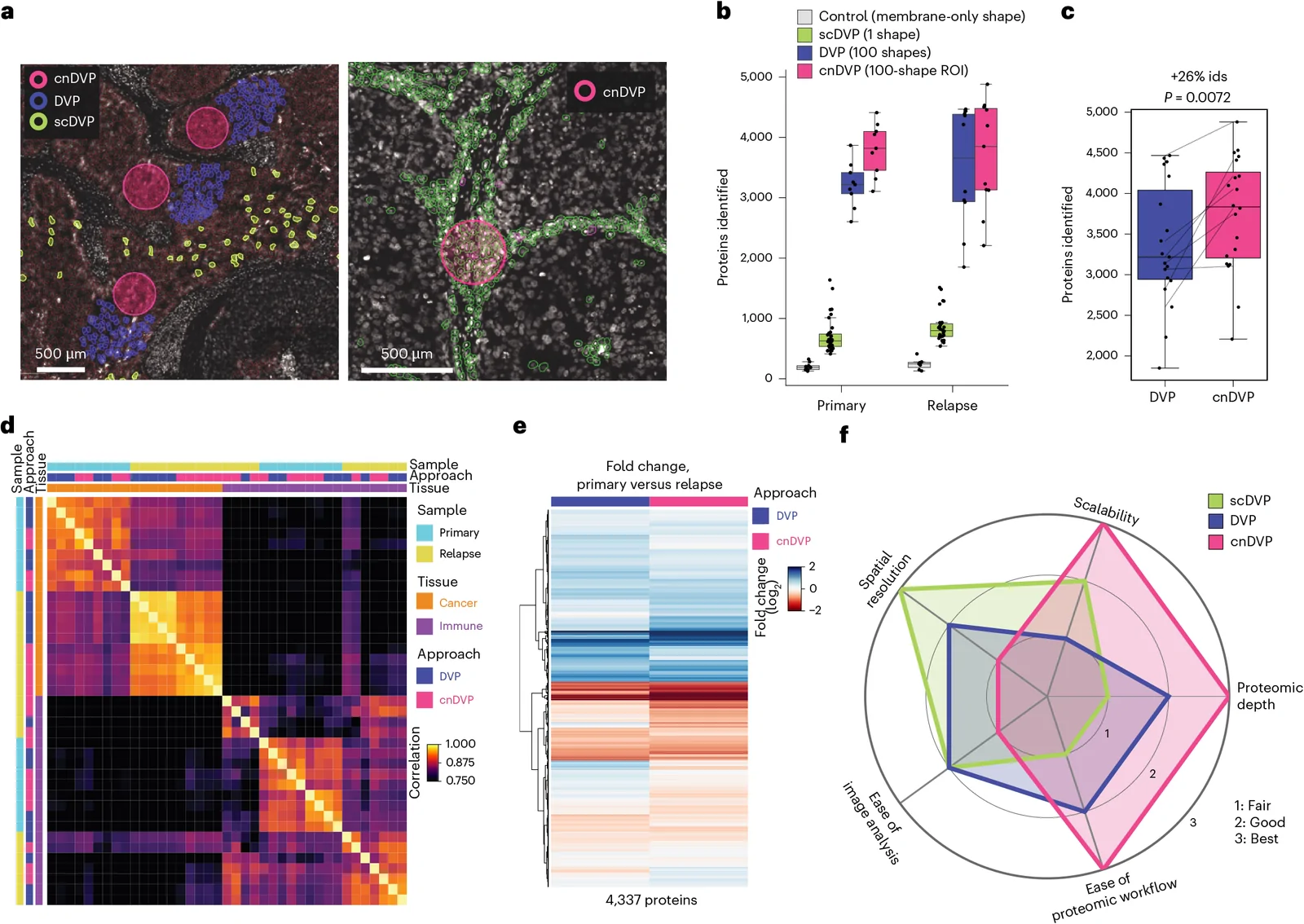
cnDVP improves proteomic depth and throughput. **a**, Images of primary TNBC tissue, showing the nuclear stain with segmentation masks for phenotyped cancer cells with red outlines on the left and CD3^+^ cells with green outlines on the right. Overlayed are collection annotations for the three DVP approaches; in magenta cnDVP, in blue DVP and in green scDVP. **b**, Box plots with the number of proteins identified with DIA-NN search across DVP approaches. **c**, A box plot with the number of proteins identified in matching cancer-specific samples analyzed by DVP and cnDVP. The lines denote spatial colocalization, showing an increase of proteome coverage using the cnDVP approach. The *P* value is based on a two-sided paired *t*-test between paired samples. For both **b** and **c**, the central lines indicate the median, while the box represents the IQR (25th–75th percentiles) and whiskers extend to 1.5× IQR of the box. Source data are provided in Supplementary Table 6. **d**, Proteome correlation heat map using Spearman correlations for all immune and cancer samples analyzed with DVP and cnDVP. Metric: ‘euclidean’, method: ‘ward’. **e**, A heat map of log_2_ fold change values for cancer samples between the primary and relapse breast cancer tissues across DVP and cnDVP approaches. Note that similar fold changes were retrieved for both approaches. **f**, The radar plot summarizes the key differences between three DVP approaches: scDVP (light green), DVP (blue) and cnDVP (pink); across five key properties: scalability, proteomic depth, ease of proteomic workflow, ease of image analysis and spatial resolution. The radial axes represent a qualitative ranking scale where 1, ‘fair’; 2, ‘good’; and 3, ‘best’. ids, identifications.

**Extended Data Fig. 5:**
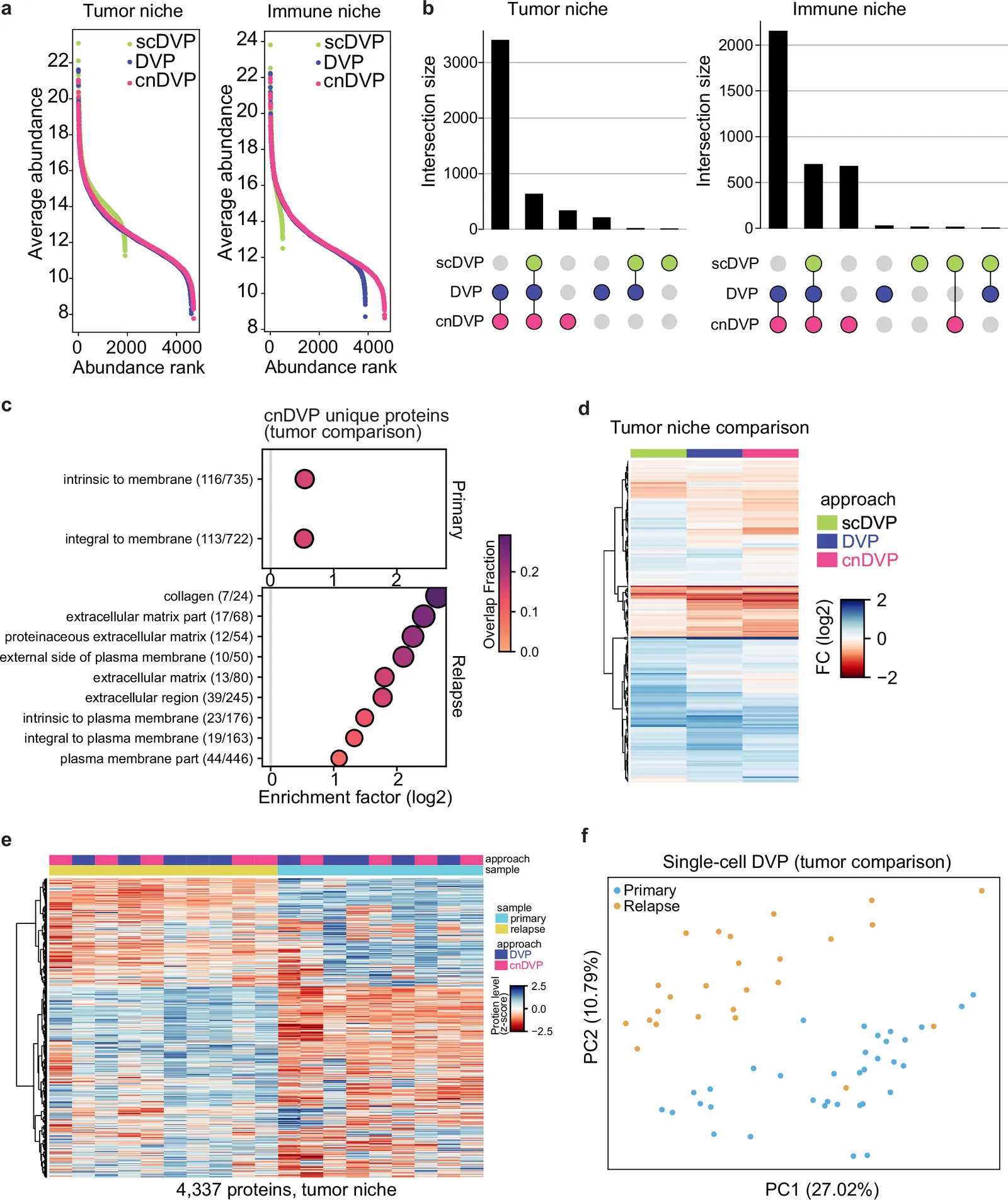
Comparative proteomic performance of complementary DVP strategies. (**a**) Rank plots illustrating the dynamic range of identified protein groups across three DVP approaches. Protein groups were retained if detected in at least five and three valid values for tumor and immune niches, respectively. (**b**) UpSet plots showing overlap of protein groups identified by the three DVP approaches. Only protein groups with at least 60% valid values per group are shown. (**c**) Dot plots represent the most significantly enriched Gene Ontology Cellular compartment (GOCC) terms for cnDVP specific proteins. Primary tissue (upper panel) and relapse tissue (lower panel). Unique proteins were determined by comparing missingness between DVP and cnDVP samples of directly adjacent cells with 504 unique proteins for the primary tumor analysis and 226 for the recurrent tumor. Proteins with 40% or more missing values per group (DVP or cnDVP) were filtered out. A Fisher’s exact test was used with a Benjamini-Hochberg FDR cut-off of 0.01. Color code and size of dots show the fraction of intersecting proteins relative to the total proteins.(**d**) Heatmap of log_2_ fold change values between primary and relapse cancer samples for the three DVP approaches. Protein groups were stringently filtered to retain features with at least 70% valid values in each DVP approach. Missing values were imputed from a down-shifted Gaussian distribution of lower-intensity values (mean shift −1.8, standard deviation 0.3, calculated per feature). Hierarchical clustering was performed using Euclidean distance and the Ward linkage method. (**e**) Heatmap of log_2_-transformed protein abundances in cancer samples profiled by the DVP and cnDVP approaches. Protein groups were retained if they contained at least 70% valid values in either the primary or relapse sample group. Missing values were imputed as in (**d**), and values were z-scored separately for the DVP and cnDVP approaches. Unsupervised hierarchical clustering was based on Euclidean distance and the Ward method. (**f**) Principal component analysis (PCA) of single-cell DVP (scDVP) cancer samples, colored by primary or relapse status. Protein groups were filtered to retain those with at least 50% valid values in either group (923 protein groups total). Missing values were imputed as described in (**d**).

Motivated by these spatial differences, we hypothesized that CN-guided DVP (cnDVP) could enable scalable spatial proteomics while preserving the core DVP principle of linking visual phenotypes to quantitative proteomic profiles. To systematically evaluate this approach, we compared cnDVP with conventional DVP^8^ and scDVP^44^ using matched regions from the primary and relapse TNBC tissue. To minimize spatial confounders, we profiled directly adjacent cells of identical phenotype and CN assignment (Figs. 4e,g and 5a). For DVP, we excised 100 individual cell shapes per well, whereas cnDVP required only a single shape encompassing 100 phenotyped cells, resulting in an approximately 100-fold faster tissue collection. For scDVP, we collected individual tumor or immune cells. As cell size largely determines proteome coverage^55^, scDVP has remained challenging for small cell types such as immune cells (Extended Data Fig. 5a). From 50 single immune cells (CD3^+^/CD8^−^ lymphocytes or CD68^+^ macrophages) profiled, only 3 samples remained after filtering for more than 400 proteins. However, for larger tumor cells, we identified up to 1,500 proteins per single shape (Fig. 5b). cnDVP achieved the highest proteome coverage in both tumor and immune niches, with an average 26% increase in protein identifications relative to DVP and the largest number of uniquely detected proteins slightly enriched for membrane proteins (Fig. 5b,c, Extended Data Fig. 5b,c and Supplementary Table 5). However, the majority of proteins were consistently detected across approaches, and tissue- and niche-specific quantitative signatures were highly correlated (Fig. 5d,e and Extended Data Fig. 5d–f). These results support cnDVP as an efficient strategy that substantially increases throughput while largely preserving cell type specificity and spatial information.

Conceptually, cnDVP expands the DVP method family implemented in openDVP, enabling users to select among complementary profiling strategies based on tissue architecture, biological question and experimental constraints (Fig. 5f).

### cnDVP resolves spatial tumor heterogeneity and relapse-associated molecular programs

**Fig. 6:**
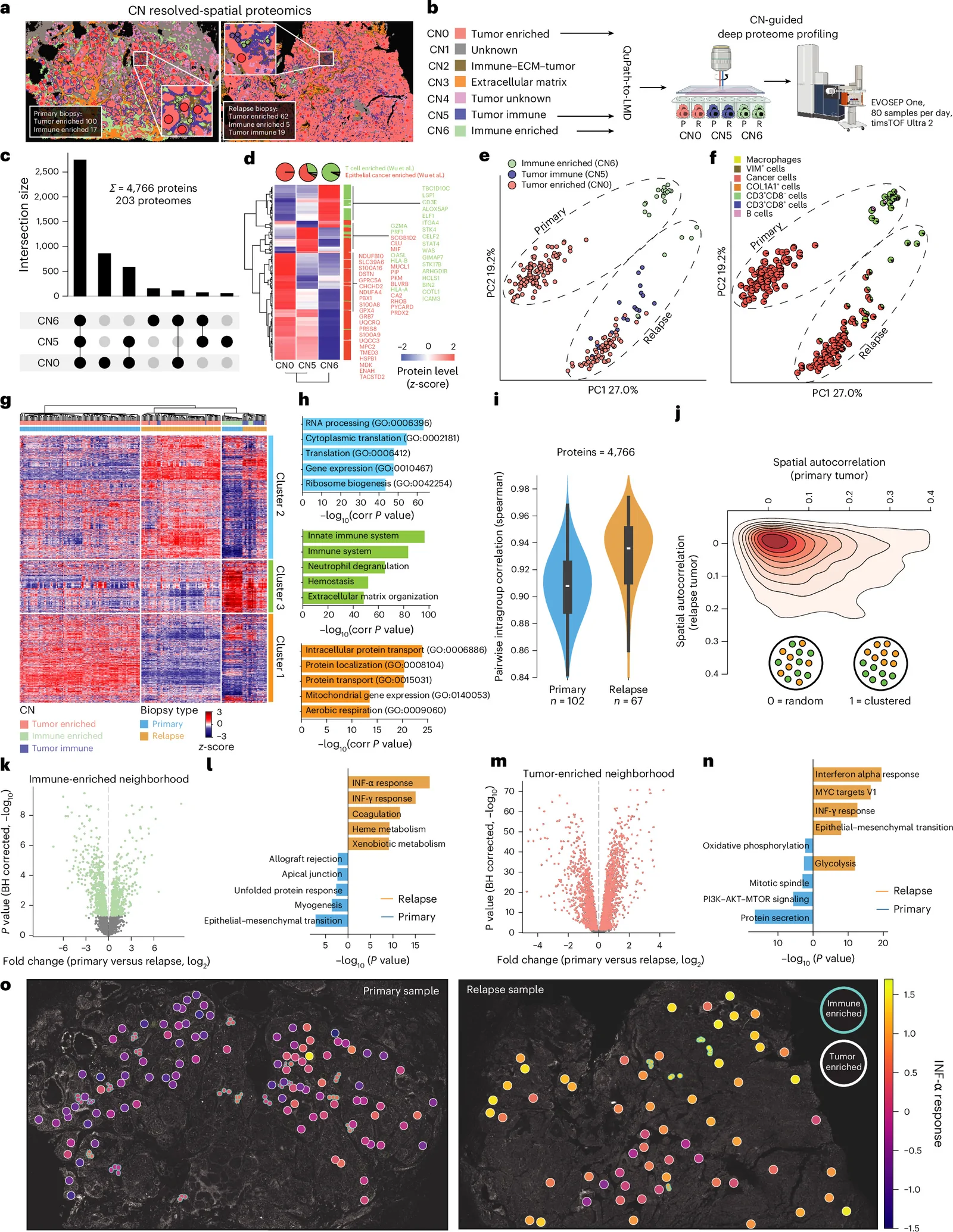
Spatial proteomic analysis of TNBC samples comparing primary and recurrent disease. **a**, Whole-slide overlay of CN annotations with contours for LMD-guided proteomics. Sample collection summaries are shown in the boxes. **b**, Three selected CNs for proteomics: tumor enriched, immune enriched and tumor immune. Regions were collected using LMD and analyzed using a rapid (80 samples per day) LC–MS strategy. **c**, An UpSet plot showing protein identification overlap across CNs. **d**, A heat map showing signature markers from a recent RNA-seq study^56^. Protein levels were *z*-scored, averaged per CN and subjected to unsupervised hierarchical clustering (method: ‘ward’, distance metric: ‘euclidean’). **e**, PCA of all samples, colored by CN. **f**, PCA embedding with samples as pie charts reflecting cell phenotype proportions derived from image analysis. Note that both PCA plots show clustering by sample type (primary versus relapse), followed by CN. **g**, A heat map displaying 4,570 proteins across 203 samples, clustered hierarchically (method: ‘ward’, distance metric: ‘euclidean’). Proteins filtered for 70% valid values in any CN group. **h**, Protein clusters analyzed using EnrichR pathway enrichment using all identified proteins as background, with *P* values calculated using one-sided Fisher’s exact test and FDR adjusted (BH). **i**, Violin plots showing pairwise intragroup correlations, highlighting greater proteomic variability in the primary tumor. *P* value <0.001, Mann–Whitney two-sided test. The central lines indicate the median, the boxes represent the IQR and the whiskers extend to 1.5× IQR of the box. **j**, A density plot of Moran’s *I* spatial autocorrelation for proteins across samples, showing greater spatial variability in the primary tissue. *P* value < 0.001, Mann–Whitney two-sided test. **k**, A volcano plot showing regulated proteins between immune-enriched CNs of primary and relapse tumors, with an FDR cutoff of 5%. **l**, A bar plot showing enriched MsigDB hallmarks between primary and relapse immune-enriched samples. **m**, A volcano plot showing regulated proteins between tumor-enriched CNs of primary and relapse samples. For both volcano plots in **k** and **m**, the *P* values were calculated using a two-sided *t*-test and FDR adjusted (BH, 5%). **n**, A bar plot showing enriched MsigDB hallmarks between primary and relapse tumor-enriched samples. For both **l** and **n** analyses, the *P* values were calculated using a one-sided Fisher’s exact test and FDR adjusted (BH). **o**, Overlay of the INF-α response pathway (Hallmarks) showing *z*-scored pathway levels of tumor- and immune-enriched samples. Note that the relapse sample featured consistently higher IFN levels across tissue. Large circles represent tumor-specific CNs (white outline) and small circles immune-specific CNs (cyan outline). Illustrations in **b** created in BioRender; Coscia, F. https://biorender.com/89ddc1q (2026). corr, corrected; ECM, extracellular matrix; P, primary; R, relapse.

**Extended Data Fig. 6:**
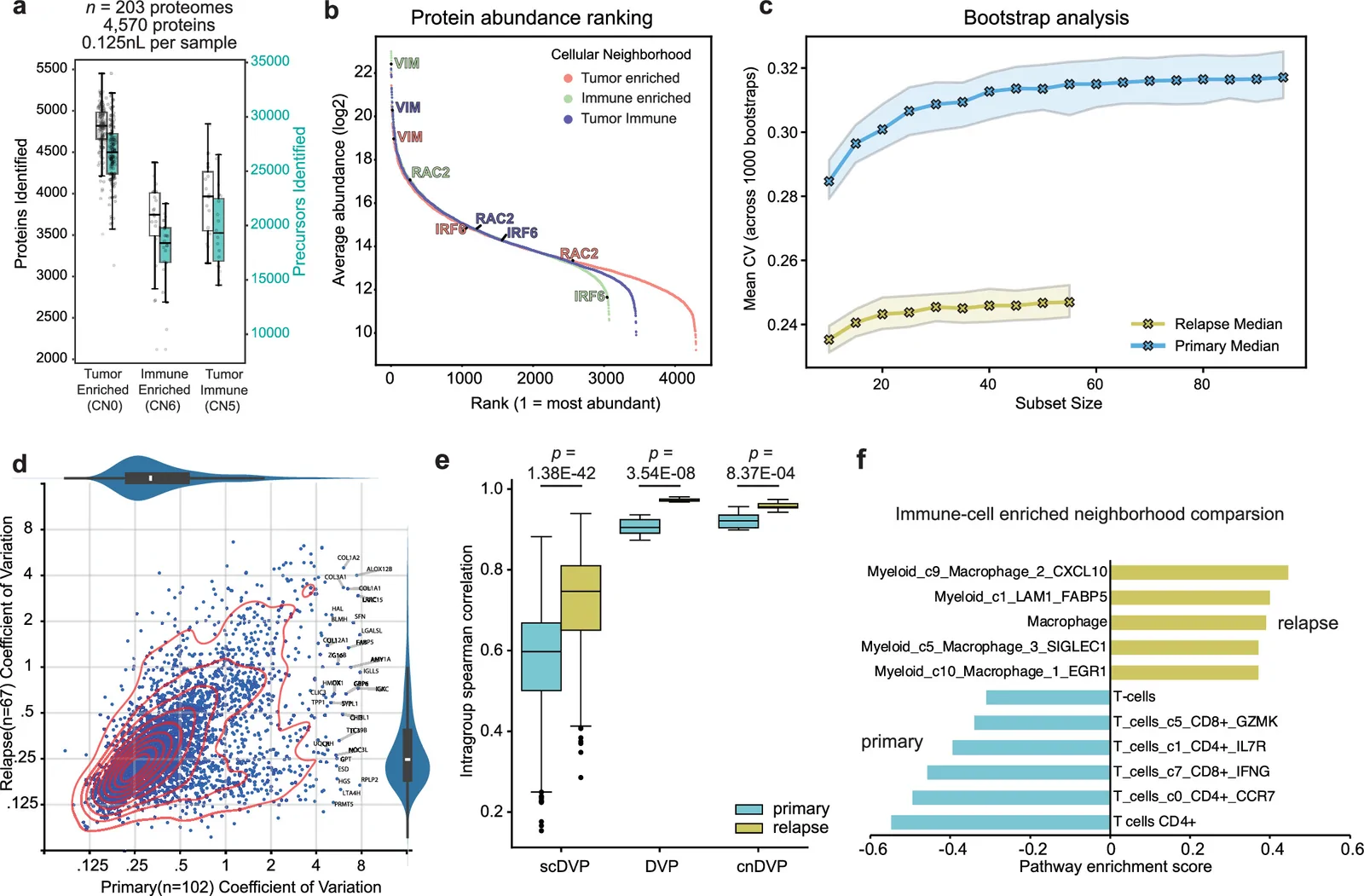
Neighborhood-guided DVP reveals proteomic differences between primary and recurrent TNBC. (**a**) Boxplot showing proteins and precursors identified per group of cellular neighborhoods. (**b**) Rankplot showing the dynamic range of protein abundance for each neighborhood. Vimentin (VIM) and RAC2 show the highest abundance in the immune-enriched samples, and IRF6 in tumor-enriched samples. (**c**) Line plot showing the spread of the mean CV of 1000 bootstrap simulations, repeated across different subset sizes for both samples. Error bars represent 99% confidence interval. Note, plateauing was observed around 30-40 samples. (**d**) Scatterplot of intra-tissue protein level variability (CV) comparing the primary and relapse sample. Only samples of the tumor-specific CN0 were used analysis. Note, the primary tumor showed overall higher proteome variability. (**e**) Boxplots showing intragroup spearman correlation between cancer-only samples for the three DVP approaches scDVP (n = 35,25), DVP (n = 5,5), and cnDVP (n = 4,5); respectively for primary and relapse samples. Protein groups are shown with at least 70% valid values for each sample group (primary or relapse) and approach (DVP, scDVP and cnDVP). P-values were calculated with a two-sided independent t-test. Data available in Suppl. Table 8. (**f**) Barplot of enriched cell type signatures between the primary and relapse sample. Only samples of the immune enriched CN6 were used for analysis. Breast cancer-specific cell type signatures were obtained from publicly available scRNAseq data described in Wu et al. 56. Note, the primary sample showed higher T cell signatures (for example, CD4 + , CD8 + ), whereas the relapsed immune niche featured higher myeloid/macrophage scores. For boxplots in (**a**), (**d**), and (**e**) the central lines indicate the median, the box the interquartile range, whiskers extend to (1.5*IQR) of the box.

To investigate how CN-resolved proteomes reflect tumor progression and treatment resistance, we performed CN-guided LMD sampling for exploratory proteomics (Fig. 6a), focusing on tumor-specific (CN0), tumor-immune interface (CN5) and immune-enriched (CN6) regions. A total of 210 ROIs were distributed across tissue sections to capture spatial heterogeneity (Fig. 6b) and processed within 1 week using an advanced throughput (80 samples per day) DIA-based workflow. After QC filtering, 203 high-quality proteomes remained with 4,500–5,000 proteins in tumor-specific CNs and 3,500–4,000 proteins in immune-enriched niches (Fig. 6c, Extended Data Fig. 6a,b and Supplementary Table 7). Proteomes exhibited strong CN specificity, with shared core proteins and niche-restricted signatures (Fig. 6c). Integration with SpatialData and public single-cell RNA sequencing (RNA-seq) datasets^56^ confirmed epithelial tumor signatures in CN0 and immune programs in CN6 (Fig. 6d). PCA separated proteomes by tissue origin and spatial niche (Fig. 6e,f), and unsupervised clustering identified distinct proteomic programs associated with primary versus relapsed tumor samples (Fig. 6g,h). Tumor-specific CNs showed significantly higher proteomic heterogeneity in the primary sample than in the relapse, as reflected by lower global correlation, increased protein variability and stronger spatial autocorrelation (Fig. 6i,j and Extended Data Fig. 6c–e). This observation also transcended to single tumor shapes collected through DVP and scDVP (Extended Data Fig. 6e and Supplementary Table 8), possibly reflecting reduced intratumoral diversity through clonal selection following chemotherapy^57,58^.

Differential abundance analysis revealed pronounced niche-specific alterations in the relapse sample. Immune niches exhibited upregulation of interferon (INF)-γ signaling, complement, and heme metabolism pathways (Fig. 6k,l), alongside enrichment of myeloid/macrophage programs (Extended Data Fig. 6f). Tumor niches displayed a metabolic shift toward glycolysis and activation of EMT, MYC and INF signaling pathways (Fig. 6m,n), hallmark features of chemoresistance and progressive disease^59,60^. Spatial mapping revealed widespread INF-high tumor phenotypes in the relapse but not in the primary tumor (Fig. 6o), consistent with molecular features of inflammatory TNBC and tumor-immune evasion^61,62^.

Together, these results demonstrate how openDVP integrates whole-slide imaging, CN analysis and exploratory proteomics to resolve spatial tumor heterogeneity and uncover potential drivers of disease progression.

## Discussion

Here, we introduce openDVP as an open-source Python-based framework to make DVP broadly accessible, interoperable and community driven. Built around established open-source tools and the scverse ecosystem, openDVP connects image processing, cell phenotyping, spatial analysis, LMD and MS-based proteomics within a modular workflow. Its use of standardized data formats facilitates the integration of existing and newly developed tools and supports the principles of findable, accessible, interoperable and reusable data. Rather than defining a single implementation of DVP, openDVP provides a flexible framework that can evolve with advances in imaging, computational analysis and ultrasensitive proteomics.

A central concept of openDVP is that it does not require the same level of image analysis or spatial resolution for every biological question. For example, the flashDVP workflow follows a simplified annotate–collect–measure strategy and provides a low-barrier entry point for spatial proteomics, particularly when tissue compartments or morphological structures can be identified directly from microscopy. The applications presented here illustrate how such image-guided proteomic sampling can complement other spatial omics modalities and extend molecular characterization to cellular structures that are difficult to resolve by segmentation-based approaches. More broadly, flashDVP illustrates how direct image annotation can connect histological or spatial phenotypes with exploratory proteomic measurements without requiring a complete single-cell image-analysis workflow.

At the other end of this spectrum, the extended openDVP workflow supports large-scale image processing, single-cell phenotyping and spatial tissue analysis. This enables proteomic sampling strategies to move beyond predefined cell types toward spatially organized multicellular structures. cnDVP represents one such strategy. By collecting and profiling spatially coherent groups of cells rather than individual segmented contours, cnDVP substantially reduces LMD complexity while maintaining spatial and phenotypic information. In our benchmark, this strategy also increased proteome coverage compared with cell pooling (DVP), potentially because larger continuous contours improve tissue recovery and facilitate more reliably visual QC during collection. More broadly, cnDVP expands the DVP method family by providing an intermediate scale between DVP and scDVP. The appropriate sampling strategy can therefore be selected according to the biological question, tissue architecture, required spatial resolution and experimental throughput. cnDVP comes with trade-offs and generally provides lower biological resolution than DVP or scDVP. In its current implementation, it may be most suitable for homotypic or spatially coherent niches. DVP and scDVP remain indispensable when rare phenotypes, cellular heterogeneity within a niche or direct cell-to-cell variation are the primary biological questions^44,63,64^. More generally, the performance of all openDVP workflows depends on tissue quality, staining fidelity, imaging performance, segmentation or annotation accuracy, and LMD efficiency, each of which can introduce technical variability. The biological information available for spatial sampling is determined by the imaging modality and marker panel used to define cells and tissue structures. OpenDVP should therefore not be viewed as a fixed workflow but as a modular framework whose optimal implementation depends on the biological question and experimental context.

Looking ahead, openDVP provides a foundation for further methodological advances in spatial proteomics. Combined with higher-plex imaging^65^, finer definition of cell states and tissue architecture could improve the selection of biologically relevant neighborhoods for downstream proteomic analysis. In parallel, pathology foundation models can further scale the image analysis component of openDVP by enabling more generalizable feature extraction, automated cell classification, and transfer learning across tissues and staining conditions. At the same time, continued improvements in mass spectrometric sensitivity and throughput should enable increasingly smaller and more numerous spatial samples to be analyzed.

Taken together, openDVP provides an open and extensible framework for spatial discovery proteomics across multiple biological scales. By combining complementary sampling strategies with interoperable image and proteomic data structures, it lowers the technical barriers to DVP while preserving the flexibility required for advanced spatial analyses. Community-driven development of individual modules, together with continued advances in imaging, computational pathology and ultrasensitive MS, should further broaden the range of biological questions that can be addressed by spatial proteomics.

## Methods

We structured the methods by analytical process to clarify workflow differences among the three exemplary applications. Detailed information on data sources, image preprocessing, proteomic analysis and all other relevant settings are provided under dedicated subsections.

### Sample collection and patient cohort

#### Placenta samples

Placental tissue was collected from electively terminated pregnancies (gestational age 7–11 weeks) with informed consent. Exclusion criteria were maternal age under 18 years, body mass index >25 kg m^−2^ and maternal pathologies. Ethical approval was obtained from Medical University Graz Ethics Committee (31-019 ex18/19). After surgical extraction, tissue was stored at 4 °C in culture medium (DMEM/F12 1:1, 1 g dl^−1^ glucose) and processed within 4 h. Villous tissue was rinsed in cold (4 °C) 0.9% NaCl solution to remove blood, fixed with 10% formalin, paraffin-embedded (FFPE), and dehydrated per standard protocols.

#### Lung cancer sample

We analyzed the primary tumor of a patient with non-small-cell lung cancer. The patient, a 63-year-old woman with a 40 pack-year smoking history and no physical limitations (Eastern Cooperative Oncology Group performance status 0), was diagnosed in March 2020 with a metabolically active tumor at the apex of the right upper lobe on positron emission tomography imaging. A transbronchial lung biopsy confirmed a TTF1-positive LUAD with acinar growth pattern. The patient underwent right upper lobectomy 2 months after diagnosis. Gross examination was performed according to standardized protocols. The specimen was fixed in 10% buffered formalin and paraffin-embedded (FFPE). Histological examination, including diagnosis, tumor grading, pTNM classification, angioinvasion, lymphatic invasion and tumor stage, was performed according to the 8th edition of the TNM classification (American Joint Committee on Cancer). Following diagnosis, the FFPE tissue block was stored at room temperature (RT) in the archive of the Institute of Pathology at the Charité University Hospital, Campus Mitte. The study was performed according to the ethical principles for medical research of the Declaration of Helsinki, and approved by the Ethics Committee of the Charité University Medical Department in Berlin (EA4/243/21).

A comprehensive 3D ST analysis of this tumor has been described recently based on 34 5-μm-thick consecutive sections (sections 4, 10, 16, 22, 28 and 34 in ref. ^37^) and a section-to-section distance of 30 μm. From the same tumor block, we profiled section 36 using proteomics after image registration in QuPath.

#### TNBC samples

Archival leftover diagnostic tissue from the tumor resection specimen and a specimen obtained 2 years later after relapse was selected from the archives of the Institute of Pathology, Charité – Universitätsmedizin Berlin. At the time of diagnosis and primary resection, the patient had carcinoma in situ (DCIS), 38 mm in diameter, with multifocal stromal invasion. The multifocal invasive tumor had a total diameter of 9 mm, lacked expression of estrogen and progesterone receptors, and had a HER2 score of 1+, considered equivalent to a lack of HER2 gene amplification. The diagnosis was multifocal invasive TNBC (stage pT1b (m)). In 2022, the patient experienced a relapse with a diameter of 34 mm and ulceration of the skin (stage yrpT4b). The patient provided written consent for the use of leftover diagnostic material for research purposes. The project was approved by the Institutional Review Board of Charité (‘Ethics Committee’; project no. EA1/253/19).

#### Membrane slide preparation

Before tissue mounting, slides were washed with 70% EtOH for 5 min, rinsed in ddH_2_O and incubated with poly-l-lysine at 0.01% (w/v) (Sigma-Aldrich, cat. no. P1524) for 10 min to optimize tissue adherence. The slides were then dried overnight at RT. All samples were sectioned at a thickness of 5 µm and mounted on metal frame PPS membrane slides (Leica, cat. no. 11600294), followed by overnight drying at 37 °C. Immediately before deparaffinization, the slides were incubated at 60 °C for 30 min.

### Cyclic and noncyclic IF staining and imaging

#### Placenta tissue

Placenta tissue sections were deparaffinized and subjected to antigen retrieval in Tris/EDTA (pH 9) at 93 °C for 20 min. After cooling (20 min), slides were placed in warm distilled water (5 min) and cooled again (5 min). Sections were washed with PBST (PBS + 0.1% Tween-20) and blocked with Ultra V Block (20 min at RT). For triple staining (CD163, CD31 and E-cadherin), primary antibodies (Extended Data Table 1) were diluted in PBST + 1% normal goat serum and incubated overnight at 4 °C. After washing, secondary antibodies in PBST + 1% normal goat serum (mouse IgG-AF647 and rabbit IgG-AF488; Extended Data Table 1) were incubated for 1 h at RT in the dark. Slides were washed and mounted with SlowFade Diamond containing 4′,6-diamidino-2-phenylindole (DAPI). Rabbit immunoglobulin and mouse IgG controls showed no stains. Imaging was performed using Zeiss Axioscan 7.

#### Lung and breast cancer tissue

Breast cancer and lung cancer tissue sections were deparaffinized and rehydrated (2× 5 min in Neo-Clear, 2× 2 min in 99% EtOH, 1× 2 min in 80% EtOH, 1× 2 min in 70% EtOH, and 3× 1 min in 1× PBS). For decrosslinking, heat-mediated antigen retrieval was performed in pH 9 Tris buffer (1:50 in ddH_2_O; Dako, cat. no. K800421-2) in a steamer for 30 min. Sections were washed in 1× PBS and prequenched in 4.5% H_2_O_2_ (in 25 mM NaOH in PBS) for 2× 30 min. Cover glasses were mounted with 10% glycerol (in PBS), and the first image acquisition for background subtraction was performed using the Zeiss Axioscan 7 Slidescanner with a 20×/0.5 NA objective at 2 × 2 binning. Slides were soaked in PBS for cover glass detachment for 5 min and then washed 3× 5 min in PBS to remove residual glycerol. Tissues were then blocked for 30 min in 3% BSA (in PBS; Serva cat. no. 11948.01) at RT, followed by antibody staining in a humid chamber at 4 °C overnight. Antibodies were all diluted 1:50 in the blocking buffer and are listed in Extended Data Table 1. The next day, sections were washed in PBS, mounted and imaged with similar settings, while exposure times were adjusted to the fluorescent signal. For cyclic (multiplex) IF of breast cancer samples, cover glasses were removed as before, sections were washed and the fluorescent signal was bleached as previously described for 30 min. Sections were washed 3× 5 min in PBS, and the second cycle of antibody staining was performed. Afterward, sections were incubated for 3 min in hematoxylin, 10 min in tap water, dipped twice in ddH_2_O, 30 s in eosin, dipped twice in ddH_2_O, and dehydrated by dipping in increasing concentrations of EtOH (70–99%) for visible staining.

Immunofluorescent staining of lung cancer samples was performed in a single cycle followed by visible staining, as described above.

### Image analysis and contour export for LMD

#### Lung cancer

Immunofluorescent and H&E staining were overlayed using the Warpy extension (version 0.2.6) in Qupath (version 0.4). ROI selection was based on panCK, VIM and E-cadherin staining. Annotations were exported as geoJSON files and converted to the LMD-compatible format (.xml) using the QuPath-to-LMD web application.

#### TNBC

TNBC whole-slide images were processed with MCMICRO: tiles were illumination-corrected with BASIC version 1.1.1, then stitched and registered with ASHLAR version 1.17.1. Channels were background-subtracted from autofluorescence images using Backsub version 0.4.1. The image stack was processed using a modified SOPA snakemake pipeline. The nuclear signal from the first cycle was segmented using cellpose v2’s ‘nuclei’ model, with the following parameters: diameter 25, flow_threshold 0.8, cellprob_threshold –8, min_area 250, clip_limit 0.2 and gaussian_sigma 1. For WSI, we used SOPA’s patchify function to tile images into 5,000-pixel tall/wide squares with 100-pixel overlap to distribute segmentation tasks across HPC (high-performance computing). Segmentation masks were expanded by five pixels and used to quantify mean, standard deviation and three quantiles (25%, 50% and 75%) of marker signal per cell. For QC, image stacks were scaled to 8-bit and pyramidized with a tile size of 4,096. The following additional filtering steps were applied with visual QC: (1) removal of manually labeled artifact regions, (2) removal of cells with too low/high nuclear signal, (3) removal of cells with too small/large areas and (4) removal of dropout cells where DAPI ratio between first and last cycle was not between 0.15 and 1.05. Cell phenotyping was carried out using the napari-cell-gater (github.com/CosciaLab/napari-cell-gater) to determine marker thresholds and scale marker signals for binomial distribution. The plugin allows the user to load an image, segmentation mask and quantification matrix of any sample(s), and then to recursively determine a threshold for each marker–sample combination. This is done with dynamic feedback, and by choosing any two features (intensity or morphology based) to populate a scatterplot. Lastly, we used scimap’s phenotype function using the generated signature-matrix after marker thresholding to label the cells. Artifacts present in the CD8 channel were manually removed prior to analysis. For CN analysis, AnnData objects were merged and analyzed by scimap’s spatial_lda function with 30 *k*-nearest neighbors and *k* = 7.

### LMD and proteomic sample preparation

#### General procedure

We used the Leica LMD7 system with software version 8.3.0.08259. Tissue was collected using the 20× objective (HC PL FL L 20×/0.40 CORR) for lung cancer and breast cancer samples, and 63× objective (HC PL FLUOTAR L 63×/0.70 CORR PH2) for placenta and scDVP samples. Laser settings were optimized per tissue type and listed below. Contours were collected into low-binding 384-well plates (Eppendorf, 0030129547) or into proteoChip EVO96 (C-PEVO-96-10). To ensure tissue settling at the bottom of the wells, 20 μl of acetonitrile was added, followed by centrifugation for 2 min at 2,000*g* and vacuum-drying for 10 min at 60 °C. Wells were finally inspected in the LMD7 system to confirm collection.

Sample purification was performed using Evotips Pure (Evosep) following the manufacturer’s instructions, and samples were kept with buffer A until LC–MS measurement.

##### First-trimester placenta

Contours were collected by LMD using the 63× magnification objective. About 150 cells were collected per sample, in total about 50,000 µm^2^. Cell lysis was performed with 4 μl of 60 mM TEAB (triethylammonium bicarbonate), followed by brief centrifugation (2,000*g*, 1 min) and heating at 95 °C for 60 min in a Bio-Rad thermal cycler (384-well module, lid at 110 °C). ACN (1 μl; 20% final) was added, and the samples were incubated at 75 °C for 60 min. After cooling, 2 μl LysC (2 ng μl^−1^ in LC–MS grade H_2_O) was added, and digestion was performed at 37 °C for 4 h. Trypsin (2 μL, 2 ng μl^−1^; Promega Trypsin Gold) was added for overnight digestion at 37 °C. Digestion was stopped with 1% TFA (trifluoroacetic acid) (v/v), and samples were vacuum-dried (~60 min, 60 °C) and stored at –20 °C. Before LC–MS, peptides were purified using Evotips Pure, eluted into a 96-well plate, vacuum-dried and resuspended in 4.2 μl MS loading buffer (3% ACN, 0.2% TFA); the plate was vortexed (10 s) and centrifuged (5 min, 2,000*g*), and 4 μl was injected into the EASY-nLC-1200 system.

##### Lung cancer

Contours were collected by LMD using the 20× magnification objective. The total area of all samples ranged between 50,000 µm^2^ and 100,000 µm^2^. Cell lysis and protein digestion was performed as described above for the placenta samples. After trypsin digestion, samples were vacuum-dried (~60 min, 60 °C) and stored at –20 °C. Immediately before measurement, C18-based peptide purification (Evotip Pure) was performed, and samples were maintained in buffer A prior to LC–MS analysis.

##### TNBC

The total area for each sample was 25,000 µm^2^. Protein extraction and digestion used a DDM (n-Dodecyl-β-D-maltoside)-based protocol as described above. A lysis buffer containing 0.025% DDM, 5 mM TCEP, 20 mM CAA (2-chloroacetamide) and 0.1 M TEAB in water was dispensed into wells (4 μl per sample) using a MANTIS liquid dispenser. Plates were sealed with PCR ComfortLid seals and incubated at 95 °C for 60 min. After cooling, 2 μl of LysC (4 ng μl^−1^ in 0.1 M TEAB (pH 8.5) with 30% acetonitrile) was added, and samples were digested for 4 h at 37 °C in a thermal cycler. Subsequently, 3 μl of trypsin (2 ng μl^−1^ in 0.1 M TEAB (pH 8.5) with 10% acetonitrile) was added, and digestion proceeded overnight at 37 °C. Samples were vacuum-dried for peptide purification.

### LC–MS

#### First-trimester placenta

Peptides were analyzed using an EASY-nLC 1200 (Thermo Fisher) coupled to a trapped ion mobility spectrometry quadrupole time-of-flight mass spectrometer (timsTOF SCP, Bruker Daltonik). Samples were loaded onto a 20-cm in-house packed C18 column (75 μm ID, 1.9 μm ReproSilPur C18-AQ silica beads, Dr. Maisch) and separated at 250 nl min^−1^ with a gradient of increasing concentrations of buffer B (0.1% formic acid, 90% ACN (acetonitrile) in LC–MS grade H_2_O) to 60% buffer A (3% ACN, 0.1% formic acid in LC–MS grade H_2_O). The gradient duration was 21 min. MS acquisition was performed in dia-PASEF (data-independent acquisition - parallel accumulation–serial fragmentation) mode using the vendor’s default ‘3 × 8’ method. Eight dia-PASEF scans were conducted, each divided into three ion mobility windows, spanning a mass-to-charge ratio (*m*/*z*) range of 400–1,000 with 25-Th windows, and covering an ion mobility range from 0.64 to 1.37 V s cm^−2^. The mass spectrometer was set to high-sensitivity mode, featuring an accumulation and ramp time of 100 ms, a capillary voltage of 1,750 V and a collision energy that increased linearly from 20 eV at 1/*K*_0_ = 0.6 Vs cm^−2^ to 59 eV at 1/*K*_0_ = 1.6 Vs cm^−2^. The collision energy was adjusted linearly on the basis of ion mobility, starting at 59 eV at 1/*K*_0_ = 1.6 Vs cm^−2^ and decreasing to 20 eV at 1/*K*_0_ = 0.6 Vs cm^−2^.

#### Lung cancer

Lung cancer samples were directly eluted from Evotips using the Evosep One coupled to a trapped ion mobility spectrometry quadrupole time-of-flight mass spectrometer (timsTOF Ultra, Bruker Daltonik). Separation was performed using an Evosep 30SPD gradient with an IonOpticks Aurora Elite column. Samples were measured in dia-PASEF acquisition mode. The MS acquisition settings were the same as described above.

#### TNBC

LC was performed using the Evosep One LC system connected to a trapped ion mobility spectrometer with a quadrupole time-of-flight mass spectrometer (timsTOF Ultra 2, Bruker Daltonik). Separation was performed using an Evosep 80SPD gradient with an IonOpticks Aurora Rapid column. Samples were measured in dia-PASEF acquisition mode in high-sensitivity mode with the MS method details as described above.

### Proteomic raw file and data analysis

#### First-trimester placenta

Raw data were processed in DIA-NN (version 1.8.1) using a predicted human spectral library (UniProt, 2021). Default settings were used with adjustments: mass range 100–1,700 *m*/*z*, precursor charge states 2–4, maximum of two miscleavages, MS1/MS2 mass accuracy 15 ppm, match-between-runs enabled and quantification set to ‘Robust LC’. Protein-level outputs (pg_matrix.tsv and unique_genes_matrix.tsv) were analyzed in Perseus^67^ (version 1.16.0.5). Missing values were imputed from a normal distribution (width of 0.3 and downshift of 1.8) after filtering for ≥70% quantified values per cell type group. Matrices were further analyzed in R (version 4.1.2). To identify phenotype-specific protein markers, highly variable proteins were selected on the basis of ANOVA *F*-values greater than four. Differential abundance was assessed using Welch’s two-sided *t*-test followed by Benjamini–Hochberg (BH) false discovery rate (FDR) correction. Identified marker proteins (FDR <0.05) overlapping with public transcriptomic datasets were visualized using DiVenn^68^. Receptor ligand communication analysis was carried out using CellChat and included receptors and ligands indexed as ‘secreted’, ‘cell-cell contact’ or ‘ECM-receptor’ in their database. We used standard parameters (excluding per-protein smoothing) of 1,000 permutations for the probabilistic inference of each cell pair and transmitter–receiver interaction, considering heterometric structures and interactor mediator proteins.

#### Lung cancer

Raw files were analyzed in DIA-NN (version 1.8.1) using a predicted human spectral library (UniProt, 2021) with small changes to default parameters, in brief, mass range 400–1,000, missing cleavages 2, max variable modifications 1, Ox(M) and Ac(N-term) modifications, precursor charge range 2–4, precursor *m*/*z* range 400–1,000, fragment ion *m*/*z* range 200–1,000, mass accuracy 15, MS1 accuracy 15 and MBR (match-between-runs) active. Raw files were preprocessed in Perseus as mentioned above. Missing values were imputed from a normal distribution (width of 0.3 and downshift of 1.8) after filtering for ≥70% quantified values per cell type group. Downstream analysis was performed in R (version 4.3.2). Differential abundance was assessed using Welch’s two-sided *t*-test followed by BH FDR correction of 5%. For comparisons with RNA levels, the ST dataset previously published in collaboration with Pentimalli et al. ^37^ was used. For analyses in Fig. 3i–k, publicly available data from the LUAD TCGA dataset was used.

#### TNBC

Raw files were analyzed in DIA-NN (version 1.9) using a predicted human spectral library (UniProt, release 2022) with small changes to default parameters. In brief, maximum number of variable modifications 1, mass accuracy 15, MS1 accuracy 15 and MBR active.

Proteomic data analysis was performed in Python version 3.12.8, using AnnData verison 0.11.2 and SpatialData version 0.4.0 as the main data formats. Analysis was performed using opendvp functions with geopandas version 1.0.1 and pandas version 2.2.3 in the background. Plotting was performed using matplotlib version 3.10.0, seaborn version 0.13.2 and pycomplexheatmap. The DIA-NN output was processed and adapted to the AnnData object using the openDVP DIANN_to_adata function, and QC samples were filtered out. Proteins that did not have at least 70% valid values in any CN were filtered out. The remaining missing values were imputed by low-abundance normal distribution per protein (width of 0.3 and downshift of 1.8). Scanpy version 1.11.0 was used to perform PCA dimensionality reduction and plotting. Hierarchical clustering and plotting of the heat map took place with pycomplexheatmap version 1.8.1, using method ‘average’, and metric ‘cityblock’. *t*-Tests and ANOVA were performed using pingouin version 0.5.5. Spearman’s correlation was calculated using scipy version 1.16.

### Declaration of generative AI and AI-assisted technologies in the writing process

During the preparation of this manuscript, the authors used Paperpal and ChatGPT to improve the readability and language of the text. After using these tools, the authors reviewed and edited the content as needed and take full responsibility for the content of the publication.

### Reporting summary

Further information on research design is available in the Nature Portfolio Reporting Summary linked to this article.

## Online content

Any methods, additional references, Nature Portfolio reporting summaries, source data, extended data, supplementary information, acknowledgements, peer review information; details of author contributions and competing interests; and statements of data and code availability are available at https://doi.org/10.1038/s41592-026-03262-z.

## Supplementary information

Reporting Summary

Peer Review File

Supplementary Table 1 Benchmarking of image processing steps.

Supplementary Table 2 Precursor and protein identifications of placental tissue.

Supplementary Table 3 Results from analysis of placental cell type analyses.

Supplementary Table 4 Exploratory and statistical analysis of lung cancer proteomic data.

Supplementary Table 5 Descriptive statistics of TNBC image analysis.

Supplementary Table 6 Precursor and protein identifications of TNBC samples.

Supplementary Table 7 Descriptive and statistical analysis of TNBC spatial proteomics data.

Supplementary Table 8 Intragroup spearman correlation between sample and approaches.

## Figures and Tables

**Extended Data Table 1:**
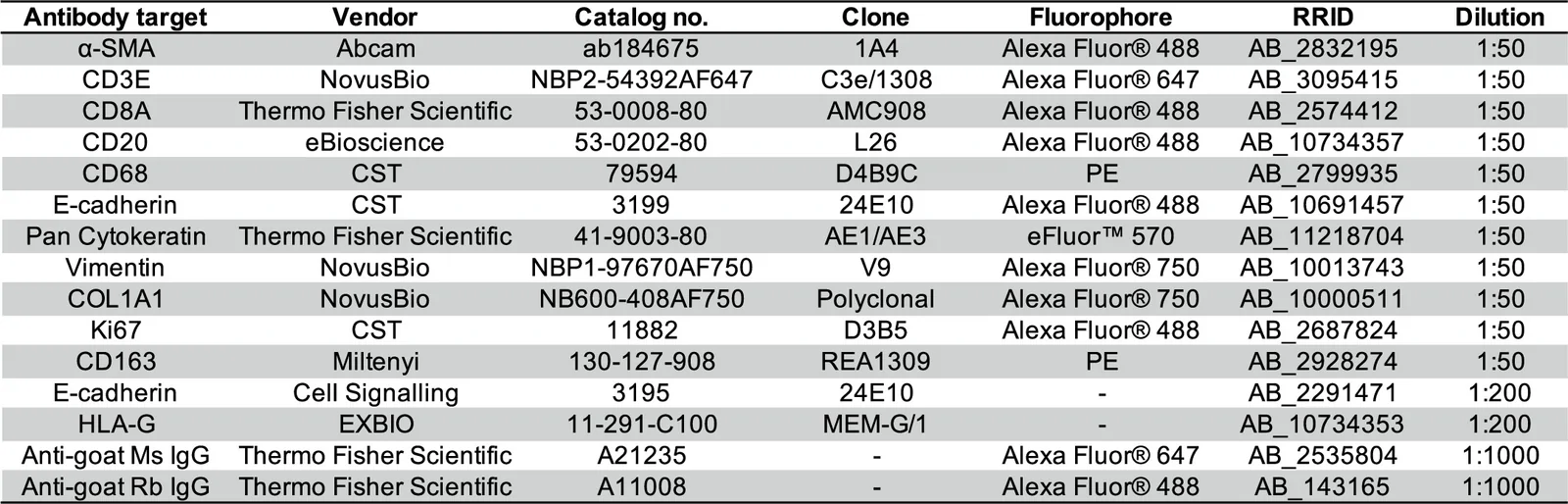
Detailed list of antibodies used in this study

Information includes the target antigen, vendor, catalogue number, clone designation, fluorophore, Research Resource Identifier (RRID), and the specific dilutions used.

## Data Availability

MS proteomics data have been deposited to the ProteomeXchange Consortium via the PRIDE partner repository^69^ with the identifier PXD072882. RNA-seq data were obtained from GEPIA^41^, an interactive web server for analyzing RNA-seq expression data from the TCGA and GTEx projects, using a standard processing pipeline. The LUAD RNA-Seq dataset GEPIA used was based on the UCSC Xena project (http://xena.ucsc.edu). Image data have been deposited to the BioImage Archive repository with the accession no. S-BIAD-2194 (https://www.ebi.ac.uk/biostudies/BioImages/studies/S-BIAD2194).

## Code availability

The source code of openDVP, as well as example tutorials, is available via GitHub at https://github.com/CosciaLab/openDVP. Extensive API documentation of functions is available via GitHub at https://coscialab.github.io/openDVP/index.html. The QuPath-to-LMD web application code is available via GitHub at https://github.com/CosciaLab/Qupath_to_LMD. Lastly, napari-cell-gater is available via GitHub at https://github.com/CosciaLab/napari-cell-gater.

## References

[1] Bressan, D., Battistoni, G. & Hannon, G. J. The dawn of spatial omics. *Science* **381**, 1–35 (2023). 10.1126/science.abq4964 PMC7614974 37535749

[2] Method of the Year 2024: spatial proteomics. *Nat. Methods* **21**, 2195–2196 (2024). 10.1038/s41592-024-02565-3 39643689

[3] de Souza, N., Zhao, S. & Bodenmiller, B. Multiplex protein imaging in tumour biology. *Nat. Rev. Cancer* **24**, 171–191 (2024). 10.1038/s41568-023-00657-4 38316945

[4] Lewis, S. M. et al. Spatial omics and multiplexed imaging to explore cancer biology. *Nat. Methods* **18**, 997–1012 (2021). 10.1038/s41592-021-01203-6 34341583

[5] Aebersold, R. & Mann, M. Mass-spectrometric exploration of proteome structure and function. *Nature* **537**, 347–355 10.1038/nature19949 (2016). 27629641

[6] Horvath, P. & Coscia, F. Spatial proteomics in translational and clinical research. *Mol. Syst. Biol.* **21**, 526–530 10.1038/s44320-025-00101-9 (2025). PMC12130312 40229558

[7] Mund, A., Brunner, A.-D. & Mann, M. Unbiased spatial proteomics with single-cell resolution in tissues. *Mol. Cell* **82**, 2335–2349 (2022). 10.1016/j.molcel.2022.05.022 35714588

[8] Mund, A. et al. Deep visual proteomics defines single-cell identity and heterogeneity. *Nat. Biotechnol.* **40**, 1231–1240 (2022). 10.1038/s41587-022-01302-5 PMC9371970 35590073

[9] Nordmann, T. M. et al. Spatial proteomics identifies JAKi as treatment for a lethal skin disease. *Nature* **635**, 1001–1009 (2024). 10.1038/s41586-024-08061-0 PMC11602713 39415009

[10] Rosenberger, F. A. et al. Deep visual proteomics maps proteotoxicity in a genetic liver disease. *Nature* **642**, 484–491 (2025). 10.1038/s41586-025-08885-4 PMC12158776 40240610

[11] Schapiro, D. et al. MCMICRO: a scalable, modular image-processing pipeline for multiplexed tissue imaging. *Nat. Methods* **19**, 311–315 (2022). 10.1038/s41592-021-01308-y PMC8916956 34824477

[12] Blampey, Q. et al. Sopa: a technology-invariant pipeline for analyses of image-based spatial omics. *Nat. Commun.* **15**, 4981 (2024). 10.1038/s41467-024-48981-z PMC11167053 38862483

[13] Bankhead, P. et al. QuPath: open source software for digital pathology image analysis. *Sci. Rep.* **7**, 1–7 (2017). 10.1038/s41598-017-17204-5 PMC5715110 29203879

[14] Chiu, C.-L., Clack, N. & the napari Community.napari: a Python multi-dimensional image viewer platform for the research community. *Microsc. Microanal.* **28**, 1576–1577 (2022).

[15] Marconato, L. et al. SpatialData: an open and universal data framework for spatial omics. *Nat. Methods* **22**, 58–62 10.1038/s41592-024-02212-x (2025). PMC11725494 38509327

[16] DI Tommaso, P. et al. Nextflow enables reproducible computational workflows. *Nat. Biotechnol.* **35**, 316–319 (2017). 10.1038/nbt.3820 28398311

[17] Köster, J. et al. Sustainable data analysis with Snakemake. *F1000Res* **10**, 33 (2021). 10.12688/f1000research.29032.1 PMC8114187 34035898

[18] Pachitariu, M. & Stringer, C. Cellpose 2.0: how to train your own model. *Nat. Methods* **19**, 1634–1641 (2022). 10.1038/s41592-022-01663-4 PMC9718665 36344832

[19] Greenwald, N. F. et al. Whole-cell segmentation of tissue images with human-level performance using large-scale data annotation and deep learning. *Nat. Biotechnol.* **40**, 555–565 10.1038/s41587-021-01094-0 (2022). PMC9010346 34795433

[20] Goldsborough, T. et al. A novel channel invariant architecture for the segmentation of cells and nuclei in multiplexed images using InstanSeg. Preprint at *bioRxiv* 10.1101/2024.09.04.611150 (2024).

[21] Nirmal, A. J. & Sorger, P. K. SCIMAP: a Python toolkit for integrated spatial analysis of multiplexed imaging data. *J. Open Source Softw.* **9**, 6604 (2024). 10.21105/joss.06604 PMC11173324 38873023

[22] Wolf, F. A., Angerer, P. & Theis, F. J. SCANPY: large-scale single-cell gene expression data analysis. *Genome Biol.* **19**, 1–5 (2018). 10.1186/s13059-017-1382-0 PMC5802054 29409532

[23] Meyer-Bender, M. et al. Spatialproteomics—an interoperable toolbox for analyzing highly multiplexed fluorescence image data. *Nat. Methods* **23**, 1564–1573 (2026). 10.1038/s41592-026-03155-1 PMC13441886 42498882

[24] Regev, A. The human cell atlas—slides. *Elife* **6**, e27041 (2017). 10.7554/eLife.27041 PMC5762154 29206104

[25] Rajewsky, N. et al. LifeTime and improving European healthcare through cell-based interceptive medicine. *Nature* **587**, 377–386 (2020). 10.1038/s41586-020-2715-9 PMC7656507 32894860

[26] Burton, G. J. & Fowden, A. L. The placenta: a multifaceted, transient organ. *Philos. Trans. R. Soc. B* **370**, 20140066 (2015). 10.1098/rstb.2014.0066 PMC4305167 25602070

[27] Bezemer, R. E., Faas, M. M., van Goor, H., Gordijn, S. J. & Prins, J. R. Decidual macrophages and Hofbauer cells in fetal growth restriction. *Front. Immunol.* **15**, 1379537 (2024). 10.3389/fimmu.2024.1379537 PMC11239338 39007150

[28] Makhmut, A. et al. A framework for ultra-low-input spatial tissue proteomics. *Cell Syst.* **14**, 1002–1014 (2023). 10.1016/j.cels.2023.10.003 37909047

[29] Demichev, V., Messner, C. B., Vernardis, S. I., Lilley, K. S. & Ralser, M. DIA-NN: neural networks and interference correction enable deep proteome coverage in high throughput. *Nat. Methods* **17**, 41–44 (2020). 10.1038/s41592-019-0638-x PMC6949130 31768060

[30] Gonzalez, T. L. et al. Sex differences in the late first trimester human placenta transcriptome. *Biol. Sex Differ.* **9**, 4 (2018). 10.1186/s13293-018-0165-y PMC5769539 29335024

[31] Nonn, O. et al. Senescent syncytiotrophoblast secretion during early onset preeclampsia. *Hypertension* **82**, 787–799 (2024). 10.1161/HYPERTENSIONAHA.124.23362 PMC12002046 39440423

[32] Baczyk, D., Drewlo, S. & Kingdom, J. C. P. Emerging role of SUMOylation in placental pathology. *Placenta* **34**, 606–612 (2013). 10.1016/j.placenta.2013.03.012 23628505

[33] Baczyk, D., Audette, M. C., Coyaud, E., Raught, B. & Kingdom, J. C. Spatiotemporal distribution of small ubiquitin-like modifiers during human placental development and in response to oxidative and inflammatory stress. *J. Physiol.* **596**, 1587–1600 (2018). 10.1113/JP275288 PMC5924830 29468681

[34] Ruano, C. S. M. et al. Alternative splicing in normal and pathological human placentas is correlated to genetic variants. *Hum. Genet.* **140**, 827–848 (2021). 10.1007/s00439-020-02248-x PMC8052246 33433680

[35] Turco, M. Y. & Moffett, A. Development of the human placenta. *Development* **146**, dev163428 (2019). 10.1242/dev.163428 31776138

[36] Reese, J. A. et al. Platelet counts during pregnancy. *N. Engl. J. Med.* **379**, 32–43 (2018). 10.1056/NEJMoa1802897 PMC6049077 29972751

[37] Massimo Pentimalli, T. et al. Combining spatial transcriptomics and ECM imaging in 3D for mapping cellular interactions in the tumor microenvironment. *Cell Syst.* **0**, 101261 (2025). 10.1016/j.cels.2025.101261 40220761

[38] David, M. et al. Durable responses to imatinib in patients with PDGFRB fusion gene-positive and BCR-ABL-negative chronic myeloproliferative disorders. *Blood* **109**, 61–64 (2007). 10.1182/blood-2006-05-024828 16960151

[39] Stine, Z. E., Schug, Z. T., Salvino, J. M. & Dang, C. V. Targeting cancer metabolism in the era of precision oncology. *Nat. Rev. Drug Discov.* **21**, 141–162 (2022). 10.1038/s41573-021-00339-6 PMC8641543 34862480

[40] Ligorio, F. et al. Early downmodulation of tumor glycolysis predicts response to fasting-mimicking diet in triple-negative breast cancer patients. *Cell Metab.* **37**, 330–344 (2025). 10.1016/j.cmet.2024.11.004 39694040

[41] Tang, Z. et al. GEPIA: a web server for cancer and normal gene expression profiling and interactive analyses. *Nucleic Acids Res.* **45**, W98–W102 (2017). 10.1093/nar/gkx247 PMC5570223 28407145

[42] Goldman, M. J. et al. Visualizing and interpreting cancer genomics data via the Xena platform. *Nat. Biotechnol.* **38**, 675–678 (2020). 10.1038/s41587-020-0546-8 PMC7386072 32444850

[43] Uhlen, M. et al. A pathology atlas of the human cancer transcriptome. *Science* **357**, eaan2507 (2017). 10.1126/science.aan2507 28818916

[44] Rosenberger, F. A. et al. Spatial single-cell mass spectrometry defines zonation of the hepatocyte proteome. *Nat. Methods* **20**, 1530–1536 (2023). 10.1038/s41592-023-02007-6 PMC10555842 37783884

[45] Liu, Y. et al. Conserved spatial subtypes and cellular neighborhoods of cancer-associated fibroblasts revealed by single-cell spatial multi-omics. *Cancer Cell* **43**, 905–924 (2025). 10.1016/j.ccell.2025.03.004 PMC12074878 40154487

[46] Ma, C. et al. Pan-cancer spatially resolved single-cell analysis reveals the crosstalk between cancer-associated fibroblasts and tumor microenvironment. *Mol. Cancer* **22**, 170 (2023). 10.1186/s12943-023-01876-x PMC10571470 37833788

[47] Barkley, D. et al. Cancer cell states recur across tumor types and form specific interactions with the tumor microenvironment. *Nat. Genet.* **54**, 1192–1201 (2022). 10.1038/s41588-022-01141-9 PMC9886402 35931863

[48] Bianchini, G., De Angelis, C., Licata, L. & Gianni, L. Treatment landscape of triple-negative breast cancer—expanded options, evolving needs. *Nat. Rev. Clin. Oncol.* **19**, 91–113 (2022). 10.1038/s41571-021-00565-2 34754128

[49] Jackson, H. W. et al. The single-cell pathology landscape of breast cancer. *Nature* **578**, 615–620 (2020). 10.1038/s41586-019-1876-x 31959985

[50] Ranek, J. S. et al. The automated computational workflow QUICHE reveals structural definitions of antitumor responses in triple-negative breast cancer. *Nat. Cancer* **7**, 451–468 (2026). 10.1038/s43018-026-01122-5 PMC13148788 41708893

[51] Miroshnychenko, D. et al. Stroma-mediated breast cancer cell proliferation indirectly drives chemoresistance by accelerating tumor recovery between chemotherapy cycles. *Cancer Res.* **83**, 3681–3692 (2023). 10.1158/0008-5472.CAN-23-0398 PMC10646478 37791818

[52] Wang, X. Q. et al. Spatial predictors of immunotherapy response in triple-negative breast cancer. *Nature* **621**, 868–876 (2023). 10.1038/s41586-023-06498-3 PMC10533410 37674077

[53] de Visser, K. E. & Joyce, J. A. The evolving tumor microenvironment: from cancer initiation to metastatic outgrowth. *Cancer Cell* **41**, 374–403 (2023). 10.1016/j.ccell.2023.02.016 36917948

[54] Friedman-DeLuca, M., Karagiannis, G. S., Condeelis, J. S., Oktay, M. H. & Entenberg, D. Macrophages in tumor cell migration and metastasis. *Front. Immunol.* **15**, 1–16 (2024). 10.3389/fimmu.2024.1494462 PMC11563815 39555068

[55] Rosenberger, F. A., Thielert, M. & Mann, M. Making single-cell proteomics biologically relevant. *Nat. Methods* **20**, 320–323 (2023). 10.1038/s41592-023-01771-9 36899157

[56] Wu, S. Z. et al. A single-cell and spatially resolved atlas of human breast cancers. *Nat. Genet.* **53**, 1334–1347 (2021). 10.1038/s41588-021-00911-1 PMC9044823 34493872

[57] Lomakin, A. et al. Spatial genomics maps the structure, nature and evolution of cancer clones. *Nature* **611**, 594–602 (2022). 10.1038/s41586-022-05425-2 PMC9668746 36352222

[58] Casasent, A. K. et al. Multiclonal invasion in breast tumors identified by topographic single cell sequencing article multiclonal invasion in breast tumors identified by topographic single cell sequencing. *Cell* **172**, 205–210 (2018). 10.1016/j.cell.2017.12.007 PMC5766405 29307488

[59] Pavlova, N. N. & Thompson, C. B. The emerging hallmarks of cancer metabolism. *Cell Metab.* **23**, 27–47 10.1016/j.cmet.2015.12.006 (2016). PMC4715268 26771115

[60] Hanahan, D. & Weinberg, R. A. Hallmarks of cancer: the next generation. *Cell* **144**, 646–674 10.1016/j.cell.2011.02.013 (2011). 21376230

[61] Wang, X. et al. Genomic and transcriptomic analyses identify distinctive features of triple-negative inflammatory breast cancer. *npj Precis. Oncol.* **8**, 1–14 (2024). 10.1038/s41698-024-00729-0 PMC11574056 39558017

[62] Benci, J. L. et al. Tumor interferon signaling regulates a multigenic resistance program to immune checkpoint blockade. *Cell* **167**, 1540 (2016). 10.1016/j.cell.2016.11.022 PMC5385895 27912061

[63] Post, F. et al. Deep visual proteomics reveals an in vivo-like phenotype of orthotopically transplanted human colon organoids. *Cell Syst.* **0**, 101396 (2025). 10.1016/j.cels.2025.101396 40934911

[64] Mardamshina, M. et al. Multiplexed deep visual proteomics resolves spatial heterogeneity and rare endocrine states in human pancreatic islets. *Nat. Commun*. 10.1038/s41467-026-77890-6 (2026). PMC13710848 42934244

[65] Zheng, X., Mund, A. & Mann, M. Deciphering functional tumor-immune crosstalk through highly multiplexed imaging and deep visual proteomics. *Mol. Cell* **85**, 1008–1023 (2024). 10.1016/j.molcel.2024.12.023 39814024

[66] Jin, S. et al. Inference and analysis of cell-cell communication using CellChat. *Nat. Commun.* **12**, 1–20 (2021). 10.1038/s41467-021-21246-9 PMC7889871 33597522

[67] Tyanova, S. et al. The Perseus computational platform for comprehensive analysis of (prote)omics data. *Nat. Methods* **13**, 731–740 10.1038/nmeth.3901 (2016). 27348712

[68] Sun, L. et al. Divenn: an interactive and integrated web-based visualization tool for comparing gene lists. *Front. Genet.* **10**, 421 (2019). 10.3389/fgene.2019.00421 PMC6509638 31130993

[69] Vizcaíno, J. A. et al. 2016 update of the PRIDE database and its related tools. *Nucleic Acids Res.* **44**, D447–D456 (2016). 10.1093/nar/gkv1145 PMC4702828 26527722

