# Supplementary material for: OpenDVP: an experimental and computational framework for community-empowered deep visual proteomics
Source: Nat Methods. 2026 Oct 22;23(11):2250–62. doi: 10.1038/s41592-026-03262-z (PMC13736673; doi:10.1038/s41592-026-03262-z)
Supplement: Supplementary file 1 [file 41592_2026_3262_MOESM1_ESM.pdf]

nature portfolio

Corresponding author(s): Fabian Coscia

Last updated by author(s): Apr 15, 2026

Reporting Summary

Nature Portfolio wishes to improve the reproducibility of the work that we publish. This form provides structure for consistency and transparency in reporting. For further information on Nature Portfolio policies, see our Editorial Policies and the Editorial Policy Checklist.

Statistics

For all statistical analyses, confirm that the following items are present in the figure legend, table legend, main text, or Methods section.

n/a	Confirmed	
☐	☒	The exact sample size (*n*) for each experimental group/condition, given as a discrete number and unit of measurement
☐	☒	A statement on whether measurements were taken from distinct samples or whether the same sample was measured repeatedly
☐	☒	The statistical test(s) used AND whether they are one- or two-sided<br>*Only common tests should be described solely by name; describe more complex techniques in the Methods section.*
☐	☒	A description of all covariates tested
☐	☒	A description of any assumptions or corrections, such as tests of normality and adjustment for multiple comparisons
☐	☒	A full description of the statistical parameters including central tendency (e.g. means) or other basic estimates (e.g. regression coefficient) AND variation (e.g. standard deviation) or associated estimates of uncertainty (e.g. confidence intervals)
☐	☒	For null hypothesis testing, the test statistic (e.g. *F*, *t*, *r*) with confidence intervals, effect sizes, degrees of freedom and *P* value noted<br>*Give P values as exact values whenever suitable.*
☒	☐	For Bayesian analysis, information on the choice of priors and Markov chain Monte Carlo settings
☒	☐	For hierarchical and complex designs, identification of the appropriate level for tests and full reporting of outcomes
☐	☒	Estimates of effect sizes (e.g. Cohen's *d*, Pearson's *r*), indicating how they were calculated

Our web collection on statistics for biologists contains articles on many of the points above.

Software and code

Policy information about availability of computer code

Data collection	Data collection for imaging was performed with Zeiss Axioscan 7 Slidescanner with a 20x/0.5NA objective at 2 × 2 binning. Data collection for mass-spectrometry based proteomics was performed with Easy-nLC or EvoSep in combination with Bruker's timsTOF mass spectrometers (models: SCP, Ultra, Ultra 2).
Data analysis	Image data analyis was performed with various software solutions. Image processing was performed with a combination of MCMICRO and SOPA pipelines with published and inhouse scripts uploaded in the image data repository. Downstream image analysis was performed with Python (v3.12), opendvp(v0.5.8), scimap (v2.2); Quality control performed with QuPath (v0.5.0), Napari (v0.5), and opendvp (v0.5.8). Downstream proteomic analysis was performed with a combination of Perseus (v1.6.15), and Python (v3.12) with opendvp (v0.5.8) and opendvp (0.7.3). All custom code is available in www.github.com/CosciaLab/opendvp, https://github.com/CosciaLab/Qupath_to_LMD, and https://github.com/CosciaLab/napari-cell-gater.

For manuscripts utilizing custom algorithms or software that are central to the research but not yet described in published literature, software must be made available to editors and reviewers. We strongly encourage code deposition in a community repository (e.g. GitHub). See the Nature Portfolio guidelines for submitting code & software for further information.

Data

Policy information about availability of data

All manuscripts must include a data availability statement. This statement should provide the following information, where applicable:

- Accession codes, unique identifiers, or web links for publicly available datasets
- A description of any restrictions on data availability
- For clinical datasets or third party data, please ensure that the statement adheres to our policy

Mass spectrometry data is deposited in ProteomeXchange Consortium via the PRIDE partner repository, identifier PXD072882.
Imaging data is deposited in the BioImage Archive repository with accession number S-BIAD-2194.

Research involving human participants, their data, or biological material

Policy information about studies with human participants or human data. See also policy information about sex, gender (identity/presentation), and sexual orientation and race, ethnicity and racism.

Reporting on sex and gender	Not applicable due to sex specific tissue types (placenta and breast cancer) and single tissue analyses.
Reporting on race, ethnicity, or other socially relevant groupings	Not applicable
Population characteristics	not applicable
Recruitment	no patient recruitment, only retrospective analyses
Ethics oversight	Provided in the Methods section:<br>Placenta: Ethical approval was obtained from Medical University Graz Ethics Committee (31-019 ex18/19).<br>Lung Cancer: The study was performed according to the ethical principles for medical research of the Declaration of Helsinki, and approval was approved by the Ethics Committee of the Charité University Medical Department in Berlin (EA4/243/21).<br>Breast cancer: The patient provided written consent for the use of leftover diagnostic material for research purposes. The project was approved by the IRB of Charité ("Ethics Committee"), project number EA1/253/19.

Note that full information on the approval of the study protocol must also be provided in the manuscript.

Field-specific reporting

Please select the one below that is the best fit for your research. If you are not sure, read the appropriate sections before making your selection.

☒ Life sciences ☐ Behavioural & social sciences ☐ Ecological, evolutionary & environmental sciences

For a reference copy of the document with all sections, see nature.com/documents/nr-reporting-summary-flat.pdf

Life sciences study design

All studies must disclose on these points even when the disclosure is negative.

Sample size	Sample sizes were determined from previous studies utilizing similar techniques (see for example: Mund & Coscia et al, Nat Biotech, 2022)
Data exclusions	For proteomics datasets, outliers were removed if they resulted in significantly lower (less than 30%) protein identification compared to the majority of samples of the same sample group.
Replication	All biological and technical replicates are reported in the manuscript. All proteomics experiments had at least three biological or intra-patient tissue replicates.
Randomization	Proteomic samples were measured in random order to reduce potential batch effects.
Blinding	No blinding was performed.

Reporting for specific materials, systems and methods

We require information from authors about some types of materials, experimental systems and methods used in many studies. Here, indicate whether each material, system or method listed is relevant to your study. If you are not sure if a list item applies to your research, read the appropriate section before selecting a response.

Materials & experimental systems

n/a	Involved in the study
☐	☒ Antibodies
☒	☐ Eukaryotic cell lines
☒	☐ Palaeontology and archaeology
☐	☒ Animals and other organisms
☐	☒ Clinical data
☒	☐ Dual use research of concern
☒	☐ Plants

Methods

n/a	Involved in the study
☐	☐ ChIP-seq
☐	☐ Flow cytometry
☐	☐ MRI-based neuroimaging

Antibodies

Antibodies used

α-SMA (Abcam, ab184675, clone 1A4, Alexa Fluor® 488, RRID: AB_2832195)
CD3E (NovusBio, NBP2-54392AF647, clone C3e/1308, Alexa Fluor® 647, RRID: AB_3095415)
CD8A (Thermo Fisher Scientific, 53-0008-80, clone AMC908, Alexa Fluor® 488, RRID: AB_2574412)
CD20 (eBioscience, 53-0202-80, clone L26, Alexa Fluor® 488, RRID: AB_10734357)
CD68 (Cell Signaling Technology, 79594, clone D4B9C, PE, RRID: AB_2799935)
E-cadherin (Cell Signaling Technology, 3199, Alexa Fluor® 488, RRID: AB_10691457)
Pan Cytokeratin (Thermo Fisher Scientific, 41-9003-80, clone AE1/AE3, eFluor™ 570, RRID: AB_11218704)
Vimentin (NovusBio, NBP1-97670AF750, clone V9, Alexa Fluor® 750, RRID: AB_10013743)
COL1A1 (NovusBio, NB600-408AF750, polyclonal, Alexa Fluor® 750, RRID: AB_10000511)
Ki67 (Cell Signaling Technology, 11882, clone D3B5, Alexa Fluor® 488, RRID: AB_2687824)
CD163 (Miltenyi, 130-127-908, clone REA1309, PE, RRID: AB_2928274)
E-cadherin (Cell Signaling Technology, 3195, clone 24E10, unlabeled, RRID: AB_2291471)
HLA-G (EXBIO, 11-291-C100, clone MEM-G/1, unlabeled, RRID: AB_10734353)
Anti-goat Ms IgG (Thermo Fisher Scientific, A21235, Alexa Fluor® 647, RRID: AB_2535804)
Anti-goat Rb IgG (Thermo Fisher Scientific, A11008, Alexa Fluor® 488, RRID: AB_143165)

Validation

Validation statements from manufacturer:
- α-SMA (Abcam, ab184675, clone 1A4, Alexa Fluor® 488, RRID: AB_2832195):
Mouse monoclonal antibody [1A4] conjugated to Alexa Fluor® 488 targeting alpha smooth muscle actin. Validated for flow cytometry (intracellular and surface), and immunocytochemistry/immunofluorescence.
- CD3E (NovusBio, NBP2-54392AF647, clone C3e/1308, Alexa Fluor® 647, RRID: AB_3095415):
Recognizes the epsilon chain of CD3 (16–28 kDa), validated in Western blot, flow cytometry (including CyTOF), immunohistochemistry (paraffin), and multiplex assays. Validated for human and mouse.
- CD8A (Thermo Fisher Scientific, 53-0008-80, clone AMC908, Alexa Fluor® 488, RRID: AB_2574412):
Validated for human tissues, demonstrated in immunohistochemistry (paraffin), immunocytochemistry/immunofluorescence, and flow cytometry.
- CD20 (eBioscience, 53-0202-80, clone L26, Alexa Fluor® 488, RRID: AB_10734357):
Validated for human, cynomolgus, and rhesus monkey. Used in immunohistochemistry (paraffin, low/high pH antigen retrieval), and immunofluorescence on Ramos B-cells.
- CD68 (Cell Signaling Technology, 79594, clone D4B9C, PE, RRID: AB_2799935):
Targets endogenous CD68, a lysosomal/macrophage marker (40–50 kDa). Validated for human and monkey flow cytometry, fixed/permeabilized cells. Conjugated to PE and validated for IHC, IF, and live-cell flow cytometry.
- E-cadherin (Cell Signaling Technology, 3199, Alexa Fluor® 488, RRID: AB_10691457):
Monoclonal antibody against human E-cadherin (~120 kDa), raised against synthetic peptide near residue 780. Validated for human and mouse in IHC (1:100–1:400), IF (1:200–1:400), and flow cytometry (1:50).
- Pan Cytokeratin (Thermo Fisher Scientific, 41-9003-80, clone AE1/AE3, eFluor™ 570, RRID: AB_11218704):
Mouse monoclonal AE1/AE3 cocktail recognizing multiple cytokeratins (40–68 kDa) marking epithelial carcinomas. Validated for human, mouse, rat, non-human primates, dog, cat, rabbit, and chicken. Used in ICC, IHC (frozen and paraffin), and flow cytometry.
- Vimentin (NovusBio, NBP1-97670AF750, clone V9, Alexa Fluor® 750, RRID: AB_10013743):
Mouse IgG1 monoclonal against vimentin (~53.6 kDa). Raised against porcine lens vimentin; no off-target binding. Validated for human, rat, bovine, canine, equine, feline, shrew, and others. Applications: flow cytometry (1:25–1:200), ICC/IF (1:10–1:500), IHC (1:25–1:200), Western blot (1:100–1:1000).
- COL1A1 (NovusBio, NB600-408AF750, polyclonal, Alexa Fluor® 750, RRID: AB_10000511):
Rabbit polyclonal against Type I collagen purified from human/bovine placenta. Cross-reacts with mammalian Type I and Type III collagens. Validated for human, mouse, rat, porcine, bovine, rabbit. Applications: WB (1:1000–1:10,000), ELISA, ICC/IF (1:10–1:500), IHC (1:10–1:200), immunoprecipitation.
- Ki67 (Cell Signaling Technology, 11882, clone D3B5, Alexa Fluor® 488, RRID: AB_2687824):
Targets nuclear proliferation marker Ki-67 (~320–345 kDa), raised against human Ki-67 N-terminus. Validated for human, mouse, rat. Applications: IHC (1:100–1:400), IF (1:100–1:200), flow cytometry (1:50).
- CD163 (Miltenyi, 130-127-908, clone REA1309, PE, RRID: AB_2928274):
Monoclonal antibody against human CD163 (hemoglobin scavenger receptor). Validated for flow cytometry in human cells. Supplied in flow cytometry buffer.
- E-cadherin (Cell Signaling Technology, 3195, clone 24E10, unlabeled, RRID: AB_2291471):
Rabbit monoclonal targeting human/mouse E-cadherin (~135 kDa), raised against synthetic peptide near Pro780. Validated in Western blot (1:1000), IHC (1:400), IF (1:1600), flow cytometry (1:100–1:400).
- HLA-G (EXBIO, 11-291-C100, clone MEM-G/1, unlabeled, RRID: AB_10734353):
Monoclonal antibody validated for human samples, used in Western blotting, IHC (paraffin), IF, and flow cytometry.

- Anti-goat Ms IgG (Thermo Fisher Scientific, A21235, Alexa Fluor® 647, RRID: AB_2535804):
Affinity-purified Goat anti-Mouse IgG (H+L) secondary antibody conjugated to Alexa Fluor™ 647, cross-adsorbed against human IgG and serum to reduce cross-reactivity. Validated for IF and IHC. Recommended dilution 2 µg/mL.
- Anti-goat Rb IgG (Thermo Fisher Scientific, A11008, Alexa Fluor® 488, RRID: AB_143165):
Affinity-purified Goat anti-Rabbit IgG (H+L) secondary antibody conjugated to Alexa Fluor™ 488, cross-adsorbed to minimize cross-reactivity with human IgG. Validated for IF and IHC. Recommended dilution 2 µg/mL.

Animals and other research organisms

Policy information about studies involving animals; ARRIVE guidelines recommended for reporting animal research, and Sex and Gender in Research

Laboratory animals | *For laboratory animals, report species, strain and age OR state that the study did not involve laboratory animals.*

Wild animals | *Provide details on animals observed in or captured in the field; report species and age where possible. Describe how animals were caught and transported and what happened to captive animals after the study (if killed, explain why and describe method; if released, say where and when) OR state that the study did not involve wild animals.*

Reporting on sex | *Indicate if findings apply to only one sex; describe whether sex was considered in study design, methods used for assigning sex. Provide data disaggregated for sex where this information has been collected in the source data as appropriate; provide overall numbers in this Reporting Summary. Please state if this information has not been collected. Report sex-based analyses where performed, justify reasons for lack of sex-based analysis.*

Field-collected samples | *For laboratory work with field-collected samples, describe all relevant parameters such as housing, maintenance, temperature, photoperiod and end-of-experiment protocol OR state that the study did not involve samples collected from the field.*

Ethics oversight | *Identify the organization(s) that approved or provided guidance on the study protocol, OR state that no ethical approval or guidance was required and explain why not.*

Note that full information on the approval of the study protocol must also be provided in the manuscript.

Clinical data

Policy information about clinical studies
All manuscripts should comply with the ICMJE guidelines for publication of clinical research and a completed CONSORT checklist must be included with all submissions.

Clinical trial registration | *Provide the trial registration number from ClinicalTrials.gov or an equivalent agency.*

Study protocol | *Note where the full trial protocol can be accessed OR if not available, explain why.*

Data collection | *Describe the settings and locales of data collection, noting the time periods of recruitment and data collection.*

Outcomes | *Describe how you pre-defined primary and secondary outcome measures and how you assessed these measures.*

Plants

Seed stocks | *Report on the source of all seed stocks or other plant material used. If applicable, state the seed stock centre and catalogue number. If plant specimens were collected from the field, describe the collection location, date and sampling procedures.*

Novel plant genotypes | *Describe the methods by which all novel plant genotypes were produced. This includes those generated by transgenic approaches, gene editing, chemical/radiation-based mutagenesis and hybridization. For transgenic lines, describe the transformation method, the number of independent lines analyzed and the generation upon which experiments were performed. For gene-edited lines, describe the editor used, the endogenous sequence targeted for editing, the targeting guide RNA sequence (if applicable) and how the editor was applied.*

Authentication | *Describe any authentication procedures for each seed stock used or novel genotype generated. Describe any experiments used to assess the effect of a mutation and, where applicable, how potential secondary effects (e.g. second site T-DNA insertions, mosiacism, off-target gene editing) were examined.*

ChIP-seq

Data deposition

☐ Confirm that both raw and final processed data have been deposited in a public database such as GEO.

☐ Confirm that you have deposited or provided access to graph files (e.g. BED files) for the called peaks.

Data access links | *For "Initial submission" or "Revised version" documents, provide reviewer access links. For your "Final submission" document,*

Data access links | *provide a link to the deposited data.*

Files in database submission | *Provide a list of all files available in the database submission.*

Genome browser session (e.g. UCSC) | *Provide a link to an anonymized genome browser session for "Initial submission" and "Revised version" documents only, to enable peer review. Write "no longer applicable" for "Final submission" documents.*

Methodology

Replicates	*Describe the experimental replicates, specifying number, type and replicate agreement.*
Sequencing depth	*Describe the sequencing depth for each experiment, providing the total number of reads, uniquely mapped reads, length of reads and whether they were paired- or single-end.*
Antibodies	*Describe the antibodies used for the ChIP-seq experiments; as applicable, provide supplier name, catalog number, clone name, and lot number.*
Peak calling parameters	*Specify the command line program and parameters used for read mapping and peak calling, including the ChIP, control and index files used.*
Data quality	*Describe the methods used to ensure data quality in full detail, including how many peaks are at FDR 5% and above 5-fold enrichment.*
Software	*Describe the software used to collect and analyze the ChIP-seq data. For custom code that has been deposited into a community repository, provide accession details.*

Flow Cytometry

Plots

Confirm that:

- [ ] The axis labels state the marker and fluorochrome used (e.g. CD4-FITC).
- [ ] The axis scales are clearly visible. Include numbers along axes only for bottom left plot of group (a 'group' is an analysis of identical markers).
- [ ] All plots are contour plots with outliers or pseudocolor plots.
- [ ] A numerical value for number of cells or percentage (with statistics) is provided.

Methodology

Sample preparation	*Describe the sample preparation, detailing the biological source of the cells and any tissue processing steps used.*
Instrument	*Identify the instrument used for data collection, specifying make and model number.*
Software	*Describe the software used to collect and analyze the flow cytometry data. For custom code that has been deposited into a community repository, provide accession details.*
Cell population abundance	*Describe the abundance of the relevant cell populations within post-sort fractions, providing details on the purity of the samples and how it was determined.*
Gating strategy	*Describe the gating strategy used for all relevant experiments, specifying the preliminary FSC/SSC gates of the starting cell population, indicating where boundaries between "positive" and "negative" staining cell populations are defined.*

- [ ] Tick this box to confirm that a figure exemplifying the gating strategy is provided in the Supplementary Information.

Magnetic resonance imaging

Experimental design

Design type	*Indicate task or resting state; event-related or block design.*
Design specifications	*Specify the number of blocks, trials or experimental units per session and/or subject, and specify the length of each trial or block (if trials are blocked) and interval between trials.*
Behavioral performance measures	*State number and/or type of variables recorded (e.g. correct button press, response time) and what statistics were used to establish that the subjects were performing the task as expected (e.g. mean, range, and/or standard deviation across subjects).*

Acquisition

Imaging type(s) — *Specify: functional, structural, diffusion, perfusion.*

Field strength — *Specify in Tesla*

Sequence & imaging parameters — *Specify the pulse sequence type (gradient echo, spin echo, etc.), imaging type (EPI, spiral, etc.), field of view, matrix size, slice thickness, orientation and TE/TR/flip angle.*

Area of acquisition — *State whether a whole brain scan was used OR define the area of acquisition, describing how the region was determined.*

Diffusion MRI ☐ Used ☐ Not used

Preprocessing

Preprocessing software — *Provide detail on software version and revision number and on specific parameters (model/functions, brain extraction, segmentation, smoothing kernel size, etc.).*

Normalization — *If data were normalized/standardized, describe the approach(es): specify linear or non-linear and define image types used for transformation OR indicate that data were not normalized and explain rationale for lack of normalization.*

Normalization template — *Describe the template used for normalization/transformation, specifying subject space or group standardized space (e.g. original Talairach, MNI305, ICBM152) OR indicate that the data were not normalized.*

Noise and artifact removal — *Describe your procedure(s) for artifact and structured noise removal, specifying motion parameters, tissue signals and physiological signals (heart rate, respiration).*

Volume censoring — *Define your software and/or method and criteria for volume censoring, and state the extent of such censoring.*

Statistical modeling & inference

Model type and settings — *Specify type (mass univariate, multivariate, RSA, predictive, etc.) and describe essential details of the model at the first and second levels (e.g. fixed, random or mixed effects; drift or auto-correlation).*

Effect(s) tested — *Define precise effect in terms of the task or stimulus conditions instead of psychological concepts and indicate whether ANOVA or factorial designs were used.*

Specify type of analysis: ☐ Whole brain ☐ ROI-based ☐ Both

Statistic type for inference — *Specify voxel-wise or cluster-wise and report all relevant parameters for cluster-wise methods.*

(See Eklund et al. 2016)

Correction — *Describe the type of correction and how it is obtained for multiple comparisons (e.g. FWE, FDR, permutation or Monte Carlo).*

Models & analysis

n/a	Involved in the study
☐	☐ Functional and/or effective connectivity
☐	☐ Graph analysis
☐	☐ Multivariate modeling or predictive analysis

Functional and/or effective connectivity — *Report the measures of dependence used and the model details (e.g. Pearson correlation, partial correlation, mutual information).*

Graph analysis — *Report the dependent variable and connectivity measure, specifying weighted graph or binarized graph, subject- or group-level, and the global and/or node summaries used (e.g. clustering coefficient, efficiency, etc.).*

Multivariate modeling and predictive analysis — *Specify independent variables, features extraction and dimension reduction, model, training and evaluation metrics.*
