# Supplementary material for: OpenDVP: an experimental and computational framework for community-empowered deep visual proteomics
Source: Nat Methods. 2026 Oct 22;23(11):2250–62. doi: 10.1038/s41592-026-03262-z (PMC13736673; doi:10.1038/s41592-026-03262-z)
Supplement: Supplementary file 2 [file 41592_2026_3262_MOESM2_ESM.pdf]

nature portfolio

Peer Review File

OpenDVP: An experimental and computational framework for community-empowered deep visual proteomics

Corresponding Author: Dr Fabian Coscia

This file contains all editorial decision letters in order by version, followed by all author rebuttals in order by version.

Version 0:

Decision Letter:

22nd Sep 2025

Dear Dr Coscia,

Your Article, "OpenDVP: An experimental and computational framework for community-empowered deep visual proteomics", has now been seen by 4 reviewers. As you will see from their comments below, although the reviewers find your work of considerable potential interest, they have raised a number of concerns. We continue to be interested in potentially publishing your manuscript in Nature Methods, but would like to evaluate your response to these concerns before we reach a final decision on publication.

We therefore invite you to revise your manuscript to fully address these concerns especially those about benchmarking and validation.

We are committed to providing a fair and constructive peer-review process. Do not hesitate to contact us if there are specific requests from the reviewers that you believe are technically impossible or unlikely to yield a meaningful outcome.

When revising your paper:

* include a point-by-point response to the reviewers and to any editorial suggestions

* please underline/highlight any additions to the text or areas with other significant changes to facilitate review of the revised manuscript

* address the points listed described below to conform to our open science requirements

* ensure it complies with our general format requirements as set out in our guide to authors at www.nature.com/naturemethods

* resubmit all the necessary files by using the link below to access your home page

Link Redacted

Note: This URL links to your confidential account and associated information about manuscripts you may have submitted, or that you are reviewing for us. If you wish to forward this email to co-authors, please delete this link.

We hope to receive your revised paper within four months. If you are substantially delayed, please let us know. In this event, we will still be happy to reconsider your paper at a later date so long as nothing similar has been accepted for publication at Nature Methods or published elsewhere.

OPEN SCIENCE REQUIREMENTS

REPORTING SUMMARY
When revising your manuscript, please update your reporting summary.

Reporting summary: https://www.nature.com/documents/nr-reporting-summary.zip

If your paper includes custom software, we also ask you to complete a supplemental reporting summary.

Software supplement: https://www.nature.com/documents/nr-software-policy.pdf

Please submit these with your revised manuscript. They will be available to reviewers to aid in their evaluation if the paper is re-reviewed. If you have any questions about the checklist, please see http://www.nature.com/authors/policies/availability.html or contact me.

Please note that these forms are dynamic 'smart pdfs' and must therefore be downloaded and completed in Adobe Reader. We will then flatten them for ease of use by the reviewers. If you would like to reference the guidance text as you complete the template, please access these flattened versions at http://www.nature.com/authors/policies/availability.html.

For any revision that includes light microscopy data, we ask our authors to please include a completed light microscopy reporting table [https://www.nature.com/documents/Light_microscopy_reporting_table.xlsx] to ensure the methods are described thoroughly. The table will be available to reviewers and ultimately published should the manuscript be accepted at the journal.

EXTENDED DATA FIGURES
When re-submitting your manuscript, please ensure that any supplementary figures and tables that are crucial to the manuscript's conclusions are converted into Extended Data figures and tables to increase visibility of these data. Extended Data figures and tables are online-only (present in the online PDF and full-text HTML versions of the paper), peer-reviewed display items that provide essential background to the article but are not included in the main article due to space constraints. A maximum of ten Extended Data display items (figures and tables) is permitted.

DATA AVAILABILITY
We strongly encourage you to deposit all new data associated with the paper in a persistent repository where they can be freely and enduringly accessed. We recommend submitting the data to discipline-specific and community-recognized repositories; a list of repositories is provided here: http://www.nature.com/sdata/policies/repositories

All novel DNA and RNA sequencing data, protein sequences, genetic polymorphisms, linked genotype and phenotype data, gene expression data, macromolecular structures, and proteomics data must be deposited in a publicly accessible database, and accession codes and associated hyperlinks must be provided in the "Data Availability" section.

For papers containing bioimaging data, we strongly recommend depositing the data to Bioimage Archive (https://www.ebi.ac.uk/bioimage-archive/). Associated accession codes and hyperlinks should be provided in the "Data Availability" section.

Refer to our data policies here: https://www.nature.com/nature-research/editorial-policies/reporting-standards#availability-of-data

To further increase transparency, we encourage you to provide, in tabular form, the data underlying the graphical representations used in your figures. This is in addition to our data-deposition policy for specific types of experiments and large datasets. For readers, the source data will be made accessible directly from the figure legend. Spreadsheets can be submitted in .xls, .xlsx or .csv formats. Only one (1) file per figure is permitted: thus if there is a multi-paneled figure the source data for each panel should be clearly labeled in the csv/Excel file; alternately the data for a figure can be included in multiple, clearly labeled sheets in an Excel file. File sizes of up to 30 MB are permitted. When submitting source data files with your manuscript please select the Source Data file type and use the Title field in the File Description tab to indicate which figure the source data pertains to.

Please include a "Data availability" subsection in the Online Methods. This section should inform readers about the availability of the data used to support the conclusions of your study, including accession codes to public repositories, references to source data that may be published alongside the paper, unique identifiers such as URLs to data repository entries, or data set DOIs, and any other statement about data availability. At a minimum, you should include the following statement: "The data that support the findings of this study are available from the corresponding author upon request", describing which data is available upon request and mentioning any restrictions on availability. If DOIs are provided, please include these in the Reference list (authors, title, publisher (repository name), identifier, year). For more guidance on how to write this section please see: http://www.nature.com/authors/policies/data/data-availability-statements-data-citations.pdf

CODE AVAILABILITY
Please include a "Code Availability" subsection in the Online Methods which details how your custom code is made available. Only in rare cases (where code is not central to the main conclusions of the paper) is the statement "available upon request" allowed (and reasons should be specified).

We request that you deposit code in a DOI-minting repository such as Zenodo, Gigantum or Code Ocean and cite the DOI in the Reference list. We also request that you use code versioning and provide a license.

For more information on our code sharing policy and requirements, please see:
https://www.nature.com/nature-research/editorial-policies/reporting-standards#availability-of-computer-code

ORCID
Nature Methods is committed to improving transparency in authorship. As part of our efforts in this direction, we are now requesting that all authors identified as 'corresponding author' on published papers create and link their Open Researcher and Contributor Identifier (ORCID) with their account on the Manuscript Tracking System (MTS), prior to acceptance. This applies to primary research papers only. ORCID helps the scientific community achieve unambiguous attribution of all scholarly contributions. You can create and link your ORCID from the home page of the MTS by clicking on 'Modify my Springer Nature account'. For more information please visit please visit www.springernature.com/orcid.

Please do not hesitate to contact me if you have any questions or would like to discuss these revisions further. We look forward to seeing your revised manuscript and thank you for the opportunity to consider your work.

Best regards,
Madhura

Madhura Mukhopadhyay, PhD
Senior Editor
Nature Methods

Reviewers' Comments:

Reviewer #1 (Remarks to the Author):

This manuscript describes OpenDVP, an open-source framework for deep visual proteomics that integrates image preprocessing, AI-driven segmentation/phenotyping, LMD export, and downstream MS analysis. The idea is timely and potentially valuable to the proteomics community, but the submission currently lacks critical technical detail and quantitative evaluation. Before the work can be considered for publication, the authors should provide stronger evidence of methodological novelty, robustness across imaging and LMD platforms, and quantitative validation of each stage in the image-to-proteomics workflow.

General Concerns
1. Lack of technical details and unclear innovation. The technical description of the OpenDVP platform is overly simplistic. Beyond the integration of existing algorithms, what are the core innovations of OpenDVP for the DVP workflow? The authors should explicitly present them with related data support.
2. Absence of quantitative benchmarking for key algorithms. Several critical algorithms—such as cell segmentation, mask file generation, and real Laser Capture Microdissection (LMD) performance—lack rigorous quantitative comparison against established state-of-the-art methods. This omission makes it difficult to objectively assess their performance and advantages.
3. Disconnected biological applications undermine systematic performance evaluation. The three presented biological applications appear too independent of each other, and their specific role in evaluating the platform's performance is not clearly defined, such as the scanning equipment. We recommend focusing on two applications and structuring the evaluation logically. A direct comparison is essential, particularly to highlight the the performance at each key step. Crucially, this evaluation should include direct, quantitative comparisons with equivalent modules from other modular software platforms to concretely position OpenDVP's performance within the existing landscape.

Technical Concerns
1. Novelty and Differentiation from Existing Tools (e.g., BIAS): The authors was involved in previously developed BIAS (Biology Image Analysis Software). To strengthen the Introduction, a more granular analysis of the limitations of existing software tools, including BIAS, is required. The authors should explicitly clarify the differences and novelty between OpenDVP and BIAS. Additionally, OpenDVP is presented as an integrative framework combining existing tools for DVP workflows; the technical innovations of OpenDVP need to be clearly articulated.
2. Accuracy and Precision of the Cell Segmentation: The authors claimed that "OpenDVP provides a framework to process images into feature tables and analyze matrices to phenotype cells, using open-source tools." (Line 105-106). Cell segmentation, especially for the single cell plasma membrane, is very critical for DVP workflow. Since the author didn't develop their own cell segmentation method, the authors should provide quantitative comparison between existing methods such as CellPose, Mesmer, Cellseg, etc. A clear description how these tools can be efficiently applied through OpenDVP as compared with direct application. The term "deep" in deep visual proteomics refers to both deep learning and high-coverage

proteomic analysis. The authors should highlight the performance of the AI-driven tools they properly evaluated.
Using scimap's spatialLDA algorithm, seven cellular neighborhoods (CNs) are divided according to cell distribution, with each shown as a square. How is the accuracy of this segmentation ensured? We noticed that in Extended data Fig.4e, the algorithm-based cell segmentation does not appear fully accurate at single-cell resolution. For instance, in the CD3E and Vimentin staining panels, the recognized cell contours do not precisely align with the actual staining signals.
Specifically, the authors highlighted that segmenting about a million cells from centimeter-scaled whole slide images in less than 3 hours. The authors need to specify if this refers to brightfield or multi-color images, as segmentation times differ significantly. The stated time should exclude fine-tuning, as initial results are often suboptimal.
3. The gating function needs validation on more complex cell types with advanced metrics: OpenDVP provides interactive annotation and quality control via the napari-cell-gater plugin in Napari. However, the gating is overly simplistic, relying only on area and marker intensity, with limited discrimination power. The authors should provide more details about this critical step for filtering out proper single cells for downstream LCM and demonstrate more challenging examples, such as gating small immune cells in tumor microenvironments.
4. Compatibility with LMD Platforms: The authors mention that this platform supports both LMD7 and MMI systems (Line162-163) which is nice. However, the authors fail to provide related details about how the mask file can be properly import into both systems and match with the real tissue slice. Quantitative data (e.g., on cutting accuracy, precision, and protein yield) obtained using the LMD7 and the MMI system should be provided, especially with single cell resolution. Related practical insight would greatly benefit user implementation the method like QuPath-to-LMD.
5. Reproducibility Issues with the GitHub Repository and Tutorials:
When we test the OpenDVP method, we encountered technical difficulties:
Issue 1: In Tutorial 1, execution halted after the following line, intended to convert a segmentation mask into a GeoDataFrame containing polygons:
gdf = dvp.io.segmask_to_qupath(path_to_segmentation, simplify_value=1, save_as_detection=True)
gdf.head()
The system was unable to locate or invoke any functions from the 'dvp.io' module, and this problem extended to all functions within it. An AttributeError was raised, indicating that the 'opendvp' module lacks an 'io' attribute.
Issue 2: Attempting installation via 'pip install opendvp' in a Python 3.11 environment proved unsuccessful. The process failed during the "getting requirements to build wheel" stage, resulting in a subprocess errors.
Issue 3: Using Python 3.13 allowed for a successful installation of opendvp. However, fundamental operations continued to fail. For instance, in tutorial2.py, the following call:
adata = dvp.io.DIANN_to_adata(# paths and parameters here)

Minor Concerns
1. Figure 4b shows poor image quality. Figure 4c has evident shading correction issues (uneven brightness) and focus problems (e.g., region 4). Under such imaging quality, quantitative assessment of segmentation accuracy is needed for downstream analysis.
2. For the image data collected by OpenDVP, the first step is to convert it into TIFF format (Line 104, Line 125), followed by analysis using flashDVP or AI-driven image analysis. It is necessary to clarify: whether this format conversion process affects image quality; and whether it causes distortion, especially for complex multi-channel fluorescently stained images.
3. The claim that “We annotated 150 cells per replicate for each cell type … to reach an equal tissue amount per sample” (Lines 205-206) is misleading, as flashDVP does not perform image segmentation to obtain true cell counts.
4. In Figure 5o, it’s not clear what do the two differently colored small circles represent.
5. Line 36: scverse → scverse (single-cell universe ecosystem) for reader clarity.
6. Line 142: “Jupiter” should be written as “Jupyter”.
7. Line 344: The authors applied H&E staining of the same slide; how are fluorescent staining images aligned with H&E images?
8. Line 346: “1'005,051” should be written as “1,005,051”.
9. Line 389: “µm2” should be written as “µm²”. Please thoroughly check the entire text.

Reviewer #2 (Remarks to the Author):

The manuscript presents an open-access pipeline for deep visual proteomics (DVP) that integrates spatial proteomics data in a multimodal framework. This enables the mapping of proteomic signatures across regions of interest and cellular neighborhoods. A major strength of this work is its open, Python-based design, which builds on established software tools including MCMICRO, SOPA, QuPath, Napari, and SpatialData and provides an end-to-end framework for proteomics and multimodal analysis. The authors introduce two complementary approaches for image analysis: flashDVP, a segmentation-free method designed for less experienced users and for analyzing syncytial tissue layers, and an advanced AI-based strategy capable of performing cell segmentation, phenotype classification, and neighborhood characterization. Three proof-of-concept case studies are provided to demonstrate the pipeline's applicability, two employing flashDVP and one the AI-driven approach.

The paper presents many exciting avenues that can make DVP a more mainstream application. It is timely and forward-looking, focusing on open source, FAIR principles, and popular tools readily adopted and, importantly, community-maintained. This makes DVP future-proof. The manuscript is well written and the tools are great, yet it is mostly a software-implementation effort and misses somewhat a scientific edge. Please find some specific comments and requests below.

Major comments:
- The workflow moves DVP away from single-cell isolation in the original publication(s) to regions of interest. This is at times

difficult to understand from the text, and I would recommend being clearer about what is based on single-shape isolation and what is ROI-based.
- The ROI-based approach raises the question of whether the presented methods are still an iteration of Deep Visual Proteomics, or rather laser microdissection with smart ROI selection that requires a different term. I find this very confusing as it will dilute the term DVP and make it also confusing for the community. Could the authors comment on this in the discussion? Currently, a technical advantage is presented which is that single shape-based approaches suffer from electrostatic forces impacting gravity-forced collection. I think other work in that field at least does not outline this as a problem.
- The appeal of DVP is the cell-type enrichment, which is lost with an ROI-based approach, irrespective of the certainly smart way of selecting ROIs due to CNs or spatial transcriptomics. Can the authors convincingly show that their new avenue delivers as good biological data as cell-type enrichment? I think this is especially important if the workflow claims to be a universal toolkit for DVP. How does it then actually perform on a single-shape level (besides the manual single-shape selection in the placenta case study)?
- Cellular Neighborhoods are in principle a great idea, but here it lacks benchmarking that this is the smartest way to discover new biology. Can this be formalized and compared against an approach with fewer or more niches?
- The authors described a visual quality control check using the AI-based approach. However, it remains unclear how the accuracy of the ROIs was determined within the model. Did they apply cross-validation or any other systematic method to evaluate the accuracy of ROI classification across phenotypes?
- For FlashDVP, the method uses manual annotation, which makes its scalability to large cohorts either very limited or highly time-consuming. To demonstrate true applicability in large-scale studies, why did the authors not consider developing a deep learning-based strategy (such as pixel-based methods) that can be trained to automatically identify ROIs? By integrating features such as shape, texture, and thresholds, the model could be trained effectively (while remaining independent of single-cell segmentation), and transfer learning could further enhance its applicability in large-scale settings.
- Similarly, the AI-based pipeline also seems to lack a transfer-learning step, limiting its adaptability and use in large-scale cohorts.
- Using the QuPath-to-LMD workflow, ROI coordinates were transferred to the laser microdissection microscope. Please report the spatial accuracy of the contour transfer from image annotation to the laser microdissection at 20× and 63× magnification (i.e., how precisely do the annotated contours align with the actual microscope image), and describe how the QuPath-LMD interface was calibrated to ensure accurate contour transfer.
- Cell size varies substantially based on cell type, cell-cycle stage (G1/S/G2), and pathologic state (e.g., cancer vs. normal), which is reflected in the nucleus-to-cytoplasm (N/C) ratio. Since the proposed study uses segmentation based on nuclear identification, can the authors clarify how the cytoplasmic area was determined, and how the model handles cells with different N/C ratios, for example, epithelioid vs. fusiform cells or lymphocytes versus lung carcinoma cells?
- Between lines 410–421 and in Fig. 5i–j, the authors claim that the DCIS proteome of the primary tumor is more heterogeneous than the relapse sample, citing a lower global proteome correlation (mean Pearson $r = 0.91$ vs. 0.95; Fig. 5i), higher protein-level variability, and higher spatial autocorrelation (Moran's I; Fig. 5j). However, I think the current data do not entirely support the idea of heterogeneity. Both Pearson r values remain high, and the p-value could reflect technical factors (e.g., mass spectrometry or sample-processing variation) rather than true biological differences. Moreover, Moran's I results are reported without p-values, making it difficult to assess statistical robustness. In that regard, single-cell measurements would help get a cleaner read on intra-tumor heterogeneity, especially since the method can potentially be applied for single-cell resolution.
- The biological examples are compelling and well chosen (I particularly like the combination of flashDVP with placenta and the reasoning). However, the examples are much underpowered and lack appropriate controls. This should be included in the discussion as a shortcoming of the examples. The biological interpretation could take up less space in the manuscript, at the benefit of properly benchmarking the workflow parts as described above.

Minor comments:
- In Fig. 4j, please report the statistical differences between the box plots.
- In lines 401–402, the PC1 and PC2 variance values don't match those shown in Fig. 5e–f; please correct them.
- In the lung tumor analysis, the authors report 133 FDA-approved drug targets enriched in the EMT niche. Please clarify the criteria used to compile this set, and whether any drug-enrichment/connectivity analysis was applied?
- Given that N-cadherin is a canonical EMT marker (cadherin switch), please explain why E-cadherin was prioritized.
- In lines 269–270 the authors describe the EMT niche as panCK+/VIM+/E-cadherin-, but Fig. 3c–d and the legend indicate E-cadherin+. Please reconcile this text–figure discrepancy.
- Please describe the staining protocol applied for lung tissue.
- Regarding the cell-neighborhood spatial proteomics analysis, please clarify why tumor–immune ROIs (CN5) were not sampled in the primary biopsy. Also, explain the rationale for the imbalance in tumor-enriched ROIs (sample numbers) between the relapse and primary biopsies.
- The collected samples reported for CN in Fig. 5a (210) does not match the number reported in the text (203); please reconcile this discrepancy.
- Please clarify what the two box plots in Extended Fig. 5a correspond to (primary and relapse biopsy?). This is unclear since tumor–immune regions were not considered for the primary biopsy.
- Please provide the reference for the data shown in Fig. 5k.
- Figure 1 is confusing at times and needs cleaning. The point of panel e is unclear even with the legend.
- The paper introduces a number of new or at least forefront concepts and avenues for DVP, such as spatial transcriptomics on a consecutive section, multiplexed imaging for CN-based ROI identification. This is almost too much for one single paper, but the authors must be congratulated for the excellent job in combining this. However, I feel at times the innovations are not ready to be claimed yet. Particularly, tissue microarrays are mentioned as an innovation, but no data is shown.
- The discussion lacks comparison against other tools in the field, such as BIAS (Cell Signaling Technologies, Hungary) or scPortrait (develop in Matthias Mann's lab). It is also fair to acknowledge other efforts next to this work. Besides this, the references cited are appropriate.

- The Github repository is easy to navigate, and the provided video introduction Qupath-to-LMD is well done.

Reviewer #3 (Remarks to the Author):

I co-reviewed this manuscript with one of the reviewers who provided the listed reports. This is part of the Nature Methods initiative to facilitate training in peer review and to provide appropriate recognition for Early Career Researchers who co-review manuscripts.

Reviewer #4 (Remarks to the Author):

Nimo and colleagues are presenting a manuscript to introduce openDVP, an experimental and computational framework for simplifying and democratizing deep visual proteomics (DVP). Built on existing open-source software for image analysis and data analysis they develop additional packages and interfaces to offer two workflows for user guided and AI guided analysis. They then demonstrate the application of openDVP on three highly relevant use cases.

DVP is a relatively new proteomic strategy co-developed by the authors that combines identification of cells or tissue regions of interest based on multi-parametric IF, IHC or histology images with laser capture microdissection and deep mass spectrometry-based proteome quantification. The technology is extremely powerful and is already showing major breakthroughs yet it is technically challenging and complex to implement. To date it is only being used by a limited number of specialized laboratories and the true transformative potential of the technology will only be reached if it becomes widely accessible and implemented.

This is the gap that Nimo et al address with their contribution. As such the manuscript and openDVP fills and important need, is of interest to a wide audience and has the potential to accelerate implementation and use of DVP enabling many discoveries across biology and biotechnology.

The data and methodology are of high quality and for the most part appropriate statistics has been used (see minor point below). The conclusions drawn from the three exemplary analyses are well reasoned based on the data obtained but would require orthogonal validation which is not provided. However, given that the major focus of the manuscript is the introduction of openDVP this seems acceptable as long as this caveat is clearly pointed out. The manuscript is well written and the code is fully accessible and accompanied by documentation, example datasets and tutorials.

While the manuscript is addressing a critical gap and has the potential to be of wide interest, I feel that critically important information is missing to achieve this goal (and enable proper in-depth review).
- Details on new packages and functions: Very little information is provided about the individual packages and interfaces that have been developed as part of this work. They are accessible on github but without in depth API reference and code review it is unclear how exactly they function, what novel capabilities they offer, what parameters they use and what their limitations are.
- The three example applications should be accompanied by detailed tutorials with step by step instructions that can be followed to exactly reproduce the presented data and figures.
- It is not immediately clear to the reader how the workflows used to analyze the three exemplary applications differ and what elements of the pipeline they employ. It would be very helpful to provide a schematic for each detailing the data and modules used and stating key parameters where necessary.

In addition, the following should be considered by the authors:
- The example analyses lack key quality metrics that should be made accessible in the extended figures or tables including data completeness, CVs, intensity distributions, sample clustering before imputation, …
- Fig 2h is complex and most of the visualization elements does not aid understanding a more basic format may be more accessible. It is further unclear how proteins enriched in two groups are colored.
- Was the clustering and correlation analysis of the lung cancer data performed before or after imputation? How much is the correlation influenced by the proteins missing in a group and therefore full imputed in this group?
- In the discussion the authors claim “Instead of excising single cells, cellular neighborhood-guided proteome profiling represents a more robust and streamlined approach for phenotype-resolved discovery proteomics.” There is no data or reference substantiating this claim. It would excellent if the authors would compare the biological insight gained from analyzing the same are on consecutive sections by single cell proteomics vs neighborhood guided mini-pools.
- The authors should review the author list and author contributions. Some author contributions have likely been missed as several authors are listed solely for contributions that do not constitute intellectual contributions (e.g. resources and funding acquisition) and do not warrant authorship. These two sections need to align.
- Considering that the authors developed a new cyclic IF staining protocol it would be important to include an extended figure with the relevant controls (at least for the markers used here). Figure 4b is not sufficient to fully evaluate the quality of this method.

Minor comments:
- Gene names should be italicized to distinguish them from proteins (e.g. line 286)
- Resolution for some figures (eg. extended fig 3g) not adequate
- Direct links to the API reference and tutorial should be included at the beginning of the results section for easy accessibility
- Outline colors indistinguishable in fig 5o
- For most heatmaps details on the distance metric and linkage are missing

- Features in plots (e.g. whiskers in box plots) are largely undefined or information not easily accessible
- Fig 3g – the volcano plot for CosMX RNA data appears to use p-values. The authors should consider using corrected q-values or FDR or clearly state why this correction was not necessary.

Reviewer #4 (Remarks on code availability):

The code is well structured, readable and well annotated.
I have not tested installation, running and reproducibility of the manuscript data.

Version 1:

Decision Letter:

Our ref: NMETH-A62009A

19th Mar 2026

Dear Fabian,

Thank you for submitting your revised manuscript "OpenDVP: An experimental and computational framework for community-empowered deep visual proteomics" (NMETH-A62009A). It has now been seen by the original referees and their comments are below. The reviewers find that the paper has improved in revision, and therefore we'll be happy in principle to publish it in Nature Methods, pending minor revisions to satisfy the referees' final requests and to comply with our editorial and formatting guidelines.

1. Please submit a point-by-point rebuttal to the remaining comments when you resubmit the paper.
2. We will overrule the comment from Ref 1 about removing cnDVP from the manuscript. Please do not remove any data.

We are now performing detailed checks on your paper and will send you a checklist detailing our editorial and formatting requirements within two weeks or so. Please do not upload the final materials and make any revisions until you receive this additional information from us.

TRANSPARENT PEER REVIEW
Nature Methods offers a transparent peer review option for new original research manuscripts submitted from 17th February 2021. We encourage increased transparency in peer review by publishing the reviewer comments, author rebuttal letters and editorial decision letters if the authors agree. Such peer review material is made available as a supplementary peer review file. **Please state in the cover letter 'I wish to participate in transparent peer review' if you want to opt in, or 'I do not wish to participate in transparent peer review' if you don't.** Failure to state your preference will result in delays in accepting your manuscript for publication.

Please note: we allow redactions to authors' rebuttal and reviewer comments in the interest of confidentiality. If you are concerned about the release of confidential data, please let us know specifically what information you would like to have removed. Please note that we cannot incorporate redactions for any other reasons. Reviewer names will be published in the peer review files if the reviewer signed the comments to authors, or if reviewers explicitly agree to release their name. For more information, please refer to our FAQ page.

ORCID
IMPORTANT: Non-corresponding authors do not have to link their ORCIDs but are encouraged to do so. Please note that it will not be possible to add/modify ORCIDs at proof. Thus, please let your co-authors know that if they wish to have their ORCID added to the paper they must follow the procedure described in the following link prior to acceptance:
https://www.springernature.com/gp/researchers/orcid/orcid-for-nature-research

Author names using non-Roman characters
Nature Portfolio journals can support presentation of author names using non-Roman characters in the HTML version of the article. If you wish to, please include author names in parentheses after the Roman-character spelling; see example online here. Currently supported scripts are: Arabic, Chinese, Cyrillic, Devanagari, Greek, Hebrew, Hangul, Japanese and Persian. You will be asked to verify the rendering is correct at proof stage.

Thank you again for your interest in Nature Methods. Please do not hesitate to contact me if you have any questions. We will be in touch again soon.

Sincerely,
Madhura

Madhura Mukhopadhyay, PhD
Senior Editor
Nature Methods

Reviewer #1 (Remarks to the Author):

The key concept of DVP is image-guided automated single-cell segmentation. Critical algorithm components of DVP workflow include whole-slide single cell segmentation (e.g., Cellpose, BIAS), mask file conversion / importation into LMD7 system (e.g., py-LMD), and multi-modal data alignment and visualization. In this study, openDVP aims to develop “an open-source framework providing flexible, interoperable workflows that support simplified experimental strategies for users with limited computational expertise and advanced pipelines optimized for scalability and complex analyses.” In this revision, the authors provided detailed responses to my comments. However, the responses are quite general for most of the critical points and didn’t make significant improvement to these critical points.
1. Current openDVP framework doesn’t provide any novel critical algorithm components, but assemble the existing ones for whole-slide single cell segmentation, mask file conversion / importation into LMD 7 system and down-stream MS data analysis. In this revision, the authors provided “an in-depth API documentation site, and guidelines for using the different functions of the openDVP package.” After tested by two researchers with computation science and DVP experience, the package can now be properly installed but is far from a whole integrated workflow for users with limited computational expertise. Current package can only start from exiting well-segmented mask file. The user needs to code additional glue scripts to accomplish the whole task.
To this end, the authors should reorganize their figure presentation to focus on the key novelty of this study for providing a user-friendly pipeline rather than provide comprehensive application without biological validation. As reviewer 4 highlighted, “the three example applications should be accompanied by detailed tutorials with step-by-step instructions that can be followed to exactly reproduce the presented data and figures.” Without achieving this user-friendly and integrated workflow from raw whole slide image segmentation, properly aligned mask file into LMD7 system to multi-modal omics data visualization, the novelty of this study is limited. The authors should provide a unified stand-alone graphic user interface to fully reproduce all the three applications.
2. As my comment and Reviewer 2’s comment pointed out “The POI-based approach raised the question of whether the presented methods are still an iteration of Deep Visual Proteomics……”, the authors failed to achieve plasma membrane based single cell segmentation, although they have performed surface marker staining such as CD3E. The performance of openDVP for single cell segmentation is therefore questionable. Along the line, the authors nicely compared the performance of Cellpose, QuPath and BIAS for nuclei segmentation with acceptable performance. Revision Fig. 2 demonstrated nice whole slide imaging segmentation. However, the authors didn’t provide the data for proper whole slide contour transfer for laser microdissection as demonstrated in Revision Fig. 1, raising the question of their performance for whole-slide single cell mask file conversion / importation accuracy. The authors should provide quantitative evaluation as they did for nuclei segmentation.
3. The cnDVP concept is practically useful but pretty much an imaging-guided mini-bulk spatial proteomic analysis, losing single-cell-type resolution which is the key concept of DVP. Such type of application doesn’t need rely on mask file with irregular shape and deviates from the key topic of this study for developing open-source DVP framework. I would suggest the authors to move these nice data to a separated story and expand the figure presentation as highlight in point 1.

Reviewer #1 (Remarks on code availability):

Included

Reviewer #2 (Remarks to the Author):

The revised manuscript shows substantial improvement and addresses the majority of the reviewers' concerns, thank you! The addition of systematic comparisons between DVP approaches (DVP, scDVP, and cnDVP) in new Figure 5 and Ext. Data Fig. 5 particularly strengthens the work. The manuscript is now even more than before an important contribution to the spatial proteomics field by providing an open-source (“democratic”), community-driven framework for DVP. The systematic comparison of DVP approaches is valuable and will help users select appropriate methods. A few minor clarifications are needed in my view before publication.

(1) The novel point that cnDVP "improves" proteome coverage compared to standard DVP should be reconsidered in my view. Given equivalent tissue input (100 cells), there is no clear mechanistic basis for increased protein identifications with cnDVP. If the observed difference reflects improved collection efficiency for single large shapes versus many small shapes (as noted in the authors' response regarding electrostatic losses), this should be stated explicitly in the manuscript.

(2) I am critical that the “improved” proteome coverage by cnDVP is in fact a desirable experimental property. An explanation could be that the ROI-based approach in cnDVP merges signals from different cell types and extracellular matrix, which would inevitably lead to higher protein IDs. However, these come at the expense of dilution of the biological signal. Thus, I think the claim of improved proteome coverage needs to be tempered, omitted or very well discussed at the moment it is stated in the manuscript.

(3) Related to that, did the authors investigate which proteins were uniquely identified by cnDVP (Extended Data Fig. 5b)?

Specifically, what is the subcellular localization of these proteins? Did the authors observe any enrichment of extracellular matrix components that could be attributed to the ROI-based nature of cnDVP, or any effects on membrane proteins that could relate to differential laser microdissection damage between approaches?

(4) Related to the comparison of DVP approaches, did the authors track the sample dropout rate for cnDVP? This is relevant because the failure modes differ fundamentally between approaches. In standard DVP, if a fraction of the 100 individual shapes fails to collect (for instance, 90%), the pooled sample remains usable with marginally reduced input (and less protein IDs). In cnDVP, however, failure to collect the single ROI results in complete sample loss. The manuscript states that 203 samples remained "after QC filtering" but I could not find how many were initially attempted. Transparency about dropout rates would help users assess the practical trade-offs, particularly given that cnDVP is rated as "best" for "Ease of Proteomic Workflow" in the radar plot (Fig. 5f).

(5) The term "cellular neighbourhood" derives from computational spatial analysis tools such as graph-based spatial networks and describes precise node-edge networks between cells as atomic units. This is not how the authors use it here, but here the authors rather refer to something like a niche (EMT niche, tumor niche, immune niche). For consistency and to better convey the intended biological interpretation, consider replacing "cellular neighborhood" with "cellular niche" or "spatial niche" throughout.

(6) Please specify the total number of single cells analyzed by the scDVP pipeline. At line 400 or so, the authors mention that "50 single immune cells" were analyzed, but it remains unclear whether these 50 cells were included in the comparison with the other methods.

Reviewer #3 (Remarks to the Author):

I co-reviewed this manuscript with one of the reviewers who provided the listed reports. This is part of the Nature Methods initiative to facilitate training in peer review and to provide appropriate recognition for Early Career Researchers who co-review manuscripts.

Reviewer #4 (Remarks to the Author):

The authors have substantially revised the manuscript and added new data.
In doing so most comments of the reviewers were addressed and the clarity of the manuscript was improved.

One additional minor comment that was missed in my initial review is that the authors state that they use low bind 384 plates. The part number then shows that they are using DNA low bind tubes rather than protein low bind tubes. This should be stated in the text.

Reviewer #4 (Remarks on code availability):

Not reviewed in the revised version.

Version 2:

Decision Letter:

9th Sep 2026

Dear Dr Coscia,

I am pleased to inform you that your Article, "OpenDVP: An experimental and computational framework for community-empowered deep visual proteomics", has now been accepted for publication in Nature Methods. The received and accepted dates will be 23rd Jul 25 and 9th Sep 26. This note is intended to let you know what to expect from us over the next month or so, and to let you know where to address any further questions.

Over the next few weeks, your paper will be copyedited to ensure that it conforms to Nature Methods style. Once your paper is typeset, you will receive an email with a link to choose the appropriate publishing options for your paper and our Author Services team will be in touch regarding any additional information that may be required. It is extremely important that you let us know now whether you will be difficult to contact over the next month. If this is the case, we ask that you send us the contact information (email, phone and fax) of someone who will be able to check the proofs and deal with any last-minute problems.

After the grant of rights is completed, you will receive a link to your electronic proof via email with a request to make any corrections within 48 hours. If, when you receive your proof, you cannot meet this deadline, please inform us at rjsproduction@springernature.com immediately.

Authors may need to take specific actions to achieve compliance with funder and institutional open access mandates. If your research is supported by a funder that requires immediate open access (e.g. according to Plan S principles or the <a

href=“https://www.springernature.com/gp/open-science/us-federal-agency-compliance”> NIH public access policy) then you should select the gold OA route, and we will direct you to the compliant route where possible. Because authors warrant under our subscription licensing terms that they haven’t committed to licensing any version of their article under a licence inconsistent with the terms of our agreement – including the applicable embargo period – publication under the subscription model isn’t suitable for authors whose funders require no embargo.

If you have any questions about our publishing options, costs, Open Access requirements, or our legal forms, please contact ASJournals@springernature.com

If you have posted a preprint on any preprint server, please ensure that the preprint details are updated with a publication reference, including the DOI and a URL to the published version of the article on the journal website.

You may wish to make your media relations office aware of your accepted publication, in case they consider it appropriate to organize some internal or external publicity. Once your paper has been scheduled you will receive an email confirming the publication details. This is normally 3-4 working days in advance of publication. If you need additional notice of the date and time of publication, please let the production team know when you receive the proof of your article to ensure there is sufficient time to coordinate. Further information on our embargo policies can be found here:
https://www.nature.com/authors/policies/embargo.html

To assist our authors in disseminating their research to the broader community, our SharedIt initiative provides you with a unique shareable link that will allow anyone (with or without a subscription) to read the published article. Recipients of the link with a subscription will also be able to download and print the PDF.

As soon as your article is published, you will receive an automated email with your shareable link.

If you are active on Twitter/X or Bluesky, please e-mail me your and your coauthors’ handles so that we may tag you when the paper is published.

You can now use a single sign-on for all your accounts, view the status of all your manuscript submissions and reviews, access usage statistics for your published articles and download a record of your refereeing activity for the Nature journals.

Please note that you and any of your coauthors will be able to order reprints and single copies of the issue containing your article through Nature Portfolio's reprint website, which is located at http://www.nature.com/reprints/author-reprints.html. If there are any questions about reprints please send an email to author-reprints@nature.com and someone will assist you.

Please feel free to contact me if you have questions about any of these points.

Best regards,
Madhura

Madhura Mukhopadhyay, PhD
Team Leader and Senior Editor
Nature Methods

** Visit the Springer Nature Editorial and Publishing website at www.springernature.com/editorial-and-publishing-jobs for more information about our career opportunities. If you have any questions please click here.**

Open Access This Peer Review File is licensed under a Creative Commons Attribution 4.0 International License, which permits use, sharing, adaptation, distribution and reproduction in any medium or format, as long as you give appropriate credit to the original author(s) and the source, provide a link to the Creative Commons license, and indicate if changes were made. In cases where reviewers are anonymous, credit should be given to 'Anonymous Referee' and the source.
The images or other third party material in this Peer Review File are included in the article's Creative Commons license, unless indicated otherwise in a credit line to the material. If material is not included in the article's Creative Commons license and your intended use is not permitted by statutory regulation or exceeds the permitted use, you will need to obtain permission directly from the copyright holder.
To view a copy of this license, visit https://creativecommons.org/licenses/by/4.0/

<u>Detailed point-by-point response to the Reviewers' comments</u>

We thank the Editor and the Reviewers for their constructive comments and positive feedback on our manuscript ***"OpenDVP: An experimental and computational framework for community-empowered deep visual proteomics"***.

Following the Reviewers' comments, we have performed additional experiments, included new data and explanations to more rigorously benchmark and validate the key components of the openDVP workflow. Particularly, we focused on image-processing and laser microdissection (LMD) and benchmarked the new cellular neighborhood-guided DVP method with a systematic comparison to our original workflow (Mund & Coscia *et al*, Nat. Biotech, 2022) [1] and the single-cell strategy introduced by Rosenberger *et al* (Nature Methods, 2023) [2]. These new data are presented in two new figures (Fig.5 and Ext. Data Fig. 5) including a summarizing method comparison to support users choosing the best DVP strategy for their own application. We have also significantly revised the main text, discussion, figures and methods where the Reviewers felt it needed more clarification or detail. Please find below the answers to each of the Reviewers' comments.

Reviewer #1 (Remarks to the Author):

This manuscript describes OpenDVP, an open-source framework for deep visual proteomics that integrates image preprocessing, AI-driven segmentation/phenotyping, LMD export, and downstream MS analysis. The idea is timely and potentially valuable to the proteomics community, but the submission currently lacks critical technical detail and quantitative evaluation. Before the work can be considered for publication, the authors should provide stronger evidence of methodological novelty, robustness across imaging and LMD platforms, and quantitative validation of each stage in the image-to-proteomics workflow.

We thank the Reviewer for the constructive feedback and appreciate the encouraging words that our work is timely and valuable to the proteomics community upon additional technical and quantitative evaluation.

General Concerns

1. Lack of technical details and unclear innovation. The technical description of the OpenDVP platform is overly simplistic. Beyond the integration of existing algorithms, **what are the core innovations** of OpenDVP for the DVP workflow? The authors should explicitly present them with related data support.

We thank the reviewer for this valuable comment. In the revised manuscript, we have substantially expanded the Methods and Results sections to clearly articulate these contributions and now provide additional data supporting each point. Most importantly, we now provide two new figures (Fig. 5 and Ext. Data Fig. 5) to show the versatility of openDVP to support different experimental workflows. The core innovations of openDVP are summarized below. We have also revised the Discussion to better articulate those.

1. First open-source and extensible DVP framework

OpenDVP is the first open-source deep visual proteomics platform. Existing solutions are proprietary and closed, limiting extensibility, reproducibility, and community-driven development. OpenDVP introduces an openly accessible and modular architecture that allows users to integrate new algorithms, customize workflows, and ensure full transparency of the DVP process.

2. Rigorous focus on quality control, interactive visualization, and interoperability

OpenDVP uniquely embeds standardized quality control procedures, interactive high-dimensional visualization, and analytic tooling aligned with the scverse ecosystem. This ensures that workflows are fully reproducible, interoperable, and consistent with community standards—capabilities not available in current solutions. To our knowledge, this is the first instance of integrating mass spectrometry-based (spatial) proteomic data into the scverse ecosystem.

3. First framework enabling multimodal integration of imaging and mass spectrometry data

OpenDVP provides the first unified framework capable of integrating multimodal data (e.g., imaging and transcriptomics) with mass spectrometry–based molecular readouts. This multimodal linkage through SpatialData [3] enables richer biological interpretation and supports downstream computational tasks such as co-localization, phenotype refinement, and spatial–molecular correspondence analyses.

4. New fast-track "flashDVP" strategy based on our newly developed QuPath-to-LMD webapp supporting a growing user community

We now include more technical details for our fast-track pipeline from QuPath annotations to laser microdissection (LMD). This has already emerged as an important entry point for the expanding spatial proteomics community, lowering initials barrier to broad adoption and increasing throughput of DVP-based discovery workflows.

5. Introduction of cellular neighborhood–guided DVP and comprehensive new benchmark dataset for method comparison of the DVP universe

We now better describe and benchmark our novel strategy that uses cellular neighborhood information to guide deep spatial proteome profiling. This approach substantially accelerates spatial discovery proteomics in tissues. Data demonstrating improved throughput and proteome coverage are included in the revised manuscript (Fig. 5 and Extended Data Fig. 5).

We believe that these additions clarify the core innovations of openDVP beyond the integration of existing algorithms and provide strong data-driven evidence of the platform's technical and experimental contributions. We thank the Reviewer again for prompting us to strengthen this section. Please see more specific points below.

2. Absence of quantitative benchmarking for key algorithms. Several critical algorithms—such as cell segmentation, mask file generation, and real Laser Capture Microdissection (LMD) performance—lack rigorous quantitative comparison against

established state-of-the-art methods. This omission makes it difficult to objectively assess their performance and advantages.

We thank the Reviewer for this important comment and agree that quantitative benchmarking is essential. In the revised manuscript, we have added systematic performance comparisons for key workflow steps.

First, we benchmarked cell segmentation accuracy (Table below) using representative images from the breast cancer dataset (Fig. 4). Manually annotated nuclei were used as ground truth to compare segmentation performance across Cellpose, QuPath cell detection, and one generic BIAS model (generic_nucleus_seg_v1.0). Cellpose and QuPath showed robust and comparable performance, whereas the BIAS model performed substantially worse, particularly on very dense immune cell areas. However, please note that segmentation accuracy can be significantly improved with additional re-training using the same tissue (not done here). Since July 2025, both QuPath and BIAS offer more recent models, namely *InstanSeg* and CellposeSAM, respectively. These are also compatible with our openDVP pipeline and should further improve segmentation.

Revision Table 1: Segmentation accuracy metrics for DVP compatible softwares.

Segmentation Approach vs. ground truth	Dice Coefficient (Higher is better)	Jaccard Index (Higher is better)	Hausdorff Distance (Lower is better)
Cellpose 'nuclei' model	0.839	0.722	81.60
QuPath's Cell detection	0.797	0.663	56.22
BIAS' generic nucleus seg model (NucleAIzer)	0.7602	0.6131	48.26

Second, we evaluated the computational scalability of the recommended image analysis pipelines by comparing processing time and peak memory (RAM) usage across all major image processing steps, demonstrating efficient performance on large whole-slide datasets. These new data are included in Ext. Data. Fig. 1a-c.

Third, we assessed the accuracy of coordinate transfer between image analysis outputs and LMD software, demonstrating that appropriate calibration point selection is critical for precise tissue collection. Best-practice guidelines are now provided in the openDVP documentation to support reproducible implementation. These guidelines are also included in Ext. Data. Fig. 1d-e.

Finally, we quantitatively compared different LMD collection strategies, including our original DVP strategy (Mund & Coscia et al, Nat. Biotech, 2022), single-cell DVP (scDVP) (Rosenberger *et al*, Nature Methods, 2023), and cellular neighborhood–guided DVP (cnDVP, this manuscript), highlighting trade-offs between throughput, spatial resolution, and proteome depth. These data are included in a new main Fig. 5 and Ext. Data. Fig. 5.

We believe these additions provide a rigorous and transparent benchmarking framework that enables objective assessment of openDVP's performance relative to established approaches.

3. Disconnected biological applications undermine systematic performance evaluation. The three presented biological applications appear too independent of each other, and their specific role in evaluating the platform's performance is not clearly defined, such as the scanning equipment. We recommend focusing on two applications and structuring the evaluation logically. A direct comparison is essential, particularly to highlight the the performance at each key step. Crucially, this evaluation should include direct, quantitative comparisons with equivalent modules from other modular software platforms to concretely position OpenDVP's performance within the existing landscape.

We thank the Reviewer for this important comment. The three biological applications were intentionally chosen to demonstrate the versatility of openDVP across complementary and commonly encountered spatial proteomics scenarios, as also appreciated by Reviewer 2. Specifically, the breast cancer tumor microenvironment study represents a segmentation- and phenotyping–dependent workflow, whereas the placenta and lung cancer examples illustrate segmentation-free entry points. Together, these cases demonstrate that openDVP supports both segmentation-based and segmentation-independent DVP strategies within a unified framework.

To clarify the role of each application in evaluating platform performance, we have revised the manuscript to explicitly link each use case to specific computational and experimental workflow components. In addition, we have added new quantitative benchmarks assessing segmentation which are now directly associated with the breast cancer application.

OpenDVP is designed to be independent of specific imaging hardware and supports open, BioFormats-compatible image formats (e.g., OME-TIFF). We clarify this explicitly in the main text (Fig. 1, line 123). All images in this study were acquired using a Zeiss Axioscan 7 slide scanner, supporting both brightfield and fluorescence imaging.

Where applicable, we now include direct quantitative comparisons at key workflow steps using functionally comparable modules. We emphasize that the primary contribution of openDVP is not the replacement of individual best-in-class algorithms, but their systematic integration and benchmarking within an open, end-to-end DVP framework.

We hope these revisions address the Reviewer’s concerns and clarify the rationale and evaluation strategy underlying the presented applications.

Technical Concerns

1. Novelty and Differentiation from Existing Tools (e.g., BIAS): The authors was involved in previously developed BIAS (Biology Image Analysis Software). To strengthen the Introduction, a more granular analysis of the limitations of existing software tools, including BIAS, is required. The authors should explicitly clarify the differences and novelty between OpenDVP and BIAS. Additionally, OpenDVP is presented as an integrative framework combining existing tools for DVP workflows; the technical innovations of OpenDVP need to be clearly articulated.

We fully agree that a clearer differentiation from existing tools, including BIAS (Biological Image Analysis Software), is needed. We have revised the Introduction to explicitly address this point in a new paragraph.

In a nutshell, both solutions are important and come with different trade-offs and interfaces. Through a new Python API interface and close collaboration with the Horvath group (BIAS developers), our future goal is therefore to enable cross-platform usage, integrating openDVP and BIAS workflows.

With respect to innovations, openDVP represents a conceptual and architectural advance. As outlined above, it is the first open-source, Python-based framework explicitly designed to support the entire DVP workflow within a unified, reproducible ecosystem. Built on scverse standards, openDVP introduces a common data model, modular APIs, FAIR-compliant outputs, and scalable execution on both local and high-performance computing infrastructures. Although openDVP intentionally integrates best-in-class existing tools rather than reimplementing individual algorithms, its technical innovations lie at the framework level, including standardized DVP workflow abstractions, support for segmentation-based and segmentation-free pipelines, multimodal integration of imaging and proteomics data, and systematic benchmarking across DVP modalities.

We believe these revisions clarify that openDVP is not an incremental extension of BIAS, but a foundational platform that enables reproducible, scalable, and community-driven deep visual proteomics.

2. Accuracy and Precision of the Cell Segmentation: The authors claimed that "OpenDVP provides a framework to process images into feature tables and analyze matrices to phenotype cells, using open-source tools." (Line 105-106). Cell segmentation, especially for the single cell plasma membrane, is very critical for DVP workflow. Since the author didn't develop their own cell segmentation method, the authors should provide quantitative comparison between existing methods such as CellPose, Mesmer, Cellseg, etc. A clear description how these tools can be efficiently applied through OpenDVP as compared with direct application. The term "deep" in deep visual proteomics refers to both deep learning and high-coverage proteomic analysis. The authors should highlight the performance of the AI-driven tools they properly evaluated.

We agree that cell segmentation is a critical step in bioimage analysis in general, and DVP in particular. However, we note that all segmentation methods supported by openDVP have been rigorously benchmarked in separate stand-alone publications (see Greenwald *et al.*, 2022; Stringer *et al.*, 2021). We provide detailed guidelines how these algorithms are implemented in openDVP.

As pointed out above, we have additionally quantitatively compared the performance of different cell segmentation strategies (Cellpose, QuPath, and BIAS) to a subset of our breast cancer imaging data. Cellpose and QuPath showed robust and comparable performance, whereas the BIAS model performed substantially worse, particularly on very dense immune cell areas. However, please note that segmentation accuracy can be significantly improved with additional re-training using the same tissue (not done here). Since July 2025, both QuPath and BIAS offer more recent models, namely *InstanSeg* and CellposeSAM, respectively. These are also compatible with our openDVP pipeline and

should further improve segmentation.

Using scimap's spatialLDA algorithm, seven cellular neighborhoods (CNs) are divided according to cell distribution, with each shown as a square. How is the accuracy of this segmentation ensured?

The accuracy of segmentation is ensured by visual inspection of the overlay of segmentation masks and multiplex immunofluorescence images. This can be done interactively in Napari or QuPath.

We noticed that in Extended data Fig.4e, the algorithm-based cell segmentation does not appear fully accurate at single-cell resolution. For instance, in the CD3E and Vimentin staining panels, the recognized cell contours do not precisely align with the actual staining signals.

These inconsistencies most likely arise because we did not perform cytoplasmic segmentation. Instead, we chose nuclear segmentation with pixel expansion (5 pixels = 1.7um) for segmenting our tissue, for its consistency across 1.6 million cells and due to the limited mIF panel including only a few cell surface markers. However, openDVP also offers cytoplasmic/whole cell segmentation though the latest Mesmer and Cellpose models. We think cytoplasmic segmentation is particularly useful with higher-plex IF data (e.g., 15+ markers).

Specifically, the authors highlighted that segmenting about a million cells from centimeter-scaled whole slide images in less than 3 hours. The authors need to specify if this refers to brightfield or multi-color images, as segmentation times differ significantly. The stated time should exclude fine-tuning, as initial results are often suboptimal.

We now provide more details and specific metrics on the performance of cell segmentation in Extended Data Fig. 1a-c. The segmentation is based the first nuclear stain channel in our image dataset.

3. The gating function needs validation on more complex cell types with advanced metrics: OpenDVP provides interactive annotation and quality control via the napari-cell-gater plugin in Napari. However, the gating is overly simplistic, relying only on area and marker intensity, with limited discrimination power. The authors should provide more details about this critical step for filtering out proper single cells for downstream LCM and demonstrate more challenging examples, such as gating small immune cells in tumor microenvironments.

We now provide more details in the Methods section for each of the Napari filtering steps. The gating allows users to plot cells by choosing between any of the measured marker intensity and morphology metrics for either x- or y-axes. We have also improved the GitHub documentation on those critical guidelines.

4. Compatibility with LMD Platforms: The authors mention that this platform supports both LMD7 and MMI systems (Line162-163) which is nice. However, the authors fail to provide related details about how the mask file can be properly import into both systems and match with the real tissue slice. Quantitative data (e.g., on cutting accuracy, precision, and protein yield) obtained using the LMD7 and the MMI system should be provided, especially with single cell resolution. Related practical insight would greatly benefit user implementation the method like QuPath-to-LMD.

We have performed an additional experiment to assess the accuracy of LMD contour transfer at different magnifications (20x and 40x) for multi-cellular ROIs and single cells. As contour import is critically dependent on the accurate selection and use of reference points in the tissue, we now provide more details on this critical step. We have expanded our QuPath-to-LMD tutorial to guide users through the critical steps of reference point definition and contour transfer. On the Leica LMD7 system, three reference points are needed for contour import. As shown in our new Extended Data Fig. 1d-e (Revision Fig. 1 below), poor calibration points (i.e., too far away, too large, or too difficult to detect in the tissue) will lead to larger contour shifts, which is more pronounced at higher magnification. Well-chosen reference points instead result in much smaller shits (see Figure below). It is noteworthy that perfect contour alignment is often difficult to achieve due to tissue distortion or expansion through multiple tissue processing steps (staining, cover glass removal, air-dying etc.). The image used to segment the cells is typically acquired much in advance and before air-drying the samples for LMD. However, residual inaccuracies are typically corrected through a global, manual correction step directly in the LMD software. For this, all imported contours per field of view are selected and aligned based on the ground truth from the live microscopy image.

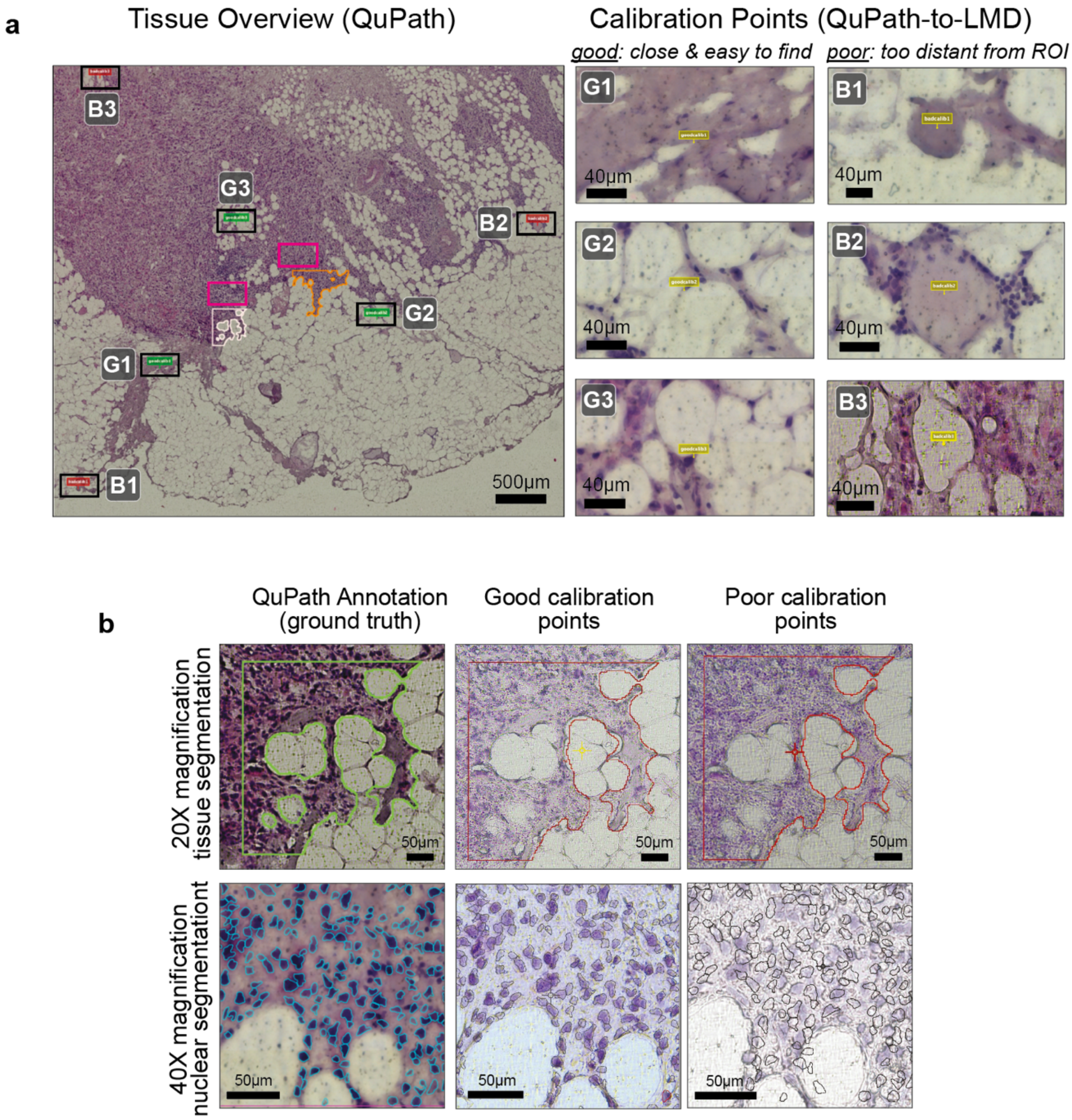

Revision Fig. 1: Accurate reference point selection enables reliable contour transfer for laser microdissection. (a) Tissue overview and reference point definition in QuPath. G1–G3 indicate three good calibration points, located close to the objects of interest and easily identifiable. B1–B3 indicate three poor calibration points, which are located farther away and are difficult to recognize. The center shows four annotated regions: two magenta boxes corresponding to regions subjected to nuclear (H&E-based) segmentation in QuPath, and two irregular regions corresponding to tissue segmentation annotations. (b) Accurate annotation transfer depends on reliable calibration points. Top row: large tissue segmentation annotation and its contour transfer to the LMD7. Poor calibration points result in an upward shift of the entire contour. Bottom row: nuclear segmentation and contour transfer. In this case, poor calibration points lead to relatively large positional errors, which would result in improper single-cell collection.

Regarding the MMI system, our laboratory currently does not have direct access to an instrument. However, through prior collaboration with MMI, we have validated that openDVP-generated JSON contour files are compatible with the MMI contour import functionality. While this confirms technical compatibility, we agree that more extensive benchmarking and user-oriented documentation would be valuable. We therefore plan to

expand and release dedicated MMI-specific documentation following additional validation experiments, which are scheduled for early 2026.

Finally, with respect to proteomic performance and protein yield, our new Fig.5 and Extended Data. Fig.5 addresses this specifically by comparing single-shape proteomes to cellular neighborhood-based ROIs (one 100-cell shape) and the standard DVP approach (hundred single-cell shapes).

5. Reproducibility Issues with the GitHub Repository and Tutorials:
When we test the OpenDVP method, we encountered technical difficulties:
Issue 1: In Tutorial 1, execution halted after the following line, intended to convert a segmentation mask into a GeoDataFrame containing polygons:
gdf = dvp.io.segmask_to_qupath(path_to_segmentation, simplify_value=1, save_as_detection=True)
gdf.head()
The system was unable to locate or invoke any functions from the 'dvp.io' module, and this problem extended to all functions within it. An AttributeError was raised, indicating that the 'opendvp' module lacks an 'io' attribute.
Issue 2: Attempting installation via 'pip install opendvp' in a Python 3.11 environment proved unsuccessful. The process failed during the "getting requirements to build wheel" stage, resulting in a subprocess errors.
Issue 3: Using Python 3.13 allowed for a successful installation of opendvp. However, fundamental operations continued to fail. For instance, in tutorial2.py, the following call:
adata = dvp.io.DIANN_to_adata(# paths and parameters here)

We thank the Reviewer for pointing this out. We discovered an installation bug, which is now addressed. We also improved the continuous code testing to prevent any similar bugs from going unnoticed. Additionally, support can also be found on our openDVP GitHub page, where we inform about recent software updates and bug fixes.

Minor Concerns
1. Figure 4b shows poor image quality. Figure 4c has evident shading correction issues (uneven brightness) and focus problems (e.g., region 4). Under such imaging quality, quantitative assessment of segmentation accuracy is needed for downstream analysis.

For Fig. 4b, we now provide updated figures showing zoom-ins (4x magnification compared to before) of the same field of view with higher image quality. For Fig. 4c, we have now compared multiple fields of view from different regions in the tissue (Figure shown below). These results revealed no differences in segmentation accuracy.

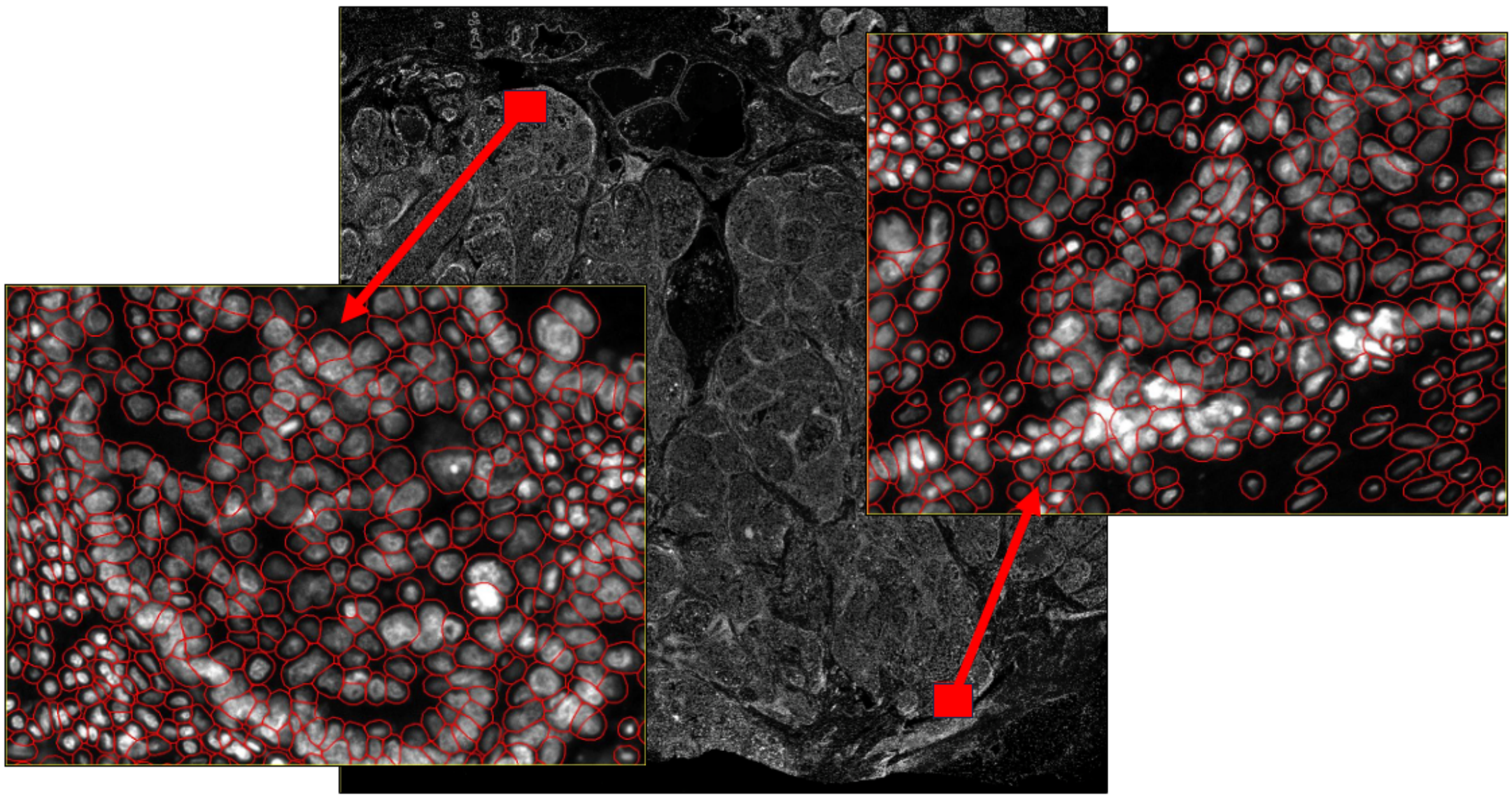

Revision Fig. 2: Consistent segmentation across entire whole-slide image. Here we show an overview of the primary breast cancer tissue and its nuclear DAPI stain. The two far apart regions show consistent segmentation results.

2. For the image data collected by OpenDVP, the first step is to convert it into TIFF format (Line 104, Line 125), followed by analysis using flashDVP or AI-driven image analysis. It is necessary to clarify: whether this format conversion process affects image quality; and whether it causes distortion, especially for complex multi-channel fluorescently stained images.

The image quality is not affected. The omeTIFF format uses no compression. Below is an example image showing a side-by-side comparison from before (Zeiss ZEN Blue software; left) and after conversion (openDVP TIFF format; right) for one tile.

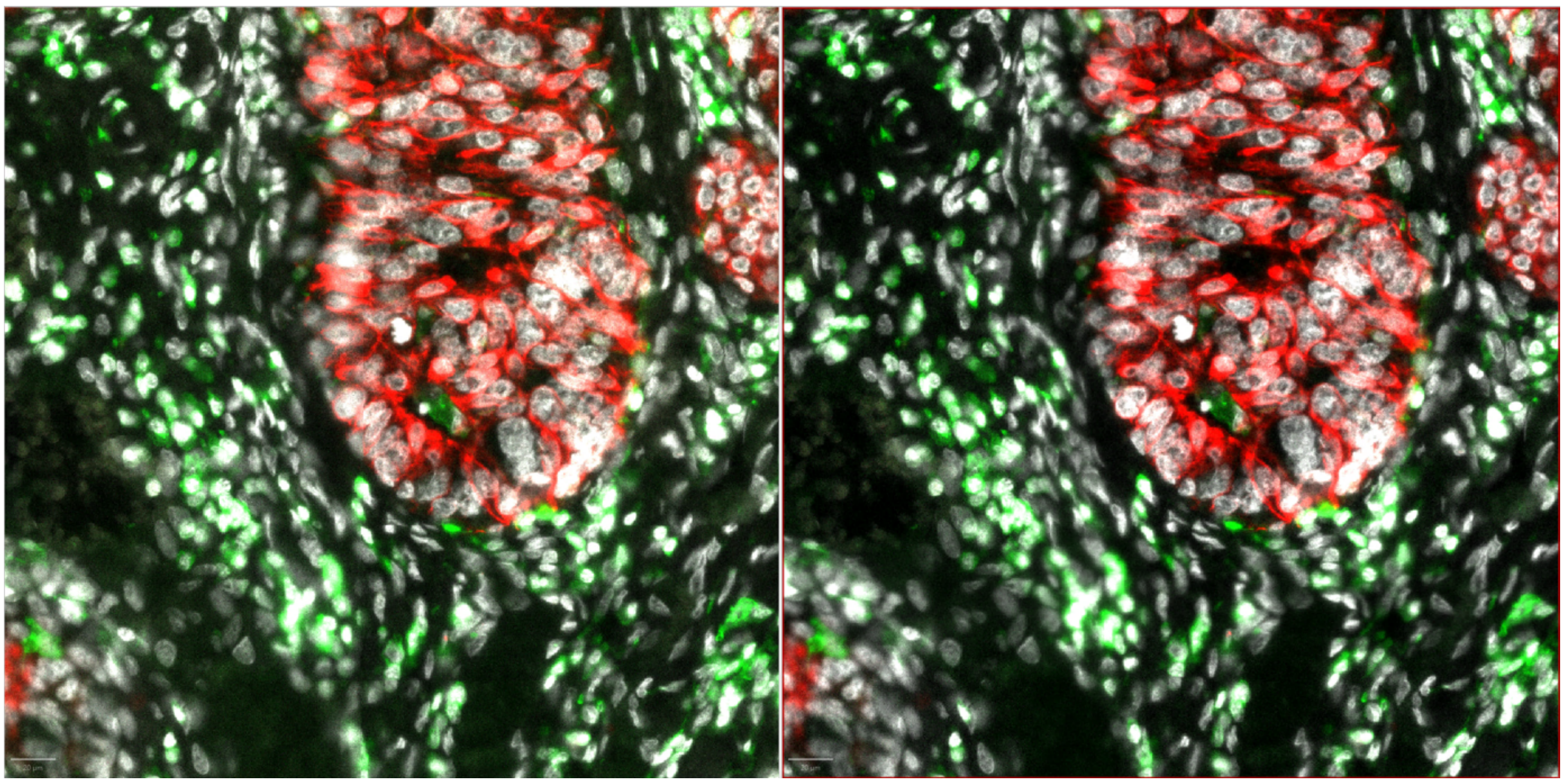

Revision Fig. 3: Image quality comparison between raw and processed data. On the left we see the raw .czi file, on the right the processed image (illumination corrected and stiched); all channels from both

images have the same minimum and maximum display values. White is the nuclear stain, green is CD3E, red is PanCK. CZI files are compressed at 14-bit, and are scaled to 16-bit in the OME-TIFF file.

3. The claim that “We annotated 150 cells per replicate for each cell type ... to reach an equal tissue amount per sample” (Lines 205-206) is misleading, as flashDVP does not perform image segmentation to obtain true cell counts.

Thank you for pointing this out. We have clarified this statement in the main text. We now write: “With QuPath, we manually annotated 150 cells per replicate...”

4. In Figure 5o, it’s not clear what do the two differently colored small circles represent.

We have now clarified this in the figure legend. These smaller circles related to the immune specific neighborhoods. Larger circles show tumor specific neighborhoods.

5. Line 36: scverse → scverse (single-cell universe ecosystem) for reader clarity.

We thank the Reviewer for highlighting the need to define this term for a broader audience. However, to our knowledge "scverse" is a project name rather than a strict acronym. We could not confirm “single-cell universe ecosystem” from the original publication.

6. Line 142: “Jupiter” should be written as “Jupyter”.

We have revised the text accordingly.

7. Line 344: The authors applied H&E staining of the same slide; how are fluorescent staining images aligned with H&E images?

We have added more details to describe the workflow. We used QuPath with the Warpy package for such alignment.

8. Line 346: “1'005,051” should be written as “1,005,051”.

We revised the text accordingly.

9. Line 389: “μm2” should be written as “μm^2”. Please thoroughly check the entire text.

We revised the text accordingly.

Reviewer #2 (Remarks to the Author):

The manuscript presents an open-access pipeline for deep visual proteomics (DVP) that integrates spatial proteomics data in a multimodal framework. This enables the mapping of proteomic signatures across regions of interest and cellular neighborhoods. A major strength of this work is its open, Python-based design, which builds on established software tools including MCMICRO, SOPA, QuPath, Napari, and SpatialData

and provides an end-to-end framework for proteomics and multimodal analysis. The authors introduce two complementary approaches for image analysis: flashDVP, a segmentation-free method designed for less experienced users and for analyzing syncytial tissue layers, and an advanced AI-based strategy capable of performing cell segmentation, phenotype classification, and neighborhood characterization. Three proof-of-concept case studies are provided to demonstrate the pipeline's applicability, two employing flashDVP and one the AI-driven approach.

The paper presents many exciting avenues that can make DVP a more mainstream application. It is timely and forward-looking, focusing on open source, FAIR principles, and popular tools readily adopted and, importantly, community-maintained. This makes DVP future-proof. The manuscript is well written and the tools are great, yet it is mostly a software-implementation effort and misses somewhat a scientific edge. Please find some specific comments and requests below.

We thank the Reviewer for the very positive evaluation of our manuscript and the encouraging words about the timely, forward-looking and future-proof nature of our work.

Major comments:
- The workflow moves DVP away from single-cell isolation in the original publication(s) to regions of interest. This is at times difficult to understand from the text, and I would recommend being clearer about what is based on single-shape isolation and what is ROI-based.

We thank the Reviewer for bringing up this important point. We now include an entire new Fig. 5 that we think addresses this concern through a systematic comparison of different DVP methodologies that are supported through openDVP. This includes our original DVP workflow (Mund & Coscia et al, Nat. Biotech, 2022)[1], single-cell DVP (Rosenberger et al. (Nature Methods, 2023)[2], and cellular neighborhood-guided DVP (cnDVP, this manuscript). We also clarified that our goal was not to move away from our original concept, but rather offering a versatile toolkit supporting different tissue collection strategies that each come with unique strengths and weaknesses. In our new Fig. 5f, we have summarized these strengths and weaknesses to support users selecting the best experimental design for their application.

In the revised manuscript, we also include more information on the chosen tissue isolation strategy for each of the datasets (ROI or single-contour based).

- The ROI-based approach raises the question of whether the presented methods are still an iteration of Deep Visual Proteomics, or rather laser microdissection with smart ROI selection that requires a different term. I find this very confusing as it will dilute the term DVP and make it also confusing for the community. Could the authors comment on this in the discussion? Currently, a technical advantage is presented which is that single shape-based approaches suffer from electrostatic forces impacting gravity-forced collection. I think other work in that field at least does not outline this as a problem.

Our new Fig.5 addresses this point in detail through a systematic comparison of different performance criteria relevant for spatial tissue proteomics experiments, such as proteome coverage, spatial resolution, workflow scalability and general ease of use. We now provide a radar plot to emphasize the unique strengths and weaknesses of each DVP

approach and also improved the Discussion. Importantly, openDVP supports all DVP-family approaches and provides a high degree of flexibility for different biological uses cases.

We fully agree that our novel cellular neighborhood guided approach should be named differently to distinguish it from pooling individually excised contours (original DVP concept). In the revised manuscript, we refer to it as cnDVP (cellular neighborhood DVP).

Tissue collection by gravity-based systems is inevitably influenced by electrostatics. It is influenced by several factors such as the tissue itself, the staining workflow and the used membrane slides. In our experience, this represents a particular challenge for small single shapes. Our summarizing radar plot comparing different DVP strategies includes this challenge under the category "Ease of Proteomic Workflow". For larger shapes this is issue is less pronounced and collection efficiencies are typically around 80 - 95%. Please also see our most recent publication by Klingeberg *et al* [4] where we systematically address this issue.

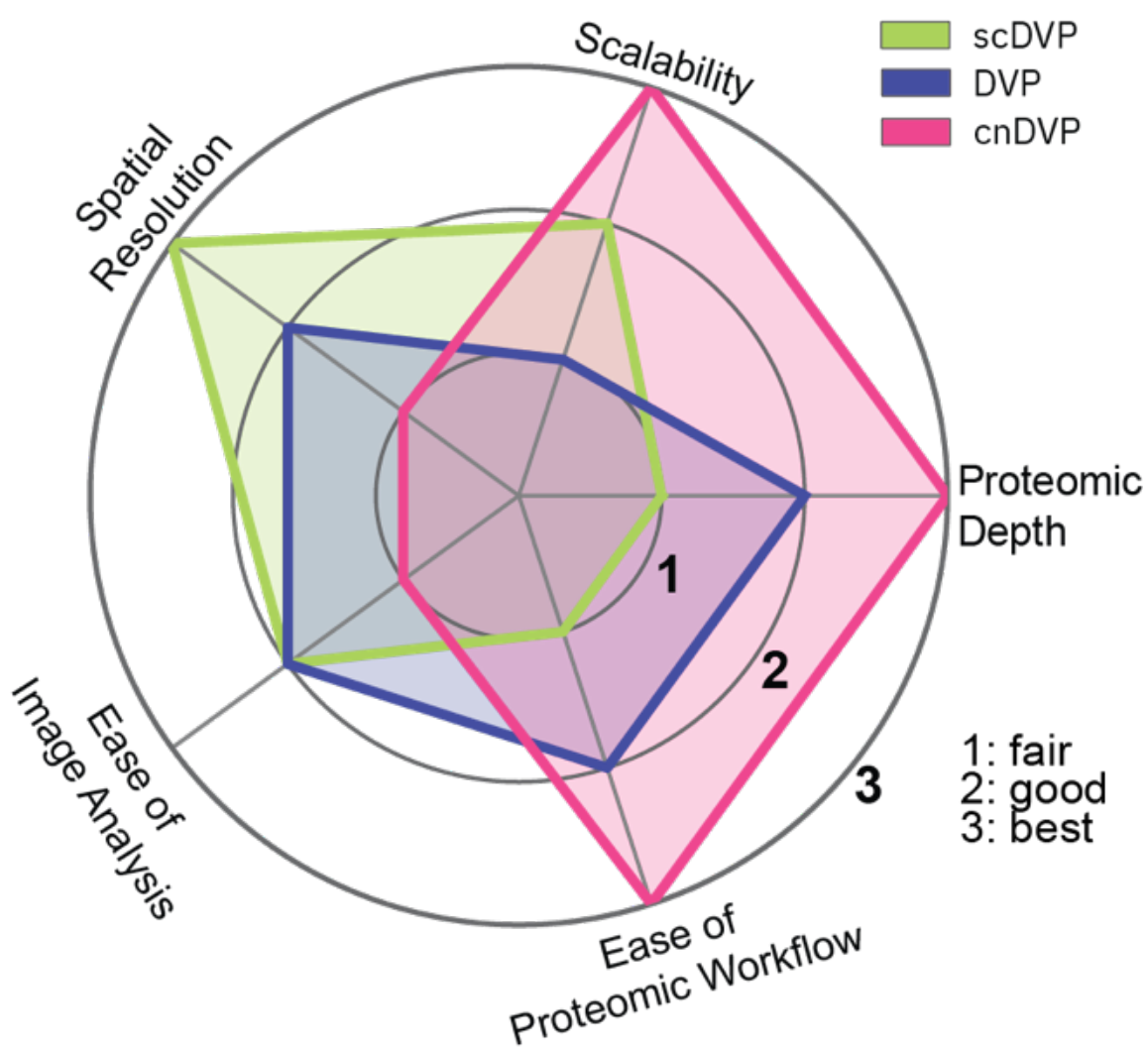

Revision Fig. 4: Radar plot comparing different DVP approaches. The radar plot summarizes the key differences between three DVP approaches; scDVP (light green), DVP (blue), and cnDVP (pink); across five key properties: Scalability, Proteomic Depth, Ease of Proteomic Workflow, Ease of Image Analysis, and Spatial Resolution. The radial axes represent a qualitative ranking scale where 1 = "fair", 2 = "good", and 3 = "best".

- The appeal of DVP is the cell-type enrichment, which is lost with an ROI-based approach, irrespective of the certainly smart way of selecting ROIs due to CNs or spatial transcriptomics. Can the authors convincingly show that their new avenue delivers as good biological data as cell-type enrichment? I think this is especially important if the workflow claims to be a universal toolkit for DVP. How does it then actually perform on a single-shape level (besides the manual single-shape selection in the placenta case study)?

We thank the Reviewer for this important point. OpenDVP includes a set of approaches that researchers can choose based on their biomedical application of interest. We now provide more detailed guidelines to compare those strategies.

In our new Fig. 5 and Extended Data Fig. 5, we have included a systematic comparison of DVP, cnDVP and scDVP using the triple-negative breast cancer (TNBC) tissue introduced in Fig. 4. We chose five adjacent regions for direct proteomic comparison and assessed multiple performance criteria: Technical challenges, collection speed, collection efficiency, and proteomic depth for the same tissue input area. Notably, all new data came from the exact same tissue sections that we stained and imaged more than one year ago (the proteome is remarkably stable in FFPE archival tissue, even after immunofluorescence staining). In our revised manuscript, we now include new data for single-shape proteomes of panCK+ tumor cells, which featured similar protein fold changes between the primary and relapse sample (Extended Data Fig. 5c). To our knowledge, this is the first example of measuring single-cell (i.e., single-shape) tumor proteomes obtained from archival FFPE tissue. Previous scDVP studies have mostly focused on significantly larger cell types, such as hepatocytes [2,5] . However, scDVP is currently limited to larger cell types and results in low proteome coverage for smaller cells, such as immune cells (Extended Data Fig. 5a). Applications are hence much more limited compared to our original DVP concept or the newly introduced cnDVP approach.

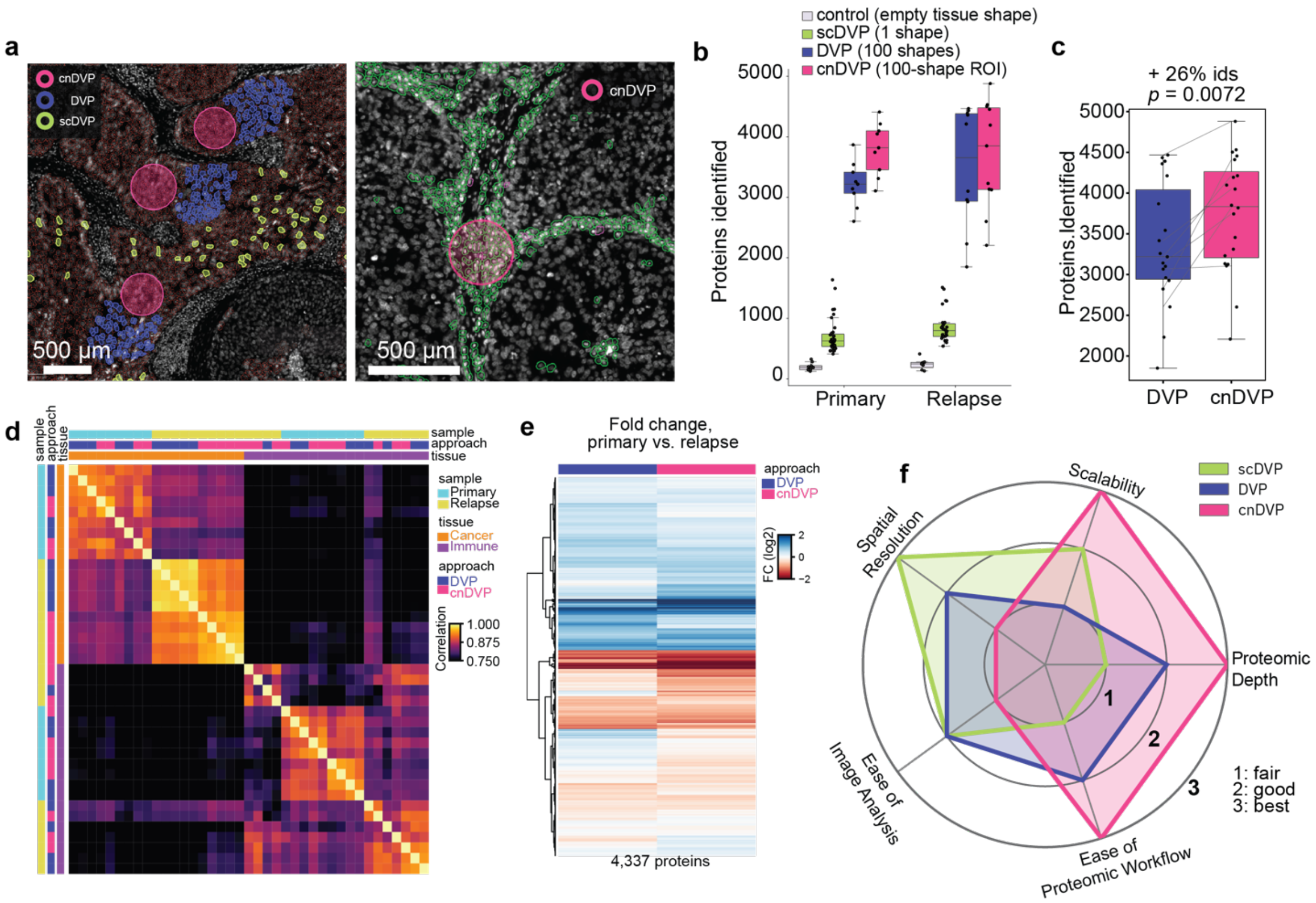

Revision Fig. 5: Cellular neighborhood guided DVP improves proteomic depth and throughput. (a) Images of primary TNBC tissue, showing the nuclear stain with segmentation masks for phenotyped cancer cells with red outlines on the left and CD3+ cells with green outlines on the right. Overlayed are collection annotations for the three DVP approaches. In magenta cnDVP, in blue DVP, and green for scDVP. **(b)** Boxplots with number proteins identified with DIANN search across DVP approaches. **(c)** Boxplot with number of proteins identified in matching cancer-specific samples analyzed by DVP and cnDVP. Lines denote spatial co-localization, showing an increase of proteome coverage using the cnDVP approach. P-value is based on a two-sided paired T-test. **(d)** Proteome correlation heatmap using Spearman correlations for all immune and cancer samples analyzed with DVP and cnDVP. **(e)** Heatmap of log2FC values for cancer samples between the primary and relapse breast cancer tissues across DVP and cnDVP approaches. Note, similar fold changes were retrieved for both approaches. **(f)** The radar plot summarizes the key differences

between three DVP approaches; scDVP (light green), DVP (blue), and cnDVP (pink); across five key properties: Scalability, Proteomic Depth, Ease of Proteomic Workflow, Ease of Image Analysis, and Spatial Resolution. The radial axes represent a qualitative ranking scale where 1 = "fair", 2 = "good", and 3 = "best".

- Cellular Neighborhoods are in principle a great idea, but here it lacks benchmarking that this is the smartest way to discover new biology. Can this be formalized and compared against an approach with fewer or more niches?

As shown above, we now provide benchmarking data for the TNBC study. The two included paired tissues (primary and local recurrent disease) offer an excellent use case for the cellular neighborhood comparison due to the vastly different tissue and microenvironment compositions. Notably, in both tissues, our results show that our novel cnDVP approach outperforms the classical DVP approach in terms of scalability and proteome coverage (Fig. 5b-c, Extended Data Fig. 5a,). For homotypic neighborhoods (dominated by one cell type), we generally recommend such strategy. We have also improved the main text and discussion to articulate the benefits and limitations of using cellular neighbourhood information to guide deep proteome profiling.

- The authors described a visual quality control check using the AI-based approach. However, it remains unclear how the accuracy of the ROIs was determined within the model. Did they apply cross-validation or any other systematic method to evaluate the accuracy of ROI classification across phenotypes?

Selected ROIs after cellular neighborhood analysis were directly annotated in the user-friendly QuPath software, which allows to overlay all image analysis data provided by openDVP (segmentation results, phenotype labels and cellular neighborhoods). We have clarified this in the main text and Methods. Our new proteomic data in Fig. 5 confirm the accuracy of such ROI based classification.

- For FlashDVP, the method uses manual annotation, which makes its scalability to large cohorts either very limited or highly time-consuming. To demonstrate true applicability in large-scale studies, why did the authors not consider developing a deep learning-based strategy (such as pixel-based methods) that can be trained to automatically identify ROIs? By integrating features such as shape, texture, and thresholds, the model could be trained effectively (while remaining independent of single-cell segmentation), and transfer learning could further enhance its applicability in large-scale settings.

We thank the Reviewer for this great suggestion. We fully agree that deep learning-based approaches (e.g., pixel-based methods and pathology foundation models) offer a very promising future addition and direction for openDVP. The Python-based open-source nature of our pipeline provides an excellent basis for such future developments.

- Similarly, the AI-based pipeline also seems to lack a transfer-learning step, limiting its adaptability and use in large-scale cohorts.

We fully agree that such approaches are exciting future areas, but beyond the scope of our current manuscript. We anticipate that a growing userbase will stimulate exciting

discussions on such innovative future extensions. OpenDVP was built to be versatile and modular in terms of the model used for steps such as nuclei/cell segmentation. Users are able to train their own models with their preferred tool and then scale up the implementation in a plug-and-play manner with the referenced pipelines.

- Using the QuPath-to-LMD workflow, ROI coordinates were transferred to the laser microdissection microscope. Please report the spatial accuracy of the contour transfer from image annotation to the laser microdissection at 20× and 63× magnification (i.e., how precisely do the annotated contours align with the actual microscope image), and describe how the QuPath-LMD interface was calibrated to ensure accurate contour transfer.

Please also see our response to Reviewer 1, point 4. We have performed an additional experiment to assess the accuracy of LMD contour transfer at different magnifications. We chose 20x and 40x as those are the most often used objectives in our experience, supporting multi-cellular ROIs and single-cell collection. As contour import is critically dependent on the accurate selection and use of reference points in the tissue, we now provide more details on this critical step. We have expanded our QuPath-to-LMD tutorial to guide users through the critical steps of reference point definition and contour transfer. On the Leica LMD7 system, three reference points are needed for contour import. As shown in our new Extended Figure 1d-e, poor calibration points (i.e., too far away, too large, or too difficult to detect in the tissue) will lead to larger contour shifts, which is more pronounced at higher magnification. Well-chosen reference points instead result in much smaller shits (see Figure below). It is noteworthy that perfect contour alignment is often difficult to achieve due to tissue distortion or expansion through multiple tissue processing steps (staining, cover glass removal, air-dying etc.). The image used to segment the cells is typically acquired much in advance and before air-drying the samples for LMD. However, residual inaccuracies are typically corrected through a global, manual correction step directly in the LMD software. For this, all imported contours per field of view are selected and aligned based on the ground truth from the live microscopy image.

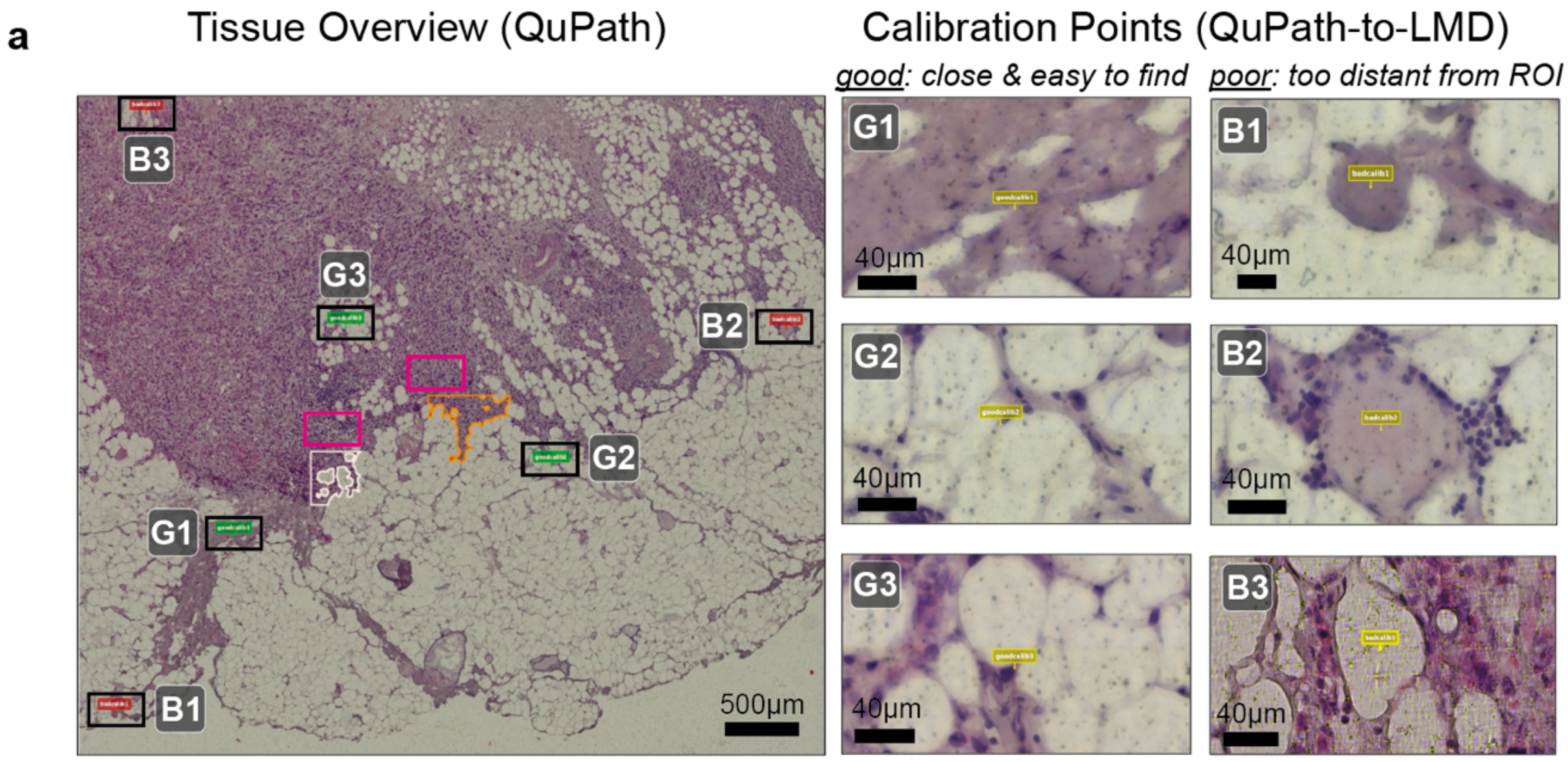

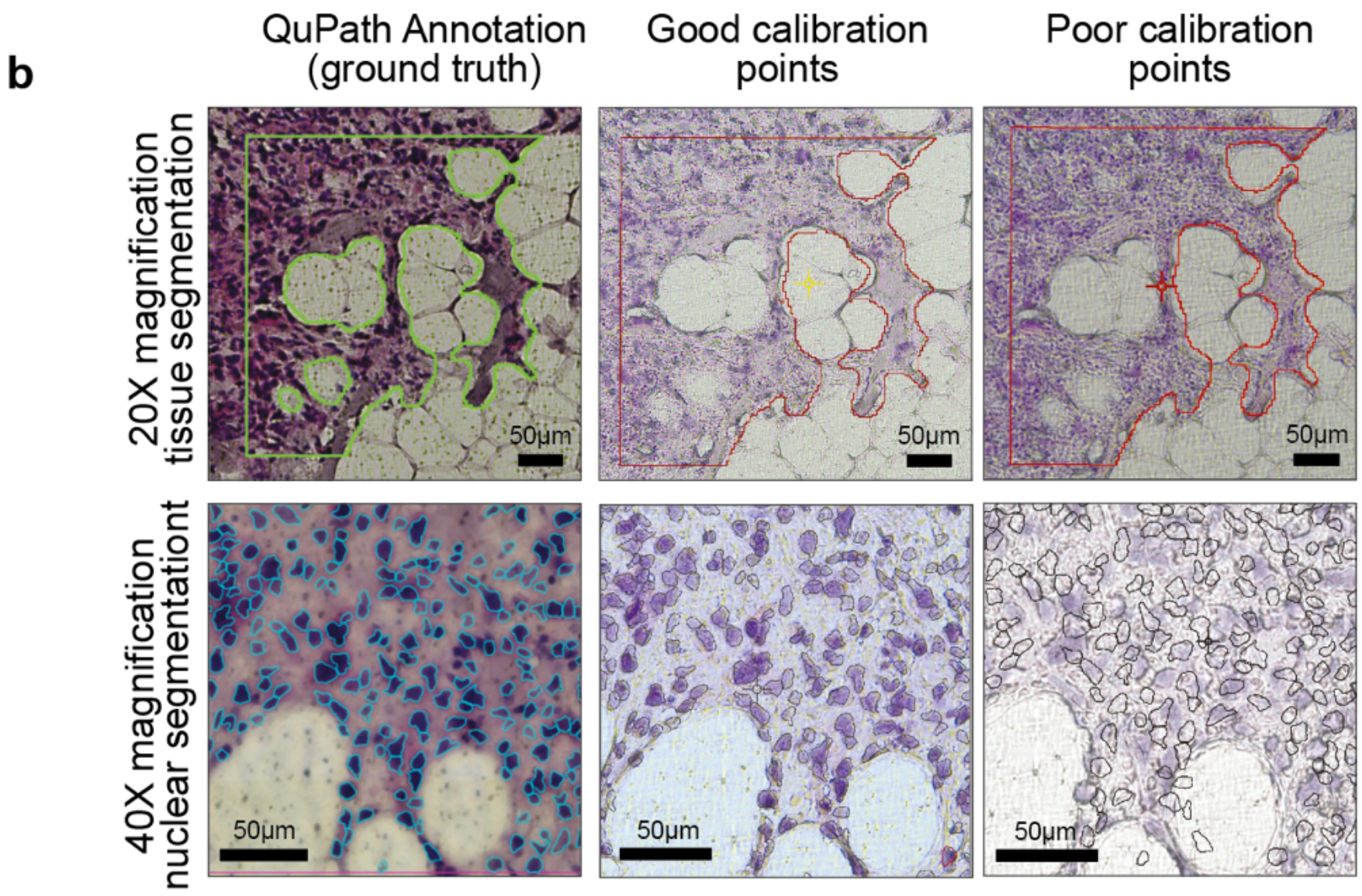

Revision Fig. 6: Accurate reference point selection enables reliable contour transfer for laser microdissection. (a) Tissue overview and reference point definition in QuPath. G1–G3 indicate three good calibration points, located close to the objects of interest and easily identifiable. B1–B3 indicate three poor calibration points, which are located farther away and are difficult to recognize. The center shows four annotated regions: two magenta boxes corresponding to regions subjected to nuclear (H&E-based) segmentation in QuPath, and two irregular regions corresponding to tissue segmentation annotations. (b) Accurate annotation transfer depends on reliable calibration points. Top row: large tissue segmentation annotation and its contour transfer to the LMD7. Poor calibration points result in an upward shift of the entire contour. Bottom row: nuclear segmentation and contour transfer. In this case, poor calibration points lead to relatively large positional errors, which would result in improper single-cell collection.

- Cell size varies substantially based on cell type, cell-cycle stage (G1/S/G2), and pathologic state (e.g., cancer vs. normal), which is reflected in the nucleus-to-cytoplasm (N/C) ratio. Since the proposed study uses segmentation based on nuclear identification, can the authors clarify how the cytoplasmic area was determined, and how the model handles cells with different N/C ratios, for example, epithelioid vs. fusiform cells or lymphocytes versus lung carcinoma cells?

We agree that cytoplasmatic area and other morphological features are also relevant, and should be measured when dealing with more cell types beyond the eight imaged markers included in our proof-of-concept data. We relied on nuclear segmentation and expansion as a consistent approach for all cell types, independent of the mIF markers. We did not determine the cytoplasmic area of cells. Importantly, openDVP with its modular nature supports such analyses with the latest Mesmer and Cellpose algorithms for nuclear and cytoplasmic segmentation.

- Between lines 410–421 and in Fig. 5i–j, the authors claim that the DCIS proteome of the primary tumor is more heterogeneous than the relapse sample, citing a lower global proteome correlation (mean Pearson r = 0.91 vs. 0.95; Fig. 5i), higher protein-level variability, and higher spatial autocorrelation (Moran's I; Fig. 5j). However, I think the current data do not entirely support the idea of heterogeneity. Both Pearson r values remain high, and the p-value could reflect technical factors (e.g., mass spectrometry or sample-processing variation) rather than true biological differences. Moreover, Moran's I results are reported without p-values, making it difficult to assess statistical robustness. In that regard, single-cell measurements would help get a cleaner read on intra-tumor heterogeneity, especially since the method can potentially be applied for single-cell resolution.

We thank the Reviewer for this interesting point. Our new data in Fig.5 allowed us to also assess single-shape proteomic differences in both TNBC tissue samples (primary and relapse). Despite the expected much lower proteome coverage obtained from single-contour measurements (Rev. Fig.7b, below), we observed a similar trend for all three DVP approaches (classic DVP, neighborhood-based DVP and single-cell DVP). The relapse sample featured significantly higher proteome correlations (Rev.Fig.7c, below).

We have toned down this statement and now write in the main text: “Overall, the DCIS proteome of the primary tumor showed lower global proteome correlation (mean Pearson r = 0.91 versus 0.95, Mann-Whitney-U test p-value: 1.6E-46, Fig. 6i) and higher protein level variability (CV, Extended Data Fig 5d), which was also reflected in the single-shape proteomes despite lower proteomic depth (Extended Data Fig 6e).”

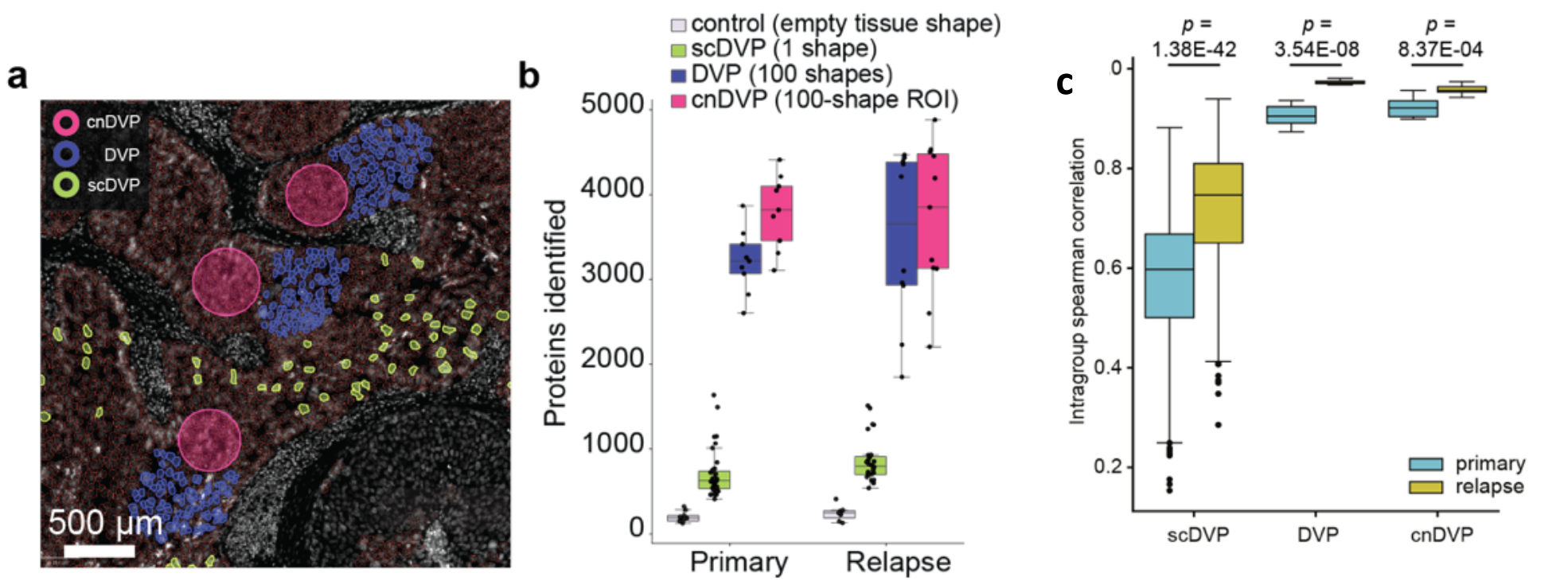

Revision Fig. 7: Three DVP approaches reveal higher intra-tissue proteome heterogeneity of the primary tumor. (a) Images of primary TNBC tissue, showing the nuclear stain with segmentation mask for phenotyped cancer cells with red outlines on the left and CD3+ cells with green outlines on the right. Overlayed are collection annotations for the three DVP approaches. In magenta cnDVP, in blue DVP, and green for scDVP. **(b)** Boxplots with number proteins identified with DIANN search across DVP approaches. **(c)** Boxplots showing intragroup spearman correlations between cancer only samples for the three DVP approaches scDVP, DVP, and cnDVP. Protein groups are shown with at least 70% valid values for each

sample group (primary or relapse) and approach (DVP, scDVP and cnDVP). P-values were calculated with a two-sided independent t-test.

We also provide the p-value (<0.001) for Morans I's Mann-Whitney test in the revised Figure 6 legend.

- The biological examples are compelling and well chosen (I particularly like the combination of flashDVP with placenta and the reasoning). However, the examples are much underpowered and lack appropriate controls. This should be included in the discussion as a shortcoming of the examples. The biological interpretation could take up less space in the manuscript, at the benefit of properly benchmarking the workflow parts as described above.

We thank the Reviewer for pointing out that our biological use cases were well chosen. We have revised and shortened the biological discussions throughout the manuscript as good as possible and included a new benchmarking figure (Fig. 5), as suggested.

Minor comments:
- In Fig. 4j, please report the statistical differences between the box plots.

This is now provided in the figure legend.

- In lines 401–402, the PC1 and PC2 variance values don't match those shown in Fig. 5e–f; please correct them.

Thanks for bringing this up. They are now corrected.

- In the lung tumor analysis, the authors report 133 FDA-approved drug targets enriched in the EMT niche. Please clarify the criteria used to compile this set, and whether any drug-enrichment/connectivity analysis was applied?

The list of FDA approved drug targets was downloaded from the Human Protein Atlas website. In total, we could map 180 proteins to our dataset. We have revised the main text and figure to better describe our analysis strategy. In the updated volcano plot in Ext. Data Fig. 3e (Figure below), we now only label the 21 significant proteins (FDR < 0.05), of which 16 were significantly higher in EMT niche cancer cells compared to cells outside this niche. This includes the receptor tyrosine kinase PDGFRB. E-cadherin (CDH1) and Vimentin (VIM) are shown additionally for reference.

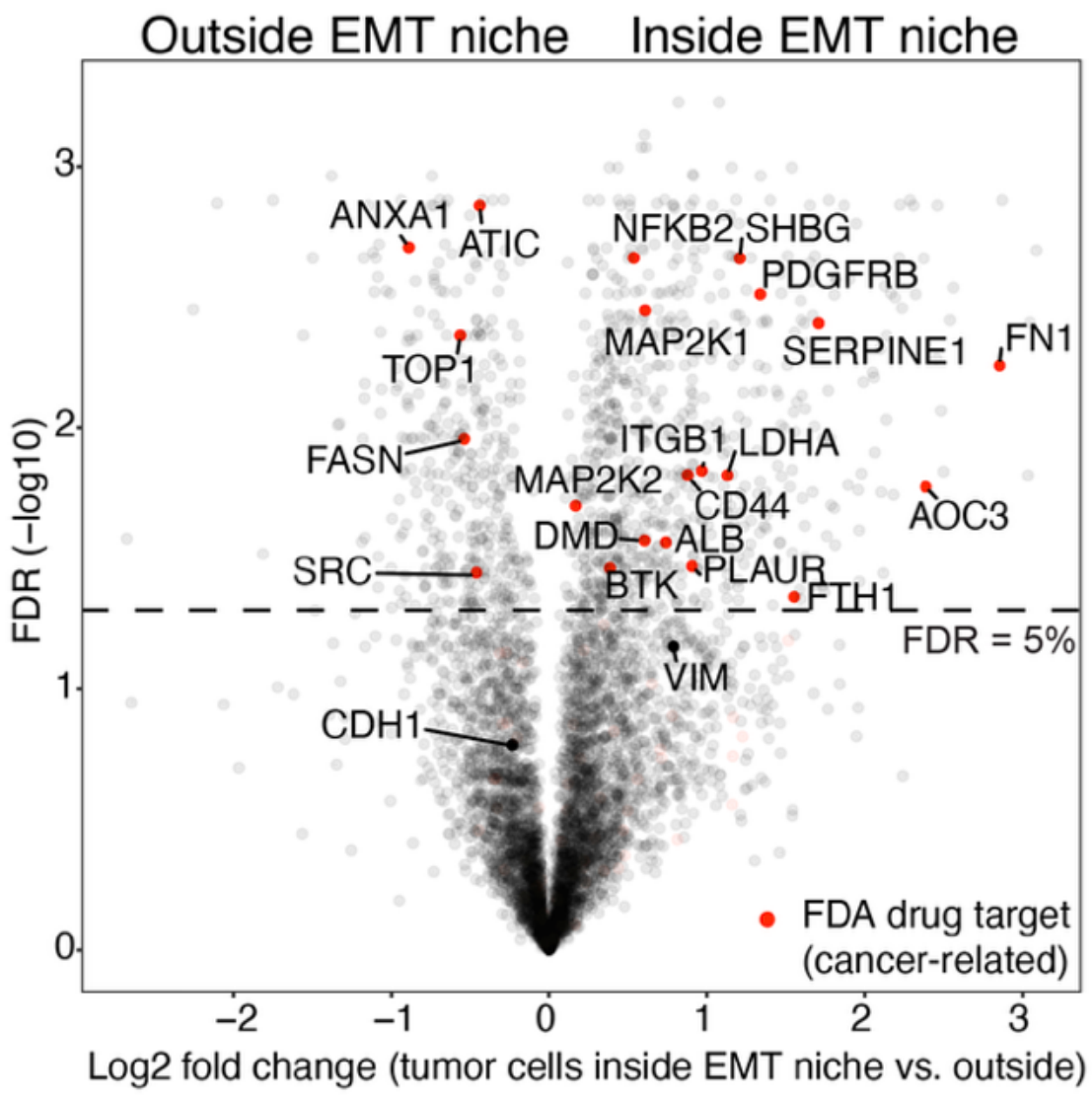

Revision Fig. 7: Volcano plot showing differentially abundant proteins between tumor cells inside and outside the EMT niche as defined by CosMx spatial transcriptomics. FDA-approved drug targets are highlighted in red. E-Cadherin (CDH1) and Vimentin (VIM) are shown for reference.

- Given that N-cadherin is a canonical EMT marker (cadherin switch), please explain why E-cadherin was prioritized.

We focused on the two EMT markers E-cadherin (CDH1) and Vimentin as two experimentally validated antibodies were already available at the time of the experiment. We hence selected those antibodies for our low-plex immunofluorescence antibody panel used to distinguish the different tumor-cell phenotypes for proteomic profiling. As expected, proteomics showed a clear trend towards lower E-cadherin levels in EMT niche tumor cells compared to cells outside of this niche (Ext. Data Fig. 3e). On the contrary, VIM was higher in the EMT niche in agreement with spatial transcriptomics (CosMx) and immunofluorescence imaging. We also checked N-cadherin levels, but this protein was not identified unfortunately. However, CD44, another marker of mesenchymal (EMT-like) tumor cells, showed strong and significant up-regulation in EMT niche tumor cells (Ext. Data Fig. 3e).

- In lines 269–270 the authors describe the EMT niche as panCK+/VIM+/E-cadherin-, but Fig. 3c–d and the legend indicate E-cadherin+. Please reconcile this text–figure discrepancy.

Thanks for pointing this out. We have corrected this to panCK+ and VIM+.

- Please describe the staining protocol applied for lung tissue.

We now provide the staining protocol in the lung cancer methods section.

- Regarding the cell-neighborhood spatial proteomics analysis, please clarify why tumor–immune ROIs (CN5) were not sampled in the primary biopsy. Also, explain the rationale for the imbalance in tumor-enriched ROIs (sample numbers) between the relapse and primary biopsies.

CN5 was not sampled in the primary biopsy because of the lack of large enough regions for cnDVP collection. Additionally, we had a sampling imbalance due to a few dropout samples that did not pass QC filtering.

- The collected samples reported for CN in Fig. 5a (210) does not match the number reported in the text (203); please reconcile this discrepancy.

This discrepancy comes from additional QC filtering. We have clarified this in the figure accordingly.

- Please clarify what the two box plots in Extended Fig. 5a correspond to (primary and relapse biopsy?). This is unclear since tumor–immune regions were not considered for the primary biopsy.

This box plot shows the number of identified proteins and precursors for the analysed cellular neighborhoods. It includes both tissues (primary and relapse). As the Reviewer pointed out, for the tumor-immune CN this relates to the relapse sample. We have changed the colour in the revised version to make clearer that proteins (white boxes) and precursors (turquoise boxes) are plotted together with two y-axes. Note, this figure is now Extended Fig. 6a.

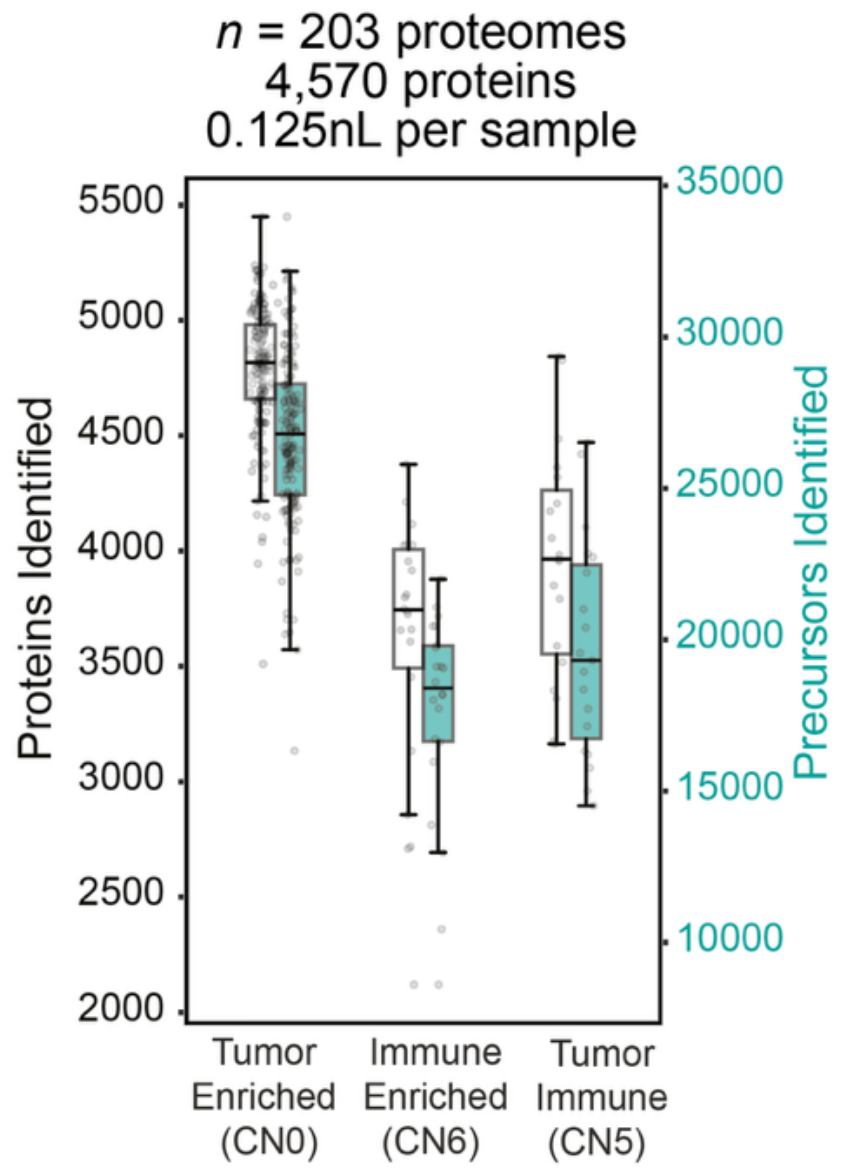

Revision Fig. 8: Boxplot showing proteins and precursors identified per group of cellular neighborhoods.

- Please provide the reference for the data shown in Fig. 5k.

These data are provided in the Extended Data Table 4.

- Figure 1 is confusing at times and needs cleaning. The point of panel e is unclear even with the legend.

Thanks for this suggestion. We have revised the figure and hope it is easier to understand now. We think the example panel in Fig. 1e is still important to contextualize the workflow

Fig. 1a with concrete examples. It is meant to prepare the reader for some of the analyses explained in more detail later in the manuscript (Figs. 4-6).

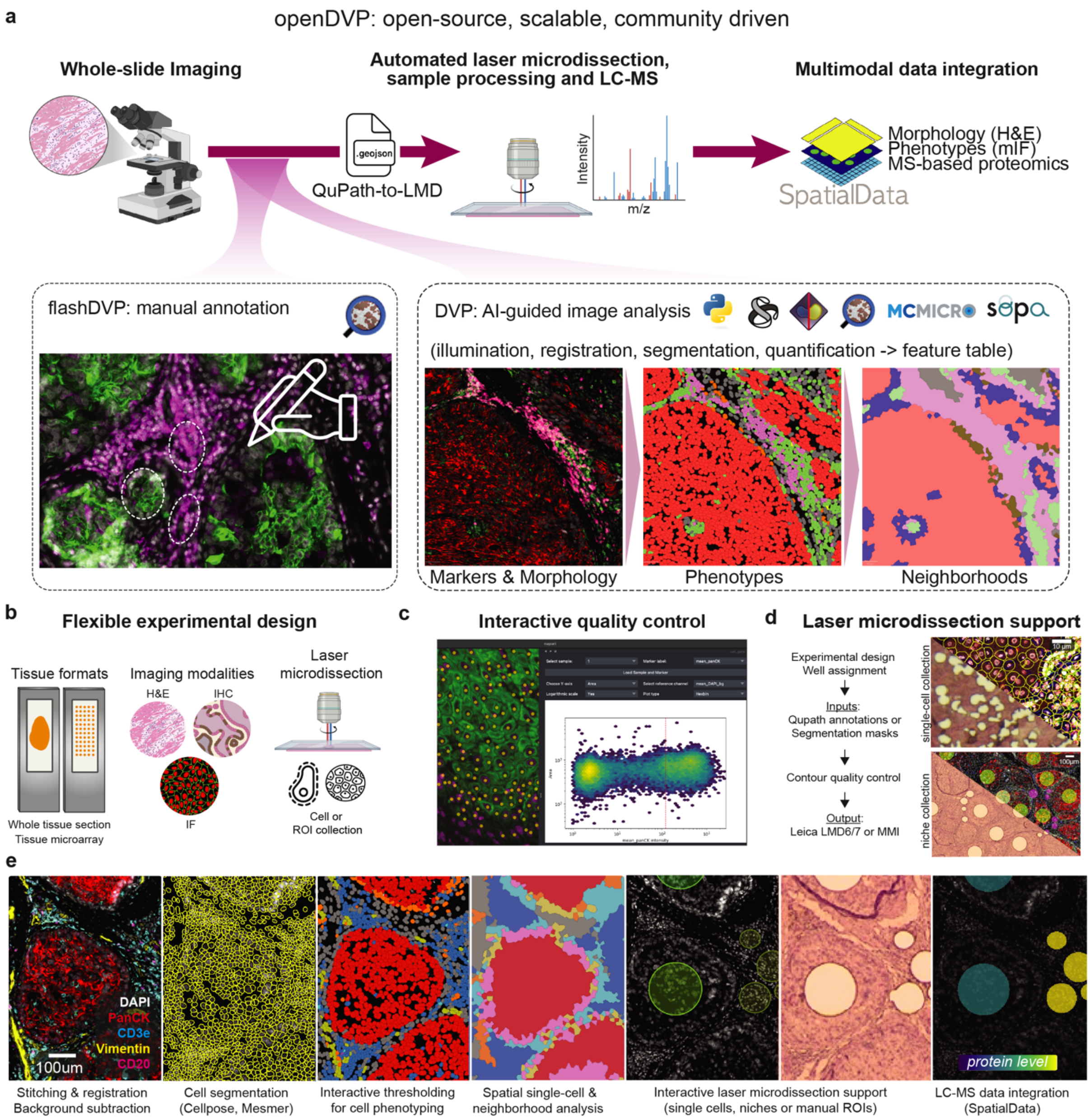

Revision Fig. 8. Overview of the openDVP framework. (a) Main workflow of openDVP begins with tissue imaging to produce TIFF files. FlashDVP comprises manual image annotation, coordinate transfer with the QuPath-to-LMD webapp, and laser microdissection. OpenDVP provides a framework to process images into feature tables and analyze matrices to phenotype cells, using open-source tools. These data layers provide the basis for contour export and laser microdissection via QuPath-to-LMD. **(b)** OpenDVP supports tissue microarrays or whole-slide images, common imaging modalities, and different LMD collection strategies. **(c)** The Napari-cell-gater plugin enables phenotyping through visual feedback thresholding, plotting cell features in FACS-like density plots. **(d)** QuPath-to-LMD webapp allows users to design, transfer, and validate annotations from QuPath and openDVP to an LMD-ready file format. **(e)** Exemplary openDVP workflow showing the results of multiplexed immunofluorescence (mIF) imaging, cell segmentation, cell phenotyping, cellular neighborhood analysis, contour annotations, and multimodal data integration using SpatialData.

- The paper introduces a number of new or at least forefront concepts and avenues for DVP, such as spatial transcriptomics on a consecutive section, multiplexed imaging for CN-based ROI identification. This is almost too much for one single paper, but the authors must be congratulated for the excellent job in combining this. However, I feel at times the innovations are not ready to be claimed yet. Particularly, tissue microarrays are mentioned as an innovation, but no data is shown.

We thank the Reviewer for these very encouraging words. Our goal was to offer as much flexibility as possible to support a wide array of biological applications. Whole-slide sections and tissue microarrays are two standard formats for spatial tissue proteomics that openDVP already supports. The TMA based image analysis workflow only requires an additional de-arraying step, but is otherwise identical to the standard pipeline. We therefore did not show separate data. However, we provide detailed documentation of the TMA workflow on GitHub: https://coscialab.github.io/openDVP. We therefore feel that mentioning the TMA capabilities is important for the reader.

- The discussion lacks comparison against other tools in the field, such as BIAS (Cell Signaling Technologies, Hungary) or scPortrait (develop in Matthias Mann's lab). It is also fair to acknowledge other efforts next to this work. Besides this, the references cited are appropriate.

We have revised the Introduction accordingly to better articulate the main goals behind openDVP and added a paragraph behind the main differences between openDVP and BIAS. We focused this comparison on peer-reviewed software tools. Please note that the scPortrait preprint was not released at the time of our initial submission.

- The Github repository is easy to navigate, and the provided video introduction Qupath-to-LMD is well done.

Thank you very much!

Reviewer #3 (Remarks to the Author):

I co-reviewed this manuscript with one of the reviewers who provided the listed reports. This is part of the Nature Methods initiative to facilitate training in peer review and to provide appropriate recognition for Early Career Researchers who co-review manuscripts.

Reviewer #4 (Remarks to the Author):

Nimo and colleagues are presenting a manuscript to introduce openDVP, an experimental and computational framework for simplifying and democratizing deep visual proteomics (DVP). Built on existing open-source software for image analysis and data analysis they develop additional packages and interfaces to offer two workflows for user guided and AI guided analysis. They then demonstrate the application of openDVP on three highly relevant use cases.
DVP is a relatively new proteomic strategy co-developed by the authors that combines

identification of cells or tissue regions of interest based on multi-parametric IF, IHC or histology images with laser capture microdissection and deep mass spectrometry-based proteome quantification. The technology is extremely powerful and is already showing major breakthroughs yet it is technically challenging and complex to implement. To date it is only being used by a limited number of specialized laboratories and the true transformative potential of the technology will only be reached if it becomes widely accessible and implemented.
This is the gap that Nimo et al address with their contribution. As such the manuscript and openDVP fills and important need, is of interest to a wide audience and has the potential to accelerate implementation and use of DVP enabling many discoveries across biology and biotechnology.
The data and methodology are of high quality and for the most part appropriate statistics has been used (see minor point below). The conclusions drawn from the three exemplary analyses are well reasoned based on the data obtained but would require orthogonal validation which is not provided. However, given that the major focus of the manuscript is the introduction of openDVP this seems acceptable as long as this caveat is clearly pointed out. The manuscript is well written and the code is fully accessible and accompanied by documentation, example datasets and tutorials.

We thank the Reviewer for the positive feedback on our manuscript and for pointing out the high quality of our work.

While the manuscript is addressing a critical gap and has the potential to be of wide interest, I feel that critically important information is missing to achieve this goal (and enable proper in-depth review).
- Details on new packages and functions: Very little information is provided about the individual packages and interfaces that have been developed as part of this work. They are accessible on github but without in depth API reference and code review it is unclear how exactly they function, what novel capabilities they offer, what parameters they use and what their limitations are.

Thanks for bringing up this important point. We have now invested significant time and effort to advance the tutorials, provide an in-depth API documentation site, and guidelines for using the different functions of the openDVP package. Please see our documentation site at: https://coscialab.github.io/openDVP/index.html

- The three example applications should be accompanied by detailed tutorials with step by step instructions that can be followed to exactly reproduce the presented data and figures.

This is indeed one of the main goals behind our openDVP GitHub documentation. We have already started to expand this documentation and continuously work on additional example tutorials including the recording of these practical tutorials. However, such recordings take some time until completed. Additionally, we have also started to teach the openDVP workflow at annual workshops as part of the European Single-Cell Proteomics Conferences in Vienna (first workshop August 2025). Recordings will also be made freely available for the next upcoming meeting.

- It is not immediately clear to the reader how the workflows used to analyze the three exemplary applications differ and what elements of the pipeline they employ. It would be very helpful to provide a schematic for each detailing the data and modules used and stating key parameters where necessary.

We thank the Reviewer for this observation. We agree that distinguishing the specific workflows for each vignette is important for reproducibility and reader comprehension. In the original submission, we organized the Methods section by process (with specific subtitles for each vignette) to highlight how parameters differ at each step. We recognize this might make it harder to visualize the workflow for a single vignette holistically. We have added an introductory paragraph to the Methods section to explicitly guide the reader through this structure, ensuring they can locate the specific pipeline elements for each application without the need for additional schematics that might duplicate the text.

In addition, the following should be considered by the authors:
- The example analyses lack key quality metrics that should be made accessible in the extended figures or tables including data completeness, CVs, intensity distributions, sample clustering before imputation, ...

Please note that that the underlying proteomic data are all provided in the extended data tables or through the Pride submission (unprocessed).

- Fig 2h is complex and most of the visualization elements does not aid understanding a more basic format may be more accessible. It is further unclear how proteins enriched in two groups are colored.

We have revised this figure accordingly. The enriched pathways are now shown as dot plots (figure below). Individual proteins are removed and category sizes ("Count") are summarized by point sizes.

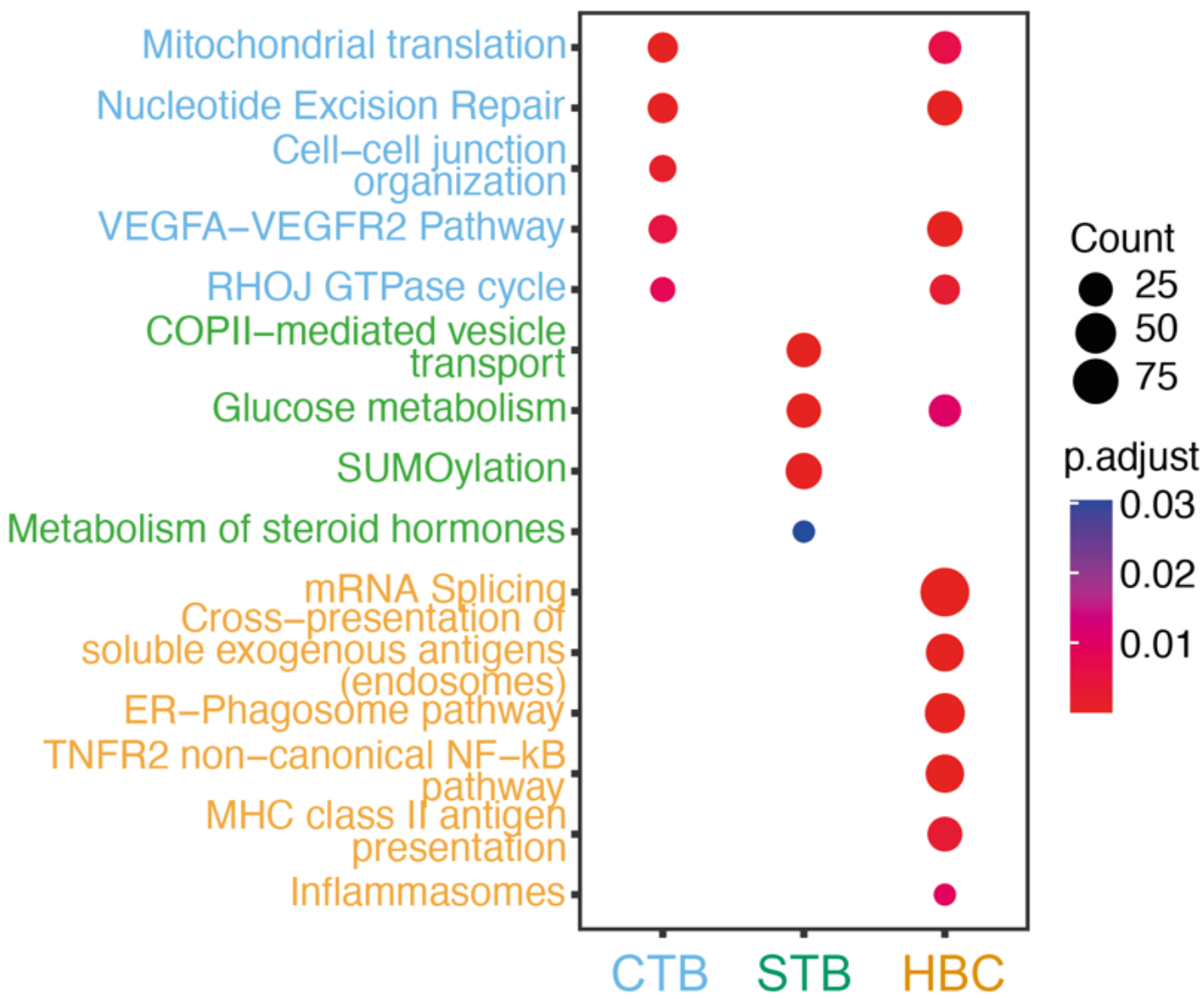

Revision Fig. 9: Pathway enriched analysis of placenta proteomes. Dotplot of enriched functional pathways of phenotype-separating clusters from Fig. 2g. Enrichment based on ranked proteins using the

Reactome database. Color shows adjusted p-values of overrepresented pathways and dot size indicates category sizes ("Count").

- Was the clustering and correlation analysis of the lung cancer data performed before or after imputation? How much is the correlation influenced by the proteins missing in a group and therefore full imputed in this group?

We have revised the figure and legend accordingly. Our revised correlation analysis and unsupervised clustering is without imputation and only reflects the true measured data.

- In the discussion the authors claim "Instead of excising single cells, cellular neighborhood-guided proteome profiling represents a more robust and streamlined approach for phenotype-resolved discovery proteomics." There is no data or reference substantiating this claim. It would excellent if the authors would compare the biological insight gained from analyzing the same are on consecutive sections by single cell proteomics vs neighborhood guided mini-pools.

Thanks for pointing this out. Please also see our detailed responses to Reviewer 2. We now provide an entire new main figure (Fig. 5) and corresponding Extended Data Fig. 5, in which we systematically compare the neighborhood-based approach (cnDVP) to our standard DVP strategy (Mund & Coscia et al, Nat. Biotech, 2022)[1] and single-cell DVP (scDVP), as introduced by Rosenberger et al. (Nature Methods, 2023)[2].

In a nutshell, cnDVP offers deeper proteome coverage and significantly faster tissue collection (>100X faster) than the original DVP approach. Qualitatively and quantitatively, we observed highly similar proteomic profiles for tumor niches and immune (T-lymphocyte) niches. We think cnDVP is particularly powerful for homotypic niches (dominated by a single cell type) or niches of clearly defined cell type composition. scDVP on the contrary offers the highest spatial resolution, but is technically the most challenging approach and limited to larger cell types. We have summarized these comparisons in Fig.5f to help users selecting the right approach for a given spatial proteomics application.

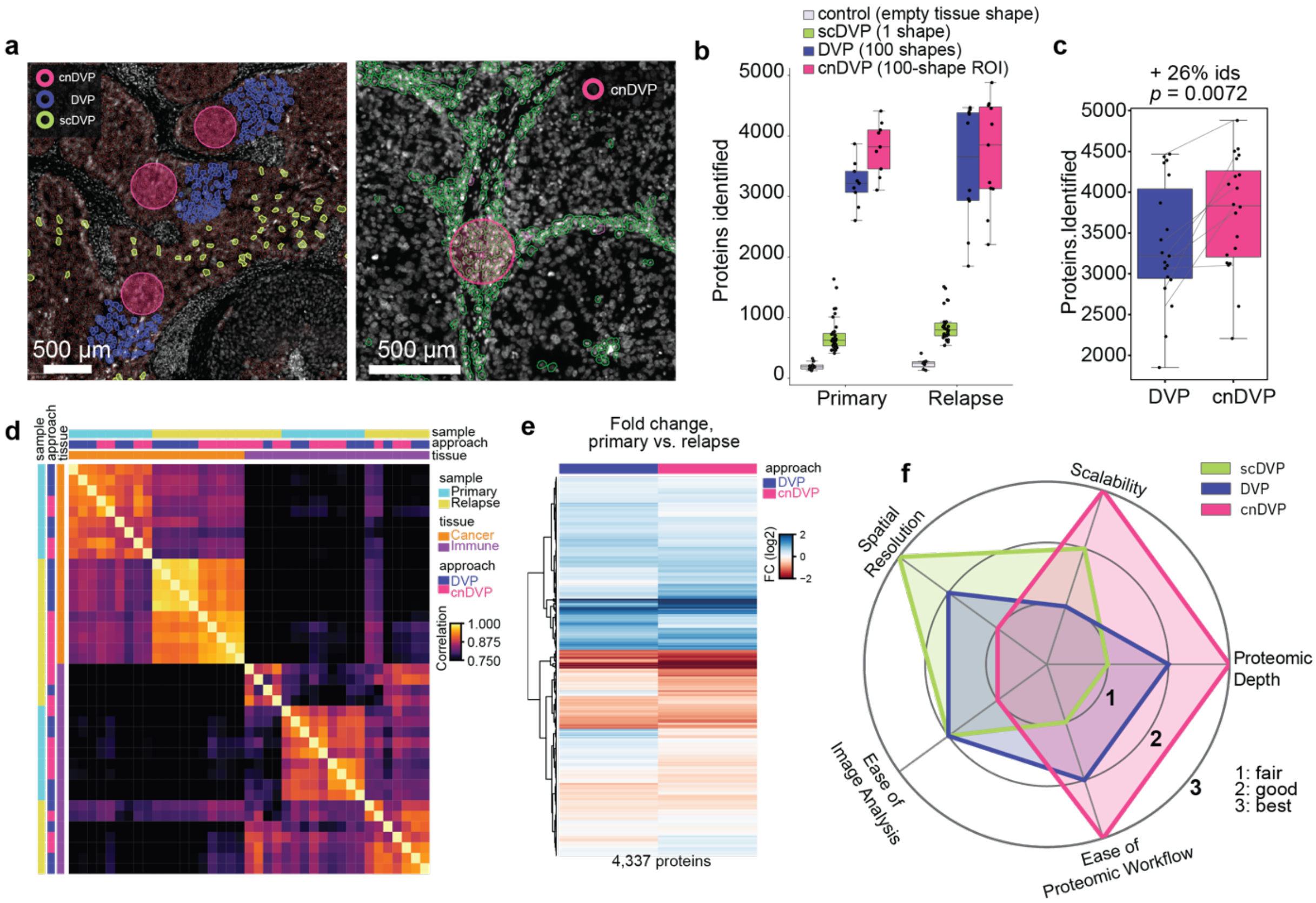

Revision Fig. 10: Cellular neighborhood guided DVP improves proteomic depth and throughput. (a) Images of primary TNBC tissue, showing the nuclear stain with segmentation masks for phenotyped cancer cells with red outlines on the left and CD3+ cells with green outlines on the right. Overlayed are collection annotations for the three DVP approaches. In magenta cnDVP, in blue DVP, and green for scDVP. (b) Boxplots with number proteins identified with DIANN search across DVP approaches. (c) Boxplot with number of proteins identified in matching cancer-specific samples analyzed by DVP and cnDVP. Lines denote spatial co-localization, showing an increase of proteome coverage using the cnDVP approach. P-value is based on a two-sided paired T-test between paired samples. For both b and c, the central lines indicate the median, while the box represents the interquartile range (IQR, 25th to 75th percentiles), whiskers extend to 1.5* IQR of the box (d) Proteome correlation heatmap using Spearman correlations for all immune and cancer samples analyzed with DVP and cnDVP. Metric: "euclidean" and method: "ward" (e) Heatmap of log2FC values for cancer samples between the primary and relapse breast cancer tissues across DVP and cnDVP approaches. Note, similar fold changes were retrieved for both approaches. (f) The radar plot summarizes the key differences between three DVP approaches; scDVP (light green), DVP (blue), and cnDVP (pink); across five key properties: Scalability, Proteomic Depth, Ease of Proteomic Workflow, Ease of Image Analysis, and Spatial Resolution. The radial axes represent a qualitative ranking scale where 1 = "fair", 2 = "good", and 3 = "best".

- The authors should review the author list and author contributions. Some author contributions have likely been missed as several authors are listed solely for contributions that do not constitute intellectual contributions (e.g. resources and funding acquisition) and do not warrant authorship. These two sections need to align.

Thanks for this suggestion. We have carefully reviewed and revised the author contribution statement.

- Considering that the authors developed a new cyclic IF staining protocol it would be important to include an extended figure with the relevant controls (at least for the markers used here). Figure 4b is not sufficient to fully evaluate the quality of this method.

We now provide more details on the cyclic IF protocol in the Methods. We have added a section on "Membrane slide preparation" that describes the slide preparation workflow including Poly-L-Lysin coating to prevent tissue loss during the staining and imaging steps. This is particularly relevant for the harsh cyclic IF workflow. The original protocol was developed by Lin *et al* [6] . To ensure staining specificity, antibodies have been validated in human tonsil FFPE tissue mounted on Superfrost® Plus slides (example images below).

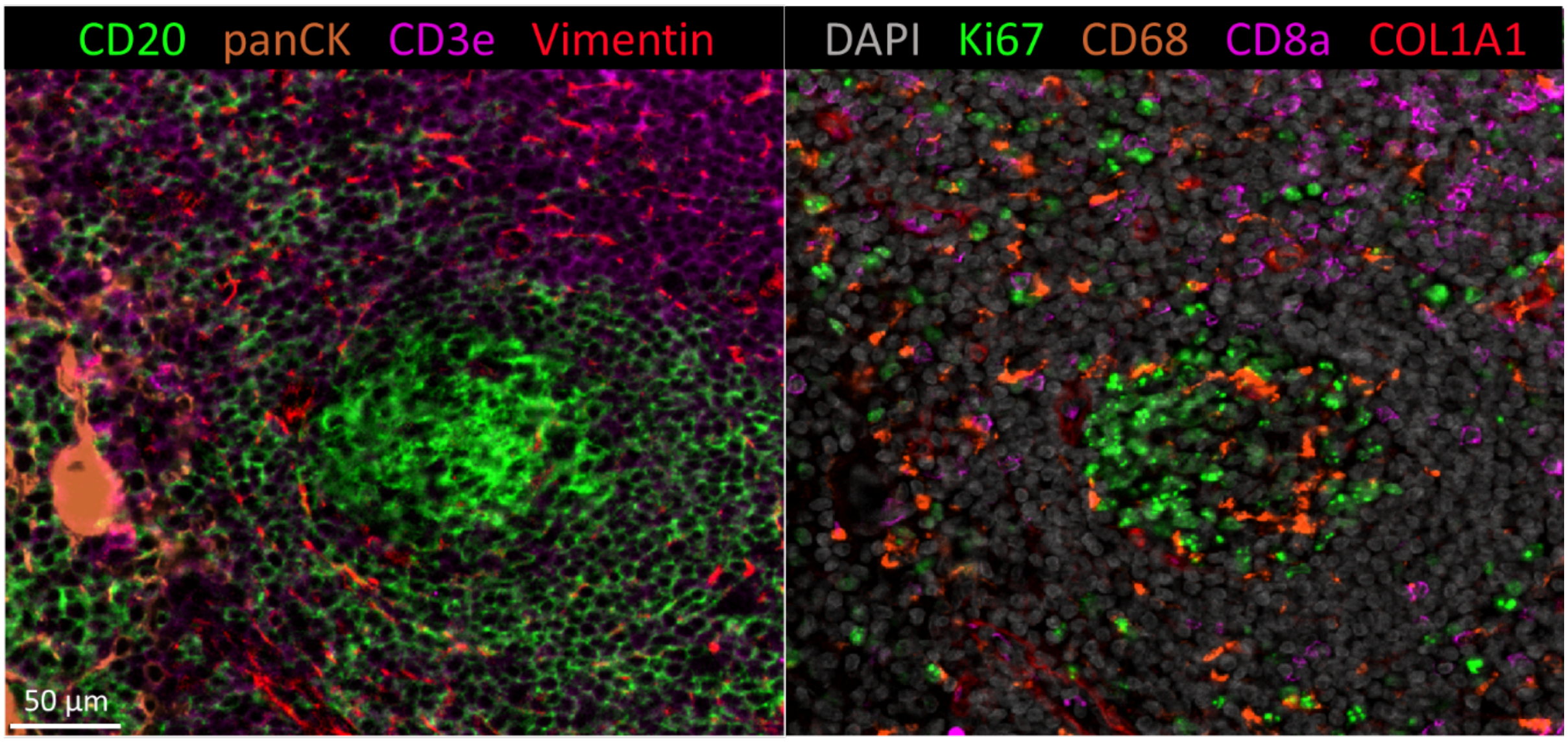

Revision Fig. 11: **Antibody validations for mIF panel**. Validation of antibodies used in breast cancer tissue show on tonsil tissue demonstrating specificity and good signal. These images were obtained using the same imaging workflow as written in the Methods section for the breast cancer tissue.

Minor comments:

- Gene names should be italicized to distinguish them from proteins (e.g. line 286)

This is corrected now.

- Resolution for some figures (eg. extended fig 3g) not adequate

This is addressed now.

- Direct links to the API reference and tutorial should be included at the beginning of the results section for easy accessibility

We added the reference as suggested. Extensive API documentation of functions can be found at: https://coscialab.github.io/openDVP/index.html.

- Outline colors indistinguishable in fig 5o

We have revised the figure legend accordingly to clarify that additionally to the colors of the circle outlines, the circle size also encodes the neighborhood type. Larger circles show tumor-specific niches, whereas smaller circles show immune-specific niches.

- For most heatmaps details on the distance metric and linkage are missing

This is now provided throughout the manuscript.

- Features in plots (e.g. whiskers in box plots) are largely undefined or information not easily accessible

This is now provided throughout the manuscript.

- Fig 3g – the volcano plot for CosMX RNA data appears to use p-values. The authors should consider using corrected q-values or FDR or clearly state why this correction was not necessary.

We have clarified this in the figure and legend. The values shown are Benjamini-Hochberg corrected p-values.

Reviewer #4 (Remarks on code availability):

The code is well structured, readable and well annotated.
I have not tested installation, running and reproducibility of the manuscript data.

We thank the Reviewer for reviewing our GitHub repository and appreciate the positive feedback on our code. In response to Reviewer 2, we have now updated the code to allow smooth installation using different Python versions and operating systems.

References

1. Mund, A. *et al.* Deep Visual Proteomics defines single-cell identity and heterogeneity. *Nature Biotechnology 2022 40:8* 40, 1231–1240 (2022).
2. Rosenberger, F. A. *et al.* Spatial single-cell mass spectrometry defines zonation of the hepatocyte proteome. *Nat Methods* 20, 1530–1536 (2023).
3. Marconato, L. *et al.* SpatialData: an open and universal data framework for spatial omics. *Nature Methods 2024* 1–5 (2024) doi:10.1038/s41592-024-02212-x.
4. Klingeberg, M. *et al.* An ultrasensitive spatial tissue proteomics workflow exceeding 100 proteomes per day. *Molecular & Cellular Proteomics* 0, 101489 (2025).
5. Rosenberger, F. A. *et al.* Deep Visual Proteomics maps proteotoxicity in a genetic liver disease. *Nature 2025 642:8067* 642, 484–491 (2025).
6. Lin, J. R. *et al.* Highly multiplexed immunofluorescence imaging of human tissues and tumors using t-CyCIF and conventional optical microscopes. *Elife* 7, 1–46 (2018).

Detailed point-by-point response

Reviewer #1:
Remarks to the Author:
The key concept of DVP is image-guided automated single-cell segmentation. Critical algorithm components of DVP workflow include whole-slide single cell segmentation (e.g., Cellpose, BIAS), mask file conversion / importation into LMD7 system (e.g., py-LMD), and multi-modal data alignment and visualization. In this study, openDVP aims to develop "an open-source framework providing flexible, interoperable workflows that support simplified experimental strategies for users with limited computational expertise and advanced pipelines optimized for scalability and complex analyses." In this revision, the authors provided detailed responses to my comments. However, the responses are quite general for most of the critical points and didn't make significant improvement to these critical points.
1. Current openDVP framework doesn't provide any novel critical algorithm components, but assemble the existing ones for whole-slide single cell segmentation, mask file conversion / importation into LMD 7 system and down-stream MS data analysis. In this revision, the authors provided "an in-depth API documentation site, and guidelines for using the different functions of the openDVP package." After tested by two researchers with computation science and DVP experience, the package can now be properly installed but is far from a whole integrated workflow for users with limited computational expertise. Current package can only start from exiting well-segmented mask file. The user needs to code additional glue scripts to accomplish the whole task.
To this end, the authors should reorganize their figure presentation to focus on the key novelty of this study for providing a user-friendly pipeline rather than provide comprehensive application without biological validation. As reviewer 4 highlighted, "the three example applications should be accompanied by detailed tutorials with step-by-step instructions that can be followed to exactly reproduce the presented data and figures." Without achieving this user-friendly and integrated workflow from raw whole slide image segmentation, properly aligned mask file into LMD7 system to multi-modal omics data visualization, the novelty of this study is limited. The authors should provide a unified stand-alone graphic user interface to fully reproduce all the three applications.

We thank the Reviewer for the continued feedback on our revised manuscript. We would like to clarify a few things below and respectfully disagree with some of the points raised.

1. Novelty
We would like to reiterate that the motivation behind openDVP what not to develop novel image analysis algorithms (e.g., for cell segmentation), but instead to create the first open-source and extensible DVP ecosystem compatible with many community standards used in the single-cell and spatial omics fields. Advances in computational image analysis, laser microdissection, MS-based proteomics and multimodal data integration have remained highly fragmented efforts. OpenDVP builds on top of these efforts, integrates state-of-the-art methods into one pipeline and also introduces a number of new tools (e.g., QuPath-to-LMD workflow, interactive Napari cell gater) and concepts (neighborhood-guided proteome profiling) to expand the original DVP concept while offering maximal user flexibility.
OpenDVP must not start from segmented mask files, instead it can also start with raw images. When starting with raw images, we suggest to first stitch and register images (where applicable), and segment cells in order to obtain the quantification matrix. All these steps can be orchestrated by the integrated pipelines MCMICRO and SOPA (both suggested), and other ones, such as Harpy, CellProfiler, SPACEc. Based on the quantification matrix, the python analysis package can then take over for single-cell phenotyping.

2. End-to-end workflow and graphic user interface (GUI)
We agree that a GUI will be helpful and this is certainly an important future direction of our work. However, we believe this is beyond the scope of the current manuscript. Notably, many successful and state-of-the-art methods for computational image analysis and spatial profiling do not have separate GUIs. Popular examples include MCMICRO, Scanpy, and DecoupleR. GUIs are generally much harder to maintain.

We did not claim that the full DVP workflow requires only limited computational experience. However, this was the main motivation behind the flashDVP approach (manual cell and ROI annotations) and the development of QuPath-to-LMD as a powerful interface between the highly user-friendly image analysis (and GUI-based) software QuPath and laser microdissection. As we now show in our new Ext. Data Fig 1a., latest generation cell and nuclear segmentation through QuPath's InstanSeg algorithm is on par with the two other supported popular models Cellpose and Mesmer. This now offers openDVP users to segment and classify cells directly in QuPath and subsequently use our QuPath-to-LMD workflow to proceed with laser microdissection and proteomics. This workflow has strong potential to become a "DVP enhancer" for less experienced users.

3. Figure representation and applications
Similar to our original highly cited DVP publication (Mund & Coscia et al, Nat Biotech 2022), we chose to present an overview of the workflow in Fig. 1 and concrete biological use cases in the subsequent figures to illustrate our method's broad applicability. Benchmarking results are included in our new Fig 5 and through a number of additional analyses in the Ext Data Figures (e.g., Ext Data Fig. 1). We think this way readers will best understand openDVP's broad application potential.

2. As my comment and Reviewer 2's comment pointed out "The POI-based approach raised the question of whether the presented methods are still an iteration of Deep Visual Proteomics……", the authors failed to achieve plasma membrane based single cell segmentation, although they have performed surface marker staining such as CD3E. The performance of openDVP for single cell segmentation is therefore questionable.

OpenDVP is capable of leveraging existing whole-cell segmentation approaches, such as Mesmer's whole-cell algorithm, different Cellpose's models, and QuPath's InstanSeg algorithm. To demonstrate this, we have now added an additional side-by-side comparison between the nuclear and cytoplasmic segmentation results of these popular models using the same tissue subset. This breast cancer tissue was mounted on a LMD membrane slide for immunofluorescence staining and imaging (breast cancer example, Figs. 4-6) and subjected to these different algorithms. These new data are now provided in the revised Ext. Data Fig. 1a **(Revision Fig. 1 below).** We also clarified the support of plasma membrane-based segmentation in the revised Fig. 1e and the main text, now stating:

Line 121: *"(2) Modular architecture, integrating latest benchmarked algorithms and machine learning models for cell and nuclear segmentation, such as Cellpose, Mesmer and InstanSeg (Extended Data Fig. 1a)."*

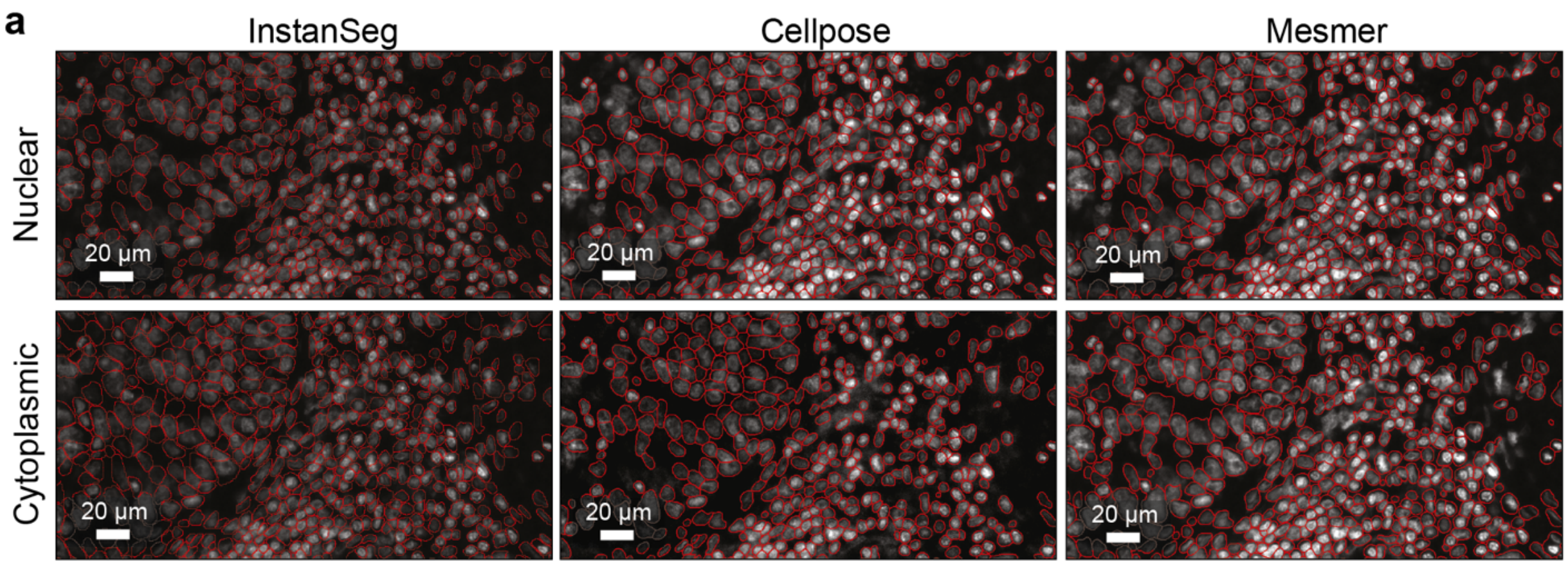

Revision Figure 1: Comparison of three state-of-the-art segmentation approaches (InstanSeg, Cellpose and Mesmer). Shown is a subset of the triple negative breast cancer tissue (primary biopsy), first nuclear stain (Hoechst). Top-row; Nuclear segmentation. Bottom-row; Cytoplasmic segmentation. For InstanSeg's segmentation, the "single_channel_nuclei-0.1.2" model was used on the first nuclear stain for nuclear segmentation. For the cytoplasmic segmentation we used the "fluorescence_nuclei_and_cells-0.1.1" model on all channels, except background images. For Cellpose's (v4.0.9) segmentation, we used the "cpsam" model. For nuclear segmentation we used the first nuclear stain and for cytoplasmic segmentation we used a two-channel (nuclei,membrane) maximum pixel projections across membrane and nuclear markers. For Mesmer, (deepcell v0.12.10), we used the "nuc" compartment for nuclear segmentation, and "whole-cell" for the cytoplasmic segmentation (using the same two-channel segmentation).

Along the line, the authors nicely compared the performance of Cellpose, QuPath and BIAS for nuclei segmentation with acceptable performance. Revision Fig. 2 demonstrated nice whole slide imaging segmentation. However, the authors didn't provide the data for proper whole slide contour transfer for laser microdissection as demonstrated in Revision Fig. 1, raising the question of their performance for whole-slide single cell mask file conversion / importation accuracy. The authors should provide quantitative evaluation as they did for nuclei segmentation.

Whole-slide contour transfer is technically possible, but impractical. The process of exporting single-cell masks as LMD-compatible shapes is computationally efficient, even for thousands of cells. For example, exporting 7,897 single-cell shapes in ".geojson" format and transforming them to the final ".xml" format via our QuPath-to-LMD web-app took under 10s. The current limiting step is loading the shapes into the LMD7 software. We analyzed loading times, showing that loading 8,000 single-cell shapes took us approx. 3-4 minutes (**Revision Figs. 2-3, below**). Based on our estimations, loading one million contours (e.g., all cells of one centimeter-wide 2D tissue section) would take about ~8 hours. Since the LMD software has limited contour import capabilities, we generally recommend to only load cells or ROIs that will be collected during an experimental session.

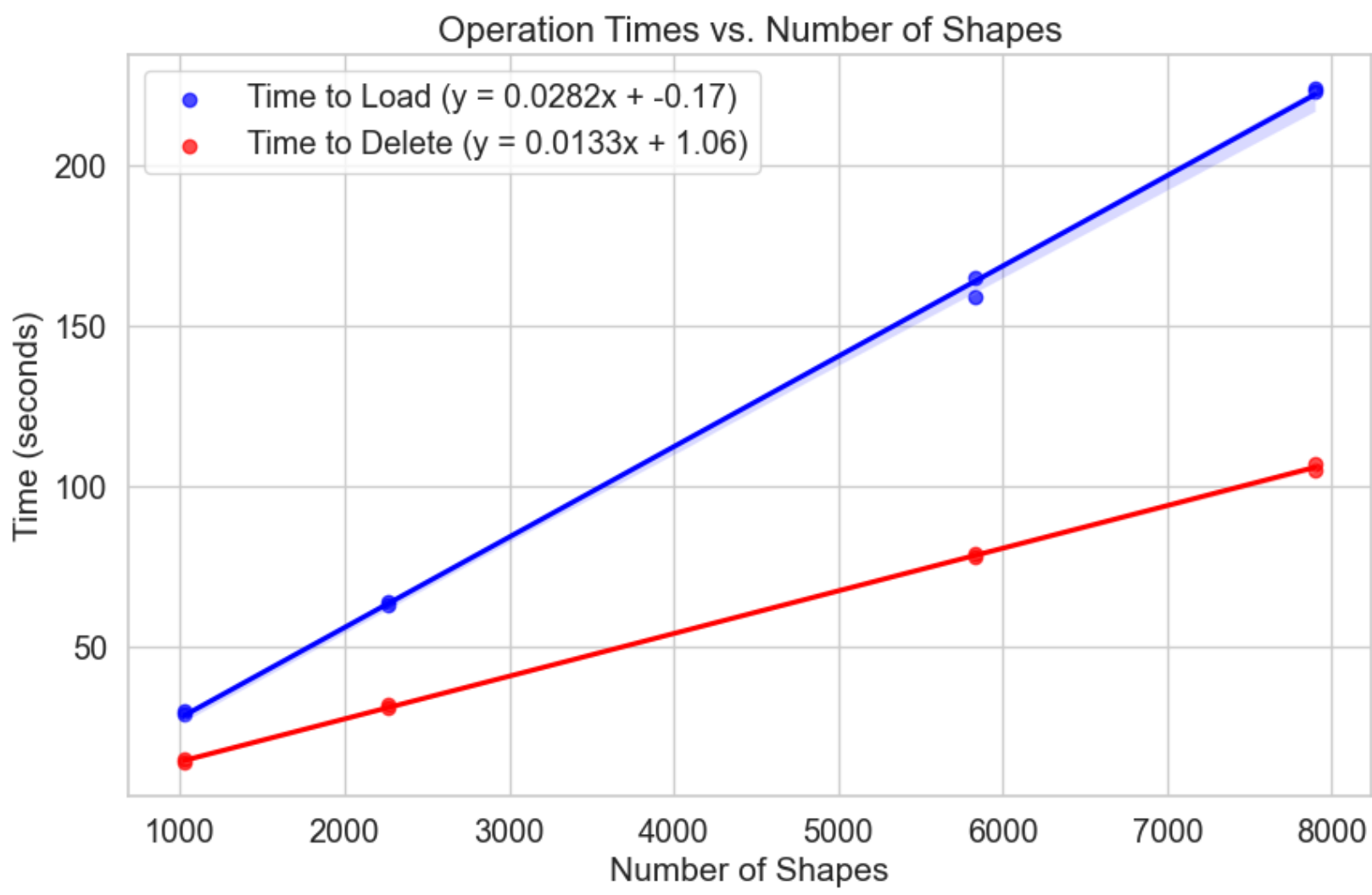

Revision Figure 2: Line graph showing linear relationship between number of shapes (originating from single-cell segmentation) and time it takes to be loaded into the LMD7 software (v8.0.1). Time to delete contours is also displayed.

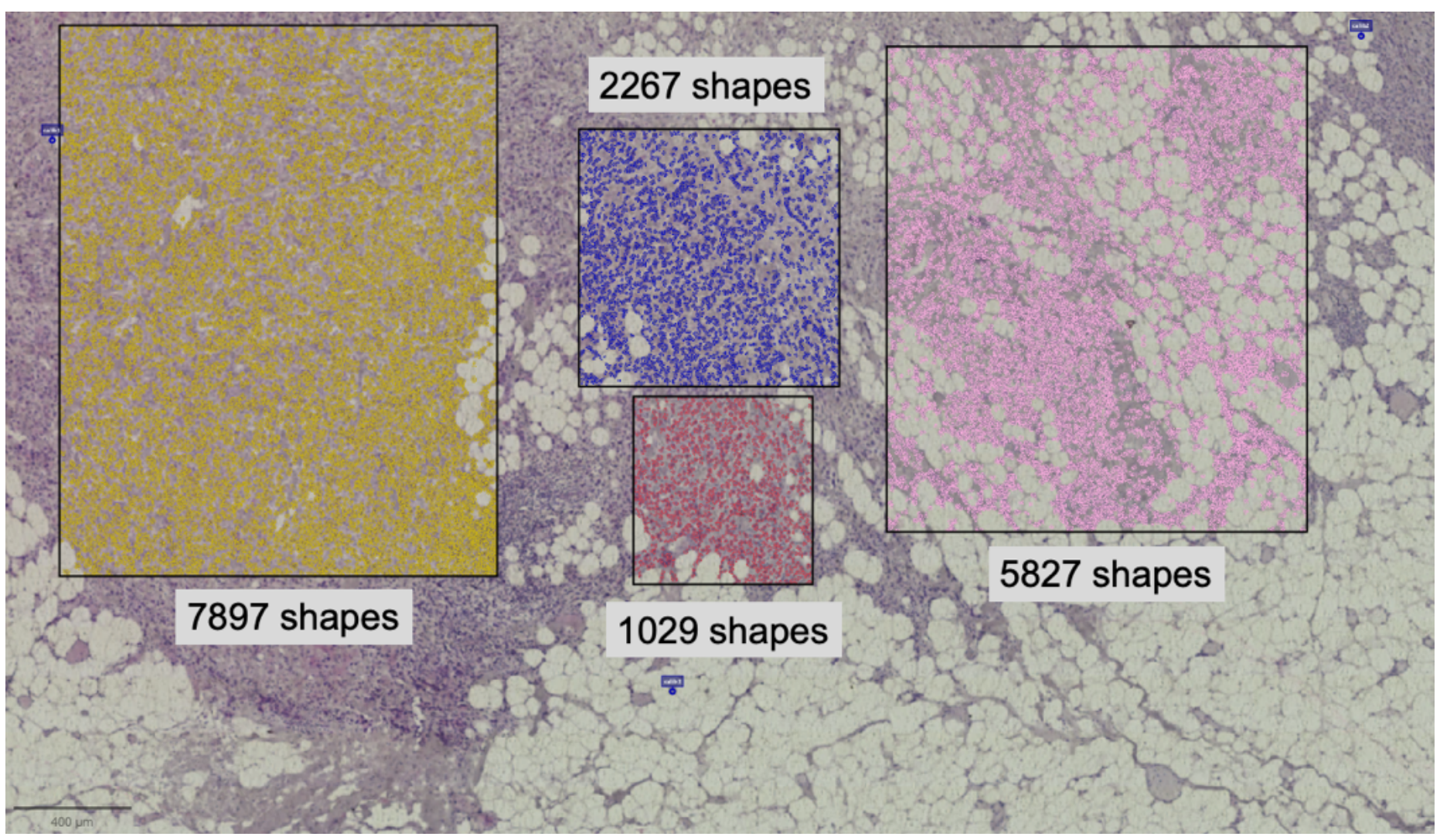

Revision Figure 3: Different number of cell shapes. Segmentation via QuPath's (v7.0.0) Cell Detection module.

3. The cnDVP concept is practically useful but pretty much an imaging-guided mini-bulk spatial proteomic analysis, losing single-cell-type resolution which is the key concept of DVP. Such type of application doesn't need rely on mask file with irregular shape and deviates from the key topic of this study for developing open-source DVP framework. I would suggest the authors to move these nice data to a separated story and expand the figure presentation as highlight in point 1.

We fully agree that single-cell type (and spatial) resolution is the key idea behind DVP. However, achieving single-cell type resolution at the proteomic level does not strictly require single-cell based laser microdissection, which is a general bottleneck in the DVP workflow preventing further scalability. This motivated us to integrate cellular neighborhoods (CN), which also encode cell identity information following cell segmentation and phenotyping. We therefore respectfully disagree that the cnDVP approach inevitably loses cell type resolution as this is strictly dependent on the exact use case. In our proof-of-concept example comparing DVP and cnDVP, we profiled homotypic tumor-cell neighborhoods (tumor cells surrounded by tumor cells of the same phenotype assignment after

segmentation). Our proteomic data clearly show that in such cases cutting 100 single-cell shapes (DVP) is not necessary and that one single contour of 100 phenotype-matched cells guided by cellular neighborhood analysis provided quantitatively highly similar data with consistent quantitative differences between the primary and recurrent tumor cells. However, the cnDVP based sample collection is significantly more scalable (for 100 single-cell shapes approx.100x faster), yielded less dropouts in the gravity-based collection system (Leica LMD7), and improved proteome coverage by 26% on average. As also explained in our response to Reviewer 2, we attribute the improved proteome coverage of cnDVP mostly to approx. 10-20% dropouts we generally observe when collecting single cells using the standard DVP workflow. Larger contours are generally easier to collect and visualize during well inspection. We also noted a slight but significant gain in membrane proteins, which likely come from less laser-induced damage of cnDVP generated samples.

Notably, well inspection of a 100-cell contour is a much simpler process as the contour is large enough to be easily spotted during well inspection. Therefore, it offers an important quality control step in the sample processing pipeline to ensure tissue collection was indeed successful and comparable across samples. In contrast, it is currently impossible to determine the exact number of successfully collected single-cell shapes when for example pooling 100 single cell shapes in the same well. Single cells are often too small, more easily adhere to the well periphery, or locate on top of each other, ultimately preventing accurate well inspection to assess collection efficiency.

We agree that in the current version of cnDVP, single-cell mask files are not strictly needed once cellular neighborhood identity is determined. However, as shown below (**Revision Figs. 4 and 5**), our extension of the cnDVP concept already includes automatically merging single-cell segmentation masks belonging to the same neighborhood group. This smart sampling approach ensures that neighborhood mask files for LMD are automatically generated. This strategy can be further customized, for example by defining the total area and distance between ROIs. We plan to release this extension with the next openDVP update. At the moment, users can already overlay cellular neighborhood information with raw images in QuPath and annotate small neighborhood-guided ROI masks. This workflow also offers a simple way to normalize tissue input amounts.

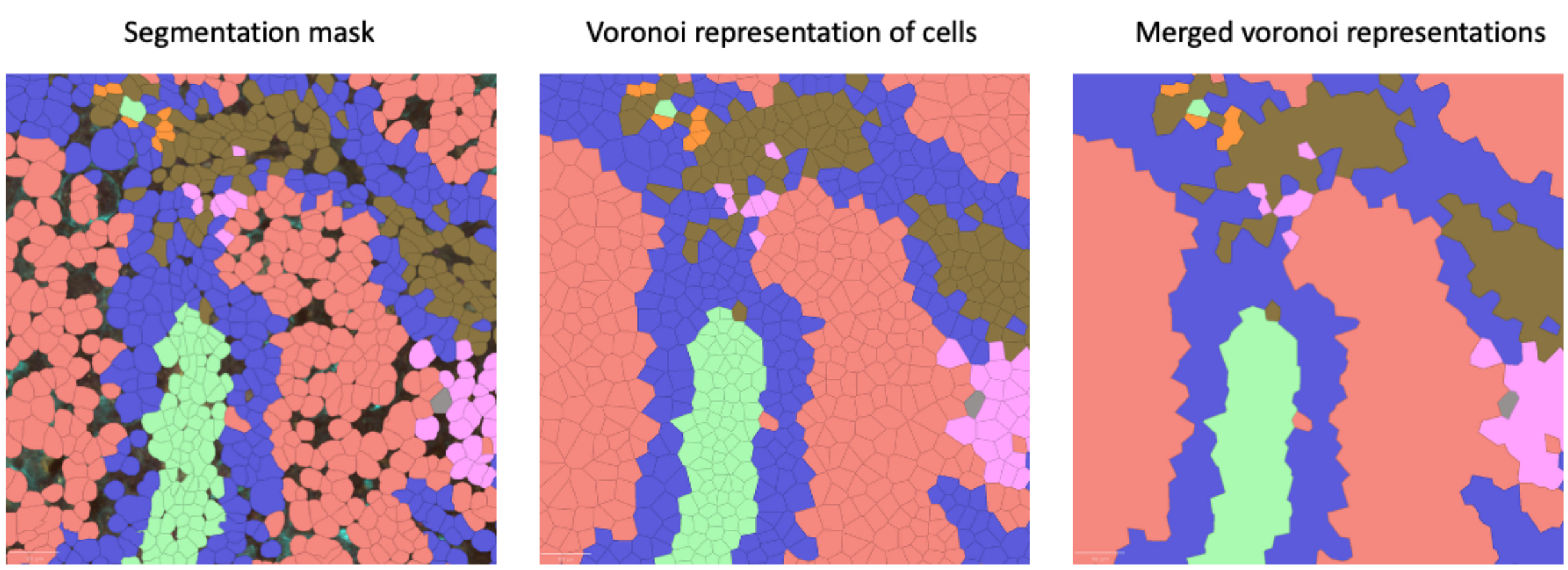

Revision Figure *4:* Workflow to create cellular neighborhood mask file from labelled segmentation masks. Cell segmentation masks provide x,y coordinates for each cell and from which a Voronoi projection is made. Adjacent cell projections of the same cellular neighborhood are merged.

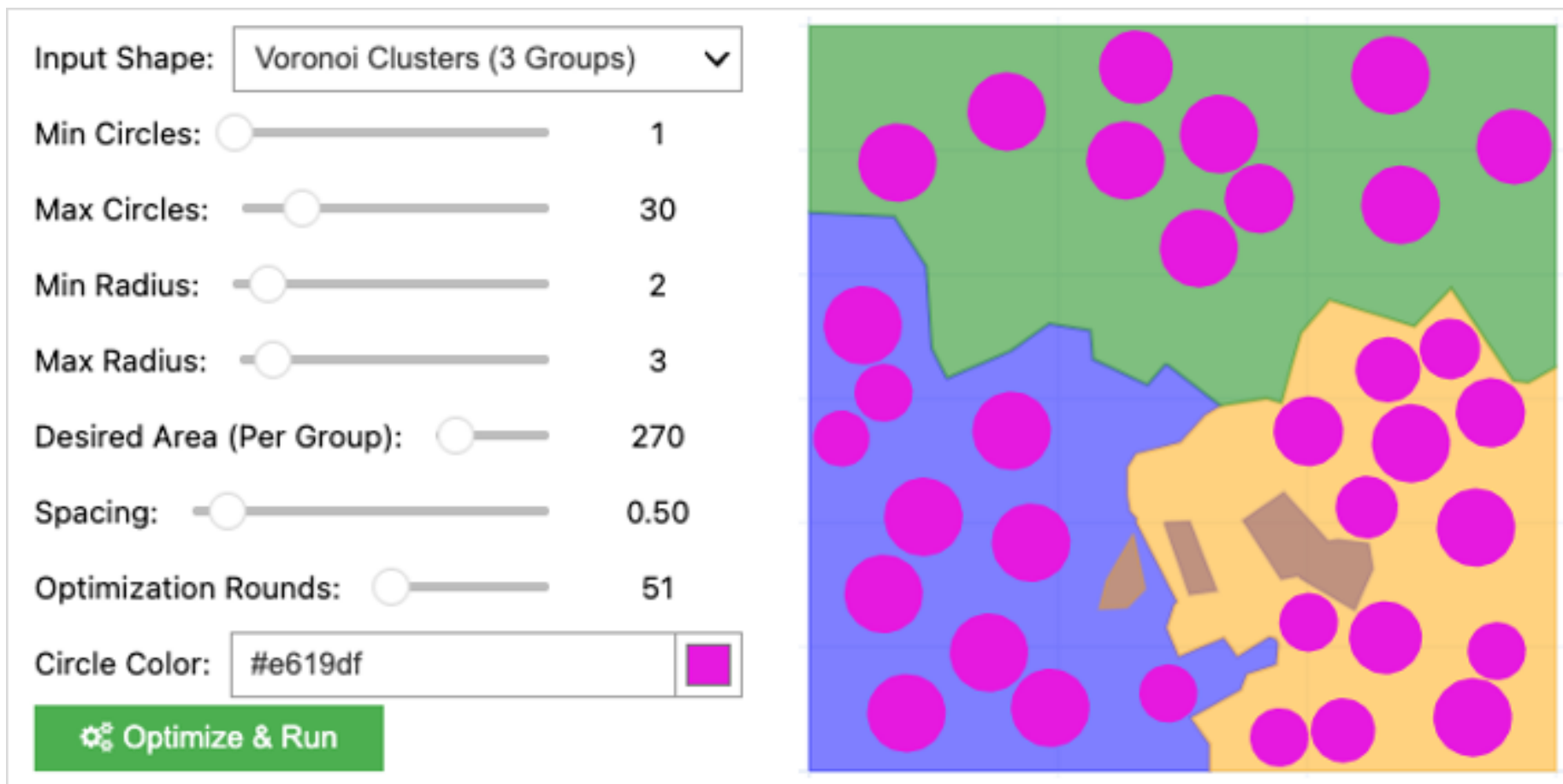

Revision Figure 5*: Concept for automated contour creation from cellular neighborhoods.* First version of the cnDVP extension for automated mask file creation. LMD-ready contours are automatically created based on cellular neighborhood information. The tool allows to customize several parameters, including contour size to normalize sample input amounts between sample groups.

Importantly, and as also highlighted in Fig. 5 and the discussion, we see the cnDVP strategy as an addition (and not replacement) of the original DVP concept that could find broad applicability in highly structured tissue types, such as tumor ecosystems. As biology is complex, we offer high user flexibility in choosing the right tissue sampling strategy depending on the user's needs (scDVP, DVP or cnDVP).

Reviewer #1 (Remarks on code availability):

Included

Reviewer #2 (Remarks to the Author):

The revised manuscript shows substantial improvement and addresses the majority of the reviewers' concerns, thank you! The addition of systematic comparisons between DVP approaches (DVP, scDVP, and cnDVP) in new Figure 5 and Ext. Data Fig. 5 particularly strengthens the work. The manuscript is now even more than before an important contribution to the spatial proteomics field by providing an open-source ("democratic"), community-driven framework for DVP. The systematic comparison of DVP approaches is valuable and will help users select appropriate methods. A few minor clarifications are needed in my view before publication.

Thank you very much!

(1) The novel point that cnDVP "improves" proteome coverage compared to standard DVP should be reconsidered in my view. Given equivalent tissue input (100 cells), there is no clear mechanistic basis for increased protein identifications with cnDVP. If the observed difference reflects improved collection efficiency for single large shapes versus many small shapes (as noted in the authors' response regarding electrostatic losses), this should be stated explicitly in the manuscript.

We mostly attribute the improved proteome coverage to more efficient sample collection. Well inspection of a 100-cell contour is also a much simpler process as the contour is large enough to be

easily spotted during well inspection. Therefore, it offers an important quality control step in the sample processing pipeline to ensure tissue collection was indeed successful and comparable across samples. For the standard DVP workflow, it is currently impossible to determine the exact number of successfully collected single-cell shapes when for example pooling 100 single cell shapes in the same well. Single cells are often too small, more easily adhere to the well periphery, or locate on top of each other, ultimately preventing accurate well inspection to assess collection efficiency. In a classic DVP experiment, approx. 10-20% dropouts can be estimated from single-cell collection.
Based on our new results (also explained in more detail below, points 2 and 3), we have clarified this in the discussion and now state:

"Compared with pooled single-cell contours, cnDVP improved proteome coverage, likely through more efficient sample recovery and improved visual quality control of tissue collection before mass spectrometry."

(2) I am critical that the "improved" proteome coverage by cnDVP is in fact a desirable experimental property. An explanation could be that the ROI-based approach in cnDVP merges signals from different cell types and extracellular matrix, which would inevitably lead to higher protein IDs. However, these come at the expense of dilution of the biological signal. Thus, I think the claim of improved proteome coverage needs to be tempered, omitted or very well discussed at the moment it is stated in the manuscript.
(3) Related to that, did the authors investigate which proteins were uniquely identified by cnDVP (Extended Data Fig. 5b)? Specifically, what is the subcellular localization of these proteins? Did the authors observe any enrichment of extracellular matrix components that could be attributed to the ROI-based nature of cnDVP, or any effects on membrane proteins that could relate to differential laser microdissection damage between approaches?

Thank you for bringing up these important points. As suggested, we additionally performed GO-term enrichment analysis on the proteins uniquely identified in cnDVP samples of both tissues as an extension of our previous Fig. 5 analysis. For the primary and relapse tumor, we identified 504 and 226 cnDVP unique proteins, respectively. The figure below (**Revision Fig. 6**) summarizes the most significantly overrepresented pathways in these subsets. As suspected by the Reviewer, we indeed found that these unique proteins were enriched for the GOCC terms "extracellular matrix part" and "plasma membrane part". The additional membrane proteins could possibly reflect reduced laser induced damage through cnDVP compared to DVP. However, by number, these additionally identified ECM and plasma membrane proteins contribute very little to total number of unique proteins with only 17 related to "extracellular matrix part" and 44 to "plasma membrane" out of the 226 unique proteins in the recurrent sample. For both comparisons (primary and recurrent tissue), we estimated that 77% and 83% of cnDVP unique proteins were not derived from the extracellular matrix. Hence, we conclude that these additional proteins mostly come from tumor cells directly. Our image data clearly show that these ROIs did not include any other cell types than tumor cells. The slightly improved membrane protein identification is an interesting observation, which we have mentioned in the main text. The data are also provided in the revised Ext. Data Fig. 5c. We have revised this statement in the discussion as follows:

"Compared with pooled single-cell contours, cnDVP improved proteome coverage, likely through more efficient sample recovery and improved visual quality control of tissue collection before mass spectrometry."

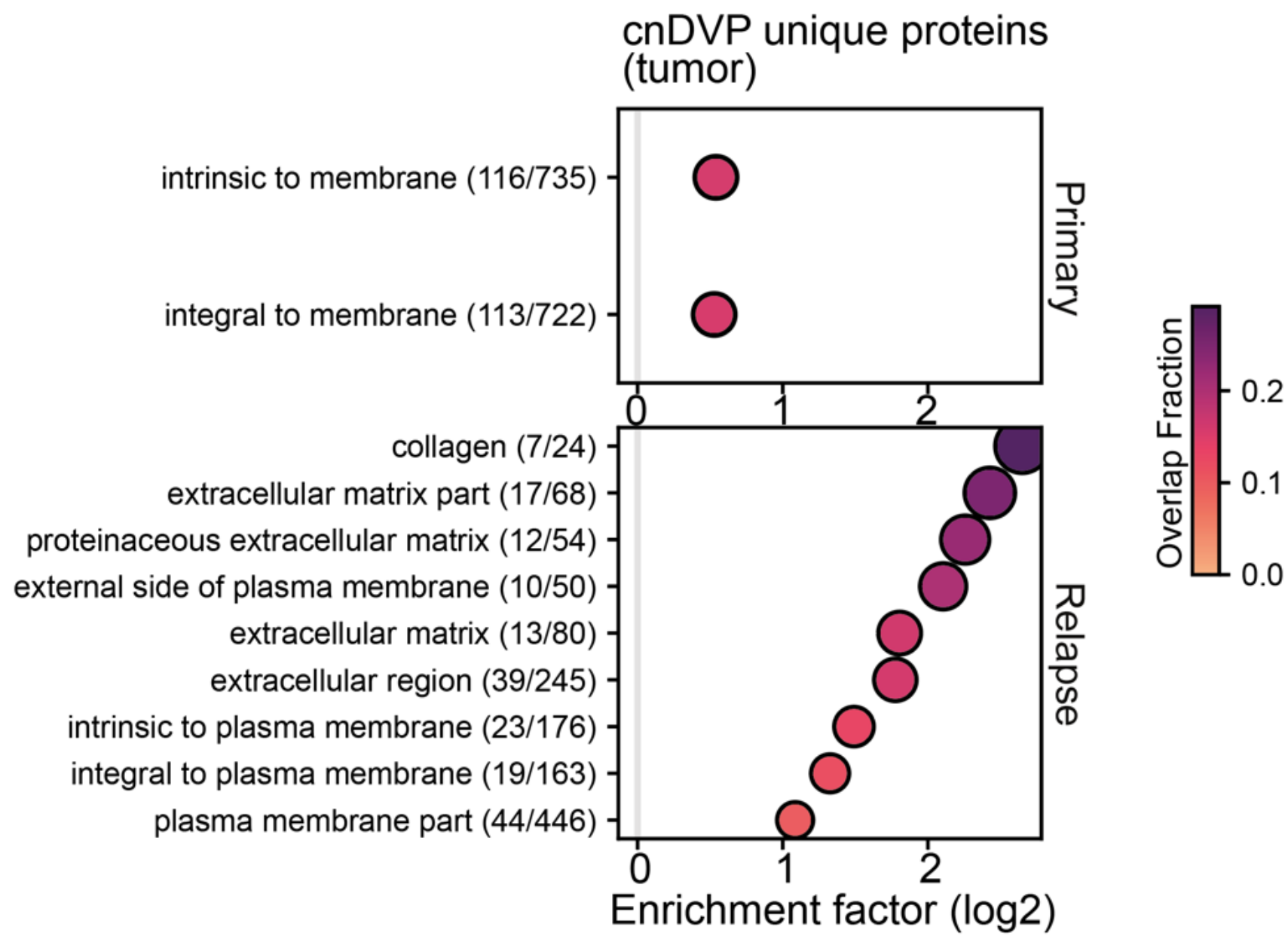

Revision Figure 6: Pathway enrichment analysis (GOCC) of cnDVP unique proteins. Dot plots represent the most significantly enriched Gene Ontology Cellular compartment (GOCC) terms for cnDVP specific proteins. Primary tissue (upper panel) and relapse tissue (lower panel). Unique proteins were determined by comparing missingness between DVP and cnDVP samples of directly adjacent cells with 504 unique proteins for the primary tumor analysis and 226 for the recurrent tumor. Proteins with 40% or more missing values per group (DVP or cnDVP) were filtered out. A Fisher's exact test was used with a Benjamini-Hochberg FDR cut-off of 0.01. The y-axis displays the most enriched terms. Color code and size of dots show the fraction of intersecting proteins relative to the total proteins in the pathway for direct contextual reference.

(4) Related to the comparison of DVP approaches, did the authors track the sample dropout rate for cnDVP? This is relevant because the failure modes differ fundamentally between approaches. In standard DVP, if a fraction of the 100 individual shapes fails to collect (for instance, 90%), the pooled sample remains usable with marginally reduced input (and less protein IDs). In cnDVP, however, failure to collect the single ROI results in complete sample loss. The manuscript states that 203 samples remained "after QC filtering" but I could not find how many were initially attempted. Transparency about dropout rates would help users assess the practical trade-offs, particularly given that cnDVP is rated as "best" for "Ease of Proteomic Workflow" in the radar plot (Fig. 5f).

Thanks again for bringing up this important point. Please also see our previous publication, in which we address dropout rates for ROI-based sampling more systematically (PMID: 41418981, Fig 3B). In that study, we describe the advantage of an additional organic solvent wash to improve our collection efficiency to over 95%.

In our experience, accurately assessing dropout rates from single-cell cuts is practically impossible as single cells cannot be reliably counted within the 384-well after collection. As also outlined above, this has several reasons, such as the size of single cells, their property to easily adhere to the well periphery, or when cells locate on top of each other. All these scenarios can lead to variability in the final proteomics results. Indeed, if 90 out of 100 cells are still collected (10% dropout) this would still result in good quality proteome measurements. However, things are slightly more complicated when fewer total cells are collected (for example due to rare phenotypes), closer to the limit of detection of the LC-MS setup. In such cases, signal losses can more severely impact proteomic depth and quantitative robustness.

We think that evaluating whether a tissue was collected or not, is a rather important feature. The ROI based sampling approach comes with the benefit that a 50-cell or 100-cell contour is generally easy to detect and visualize during well inspection. This offers an excellent binary quality control step in our workflow (collection success yes/no).

Collection efficiency generally varies due to sample related factors, such as tissue-type, storage duration, and treatment protocol; as well as extrinsic factors such as membrane-type, temperature, moisture, and even static charge. Even when controlling for these extrinsic factors, we still see variation ranging between 70% to 95% collection efficiency. For example, for the tumor-enriched cellular neighborhoods of our TNBC tissues, 74% of collected samples could be visually confirmed during well check. Samples that failed this initial collection QC check can then be re-sampled with minimal effort. Secondly, even when tissue regions are visually confirmed, there can also be some dropouts from LC-MS analysis. However, these issues are much rarer in comparison, from 210 measured samples from the three cellular neighborhoods of interest, only 7 had to be removed at the QC filtering step, only about 2% of samples. We have clarified this in the revised text:

Line 349:
"210 regions of interest were measured across tissue sections to capture spatial heterogeneity (Fig. 6b), and processed within one week using an advanced throughput (80 samples per day) DIA-based workflow. After QC filtering, 203 high-quality proteomes remained, quantifying ~4,800 proteins in tumor-specific CNs and 3,500–4,000 proteins in immune-enriched niches (Extended Data Fig. 6a-b)."

(5) The term "cellular neighbourhood" derives from computational spatial analysis tools such as graph-based spatial networks and describes precise node-edge networks between cells as atomic units. This is not how the authors use it here, but here the authors rather refer to something like a niche (EMT niche, tumor niche, immune niche). For consistency and to better convey the intended biological interpretation, consider replacing "cellular neighborhood" with "cellular niche" or "spatial niche" throughout.

We thank the Reviewer for this observation. While we agree that 'cellular neighborhood' involves computational methods like graph-based networks or k-nearest neighbor clustering, in our opinion the term has become a standard biological descriptor in the spatial proteomics field. Specifically, the landmark work by Schürch et al. (Cell, 2020) established the term for the recurring local assemblages of cell types that coordinate tissue function. In our manuscript, we use the term to describe the composition of cells surrounding a central cell, which is distinct from the more functional connotation of a 'niche.'

(6) Please specify the total number of single cells analyzed by the scDVP pipeline. At line 400 or so, the authors mention that "50 single immune cells" were analyzed, but it remains unclear whether these 50 cells were included in the comparison with the other methods.

We clarified that for immune cells we only captured very few good-quality proteomes (judged by depth) due to the technical challenge isolating small cells and the limited proteomic input material. Only those three cells that passed the QC filter (400 proteins) were exemplarily shown as part of **Fig. 5b**, and otherwise not used for any further analysis. In our experience, immune cell analysis by scDVP requires further advances in sample processing and MS sensitivity.

Reviewer #3 (Remarks to the Author):

I co-reviewed this manuscript with one of the reviewers who provided the listed reports. This is part of the Nature Methods initiative to facilitate training in peer review and to provide appropriate recognition for Early Career Researchers who co-review manuscripts.

Reviewer #4 (Remarks to the Author):

The authors have substantially revised the manuscript and added new data.
In doing so most comments of the reviewers were addressed and the clarity of the manuscript was improved.

We thank the Reviewer for the constructive feedback and are pleased to hear that our manuscript improved.

One additional minor comment that was missed in my initial review is that the authors state that they use low bind 384 plates. The part number then shows that they are using DNA low bind tubes rather than protein low bind tubes. This should be stated in the text.

We clarified this in the Methods section.

Reviewer #4 (Remarks on code availability):

Not reviewed in the revised version.
